# Neutrophils Dominate the Cervical Immune Cell Population in Pregnancy and Their Transcriptome Correlates With the Microbial Vaginal Environment

**DOI:** 10.3389/fmicb.2022.904451

**Published:** 2022-06-14

**Authors:** Amirah Mohd Zaki, Alicia Hadingham, Flavia Flaviani, Yasmin Haque, Jia Dai Mi, Debbie Finucane, Giorgia Dalla Valle, A. James Mason, Mansoor Saqi, Deena L. Gibbons, Rachel M. Tribe

**Affiliations:** ^1^Department of Women and Children’s Health, School of Life Course and Population Sciences, Faculty of Life Sciences and Medicine, King’s College London, London, United Kingdom; ^2^NIHR Biomedical Research Centre, Guy’s and St. Thomas’ NHS Foundation Trust and King’s College London, London, United Kingdom; ^3^Peter Gorer Department of Immunobiology, School of Immunology and Microbial Sciences, Faculty of Life Sciences and Medicine, King’s College London, London, United Kingdom; ^4^Institute of Pharmaceutical Science, School of Cancer and Pharmaceutical Science, Faculty of Life Sciences and Medicine, King’s College London, London, United Kingdom

**Keywords:** neutrophils, host response, vaginal, RNA sequencing, metagenomics, microbiome, pregnancy, preterm birth

## Abstract

The cervicovaginal environment in pregnancy is proposed to influence risk of spontaneous preterm birth. The environment is shaped both by the resident microbiota and local inflammation driven by the host response (epithelia, immune cells and mucous). The contributions of the microbiota, metabolome and host defence peptides have been investigated, but less is known about the immune cell populations and how they may respond to the vaginal environment. Here we investigated the maternal immune cell populations at the cervicovaginal interface in early to mid-pregnancy (10–24 weeks of gestation, samples from *N* = 46 women), we confirmed neutrophils as the predominant cell type and characterised associations between the cervical neutrophil transcriptome and the cervicovaginal metagenome (*N* = 9 women). In this exploratory study, the neutrophil cell proportion was affected by gestation at sampling but not by birth outcome or ethnicity. Following RNA sequencing (RNA-seq) of a subset of neutrophil enriched cells, principal component analysis of the transcriptome profiles indicated that cells from seven women clustered closely together these women had a less diverse cervicovaginal microbiota than the remaining three women. Expression of genes involved in neutrophil mediated immunity, activation, degranulation, and other immune functions correlated negatively with *Gardnerella vaginalis* abundance and positively with *Lactobacillus iners* abundance; microbes previously associated with birth outcome. The finding that neutrophils are the dominant immune cell type in the cervix during pregnancy and that the cervical neutrophil transcriptome of pregnant women may be modified in response to the microbial cervicovaginal environment, or vice versa, establishes the rationale for investigating associations between the innate immune response, cervical shortening and spontaneous preterm birth and the underlying mechanisms.

## Introduction

The upper vaginal tract and ectocervix are lined with stratified squamous epithelium that produce a mucosal barrier and cervicovaginal fluid rich in host defence peptides, metabolites, and immune cells. This forms a first line of defence against opportunistic pathogens. In addition, commensal bacteria such as *Lactobacillus* species live in a mutualistic relationship with the host, and by producing lactate they create an acidic vaginal environment (Smith and Ravel, 2017). *Lactobacillus* species act to suppress anaerobes like *Gardnerella*, *Streptococcus*, *Prevotella*, *Ureaplasma* and others related to infections such as bacterial vaginosis. The vaginal microbiota, examined *via* 16S rRNA sequencing, is often classified into community state types (CSTs) based on which species dominates the microbiome, i.e., *Lactobacillus crispatus* (CST I), *Lactobacillus gasseri* (CST II), *Lactobacillus iners* (CST III), *Lactobacillus jensenii* (CST V) and CST IV (diverse mixture of anaerobic bacteria); with recent studies based on metagenomics sequencing expanding these groups (Mancabelli et al., 2021).

The vaginal environment of pregnant women is enriched in *Lactobacillus* species compared to non-pregnant women (Gupta et al., 2011; Aagaard et al., 2012; Freitas et al., 2018) and the abundance of *Lactobacillus* tends to increase as pregnancy progresses (Romero et al., 2014; Kindinger et al., 2017; Flaviani et al., 2021). Several studies have linked a diverse non-*Lactobacillus* dominant vaginal microbiome and preterm birth (DiGiulio et al., 2015; Callahan et al., 2017; Brown et al., 2018) although ethnic differences may exist (Manuck, 2017; Fettweis et al., 2019; Flaviani et al., 2021). An imbalance can have a negative impact on the host, leading to cervical shortening, inflammation, and greater risk of ascending inflammation which is associated with spontaneous preterm birth (sPTB; Amabebe et al., 2016; Elovitz et al., 2019; Fettweis et al., 2019; Flaviani et al., 2021; Pruski et al., 2021). Changes in bacterial communities are also accompanied by alteration in host defence peptides and metabolic profiles (Amabebe et al., 2016; Elovitz et al., 2019; Flaviani et al., 2021; Pruski et al., 2021) but there has been less research into the relationship with local immune cells. The vaginal microenvironment hosts a number of immune cells (such as neutrophils, dendritic cells, macrophages, NK cells and T cells) expressing receptors, e.g., toll-like receptors (TLRs) and nod-like receptors (NLRs), which recognise the presence of pathogenic microbial products (Benjelloun et al., 2020). The relationship between the microbiome and the immune response in sPTB is being actively explored, although to date, studies have mainly focused on the associations between soluble immune mediators and the microbiome in sPTB (Elovitz et al., 2019; Fettweis et al., 2019; Flaviani et al., 2021; Florova et al., 2021). Hunter and colleagues have investigated cervical leukocytes and reported that the absence of cervical macrophages and low C–C motif chemokine ligand 2 (CCL2) may be associated with sPTB (Hunter et al., 2016). However, the contribution of other immune cells and their interaction with the cervicovaginal microbiome are still poorly understood even in healthy pregnancies, particularly in relation to neutrophils, which are considered the most abundant leucocyte population in the cervicovaginal environment (Hunter et al., 2016; Farr et al., 2020) and warrant further investigation.

Research outside of the reproductive tract has demonstrated circulating neutrophil phenotype heterogeneity, and that products derived from microbiota can induce neutrophil diversity (Clarke et al., 2010; Nauseef and Borregaard, 2014; Zhang et al., 2015). Such studies have generally relied on the assessment of freshly isolated neutrophils due to the significant challenges in preserving this cell type. Short half-life in the blood stream (~ 6–8 h), rapid apoptosis after purification from the blood, tissue or other biological fluids (Scheel-Toellner et al., 2004; Summers et al., 2010), and difficulties in the cryopreservation of neutrophils (Coffelt et al., 2016) makes the analysis of this cell type complex. Furthermore, studies of cervical neutrophils and cervical lymphocytes have often been performed using neutrophils extracted from tissue biopsies (Scheel-Toellner et al., 2004; Summers et al., 2010). In pregnancy, cervical biopsies cannot easily be obtained, so the main approach used is cervical cytobrush sampling, which can only provide a limited number of cells for analysis.

Here, we describe a combined cell enrichment approach for neutrophils for RNA sequencing (RNA-seq) to overcome this limitation. We interrogated the relationship between the host immune cells, particularly neutrophils, and the cervicovaginal microbiome in early to mid-pregnancy with the goal to provide insight into how these interactions may influence inflammation and hence potentially risk of sPTB.

## Materials and Methods

### Participant and Sample Collection

Pregnant women were selected from an ongoing pregnancy study cohort (INSIGHT) as part of a prospective longitudinal observational study of women at high and low risk of spontaneous preterm labour (*N* = 46). This study uses samples obtained predominantly from high-risk women (10–24 weeks of gestation) as defined by the following criteria: one or more of prior sPTB or late miscarriage between 16–36^+6^ weeks’ gestation, previous cervical surgery, uterine anomaly or incidental finding of a cervical length < 25 mm on transvaginal ultrasound scan. Low-risk women were defined as women that did not meet the criteria for high-risk. Women were recruited either from the Preterm Surveillance Clinic or the general antenatal clinic at Guys and St Thomas’ NHS Foundation Trust (Hezelgrave et al., 2020).

All women (Table 1) provided samples for flow cytometry to assess immune cell profiles across gestation. For the comparison of neutrophil transcriptome and cervical microbiota samples a subset of these women (*N* = 10) women were successfully sequenced and analysed.

**Table 1:** Demographics and clinical characteristic of participants in the study cohort.

	Term (*N* = 40)	Preterm (*N* = 6)	Overall (*N* = 46)
**[*n*** **(%)]**			
**Ethnicity**			
African	7 (17.5)	4 (66.7)	11 (23.9)
Black-Caribbean	3 (7.5)	1(16.7)	4 (8.7)
White European	26 (65.0)	1 (16.7)	27 (58.7)
Unclassified	4 (10.0)	0 (0)	4 (8.7)
**Age (years)**			
20–30	5 (12.5)	0 (0)	5 (10.9)
30–40	32 (80.0)	6 (100)	38 (82.6)
40–50	3 (7.5)	0 (0)	3 (6.5)
**BMI (kg/m** ^**2**^ **)**			
Underweight (BMI < 18)	1 (2.5)	0 (0)	1 (2.2)
Healthy weight (18.5 ≥ BMI ≤ 24.9)	21 (52.5)	1 (16.7)	22 (47.8)
Overweight (25 ≥ BMI ≤ 29.9)	11 (27.5)	3 (50)	14 (30.4)
Obese (30 ≥ BMI ≤ 39.9)	7 (17.5)	1 (16.7)	8 (17.4)
Morbidly obese (BMI > 40)	0 (0)	1 (16.7)	1 (2.2)
**PPROM during current pregnancy**			
Unknown	7 (17.5)	0 (0)	7 (15.2)
No	27 (67.5)	4 (66.7)	31 (67.4)
Yes	6 (15)	2 (33.3)	8 (17.4)
**Smoking**			
Current	1 (2.5)	0 (0)	1 (2.2)
Ex—gave up before pregnancy	6 (15)	0 (0)	6 (13)
Never	33 (82.5)	5 (83.3)	38 (82.6)
Unknown	0 (0)	1 (16.7)	1 (2.2)
**Preterm birth risk status**			
High risk	36 (90)	6 (100)	42 (91.3)
Low risk	4 (10)	0 (0)	4 (8.7)

BMI, body mass index, PPROM, preterm premature rupture of membranes.

Five women in the cohort received progesterone during their pregnancy, but only two women were on progesterone at the time of sampling. Only one woman was on progesterone at the time of sampling in the microbiota and neutrophil RNA-seq subset). During speculum examination, a cervicovaginal swab was obtained from the posterior fornix using a nylon flocked swab (Copan eSwab, VWR International Ltd., United Kingdom) for shotgun metagenomic sequencing, placed into 1 ml of TE buffer (Promega, United Kingdom) transported immediately on ice to the laboratory. Swab was removed and TE buffer were stored at −80°C until analysis. Cervical cytobrushes for immune cells were obtained by placing the cytobrush (VWR International Ltd., United Kingdom) into the endocervical os and rotating for 360° for 5 s, placed in RPMI medium (Life Sciences UK Ltd.) and transported immediately on ice to the laboratory for processing.

### Flow Cytometry Analysis and Isolation of Cervical Immune Cells

The 15 ml tube containing the cytobrush sample was vortexed for 1 min, and centrifuged at 200x g for 10 min at 4°C. Supernatant was divided into aliquots and stored at −80°C. The cell pellet was resuspended in 2 ml PBS containing 1% fetal calf serum and passed through a 70 μm filter. Cells were centrifuged at 200 x g for 5 min and resuspended at 10^7^/ml. Cells were phenotyped using flow cytometry. Zombie NIH was used to stain squamous epithelial cells and dead leukocytes. Cells excluded from Zombie dye were examined using directly labelled monoclonal antibodies against the following human proteins: CD45-APC (clone HI30), CD3-AF700, CD16-PECY7, CD14-BV650, CD19-BV786 and HLADR (AF488)-PE, CD66b-FITC from BioLegend. A minimum of 200,000 CD45+ cells were collected, and data were analysed using FlowJo (v10.7.1, BD). Cells were gated on live CD45+ prior to hierarchical gating on T cells, B cells, NK cells, neutrophils and monocytes. Wilcoxon rank sum tests were performed to test whether immune cell proportions differed by pregnancy outcome (term versus preterm). Univariate linear regression models were created to test whether immune cell proportions changed according to when the samples were taken in pregnancy.

For isolation of cervical neutrophils *via* fluorescence-activated cell sorting (FACS), stained cells were analysed and sorted using BD FACS Aria II Cell Sorter (BD). Cervical neutrophils were sorted to > 98% purity and were lysed into lysis buffer (Qiagen) and 2-mercaptoethanol (Merck, United Kingdom) and kept at −80°C until further analysis.

A total of 54 samples were processed, but *n* = 3 were predominantly dead cells; these samples were excluded from further analysis. Results are reported for *n* = 51 samples from *N* = 46 women.

### DNA Isolation

DNA was extracted from thawed samples using QIAmp DNA Mini Kit (Qiagen) with some modification from the standard protocol. Briefly, upon thawing samples on ice, samples were treated for enzymatic cell lysis with lysozyme (10 mg/ml), mutanolysin (25,000 U/ml), lysostaphin (Merck, United Kingdom; 4,000 U/ml in sodium citrate) and TE50 buffer (10 nM Tris HCL and 50 nM EDTA, pH 8.0), then incubated for 1 h at 37°C (Aagaard et al., 2012). The resulting lysate was processed using QIAmp DNA Mini Kit according to the manufacturer’s recommendation. DNA was eluted twice in 100 μl of the kit buffer labelled AE. DNA was stored at −80°C until analysis.

### Shotgun Metagenomic Sequencing

Fully anonymised samples of DNA were transported on dry ice (with continuous temperature monitoring) to the sequencing provider. Extracted DNA was fragmented by sonication to yield 200–500 bp fragments. The shotgun metagenomic sequence libraries were constructed using end-repair, dA-tailing, adapter ligation and PCR amplification. Quality of DNA libraries was assessed on the Agilent Bioanalyzer 2,100 system (Agilent Technologies). Whole genome shotgun sequencing was performed on the BGISEQ-500 platform (100 bp of paired end reads; Fang et al., 2018).

### Neutrophil RNA Sequencing

RNA was isolated using RNesay Mini Kit (Qiagen). Quality of RNA was assessed on the Agilent Bioanalyzer 2,100 system (Agilent Technologies). Fully anonymised samples were shipped to the sequencing provider on dry ice for library construction using SMART-Seq II protocol for ultra-low input RNA sample on BGISEQ-500 platform. Quality of library was assessed on the Agilent Bioanalyzer 2,100 system (Agilent Technologies).

### Neutrophil Gene Expression Data Analysis

Reads were mapped to the human reference genome (hg38_tran) using HISAT2 v2.1.0 (Kim et al., 2019) using paired alignments. Read fragments were summarised and counted over genes using featureCounts v2.0.0 (Liao et al., 2014) and only read pairs with both ends aligned were counted.

Raw counts were batch adjusted, to remove the impact of sequencing lane, using the ComBat_seq function in the R package sva v3.36.0 (Zhang et al., 2020), with ethnicity labelled as the biological condition of interest. Principal component analysis (PCA) was performed on the regularized log (rlog) transformed adjusted count data using the R package DESeq2 v1.28.1 (Love et al., 2014). The pairwise Pearson’s correlations of the rlog transformed count data of the samples was performed using stats v4.1.0 (R Core Team, 2021) and plotted using pheatmap v1.0.12 (Kolde, 2019). Clustering of samples to find the optimum number of clusters in our cohort was performed using hierarchical clustering and k-means clustering of the rlog transformed gene expression, using the R packages stats (R Core Team, 2021) and cluster v2.1.2 (Maechler et al., 2021). All statistical analysis was performed using R v4.1.0 unless otherwise stated.

### Differential Gene Expression Analysis

Differential expression analysis was performed using DESeq2 (Love et al., 2014) on the adjusted counts of genes which were present in at least one sample. The adjustment removed the impact of sequencing lane, while maintain the data as appropriate input for DESeq2 (Zhang et al., 2020). The differentially expressed genes were determined between the samples that clustered closely in Cluster 1 (*N* = 7) and the remaining samples (*N* = 3). *p*-values were adjusted using FDR (false discovery rate) correction (Benjamini and Hochberg, 1995) with a cut-off of *p* < 0.05 and a fold change (FC) ≥ |1.5|. The log FC were then shrunk using an Empirical Bayes approach from the R package ashr v2.2–47 (Stephens, 2016). The volcano plot of differentially expressed genes was made using the R package EnhancedVolcano v1.10.0 (Blighe et al., 2021).

The differential expressed genes with their adjusted *p*-values and log fold changes were inputted into QIAGEN Ingenuity Pathway Analysis (IPA; Krämer et al., 2014; QIAGEN Inc., 2021) to examine the pathway results. The parameters used in IPA included setting the reference set to IKB (Genes + Endogenous Chemicals); selecting both direct and indirect relationships; using the default settings on the network, node type, data source and confidence options; selecting the species as Human; and selecting immune cells as the tissue type.

Gene ontology (GO) analysis was performed using the R package gage v2.42.0 (Luo et al., 2009). GO analysis was run using the log FC of the differentially expressed entrez genes and using data from The Gene Ontology Consortium in the R package gageData v2.30.0 (Wickham, 2016; Grant, 2018; Luo, 2021; The Gene Ontology Consortium, 2021). The analysis of biological process, molecular function and cellular component GO were run separately. The GO terms which were statistically significantly (FDR adjusted *p* < 0.05) upregulated or downregulated by transcriptomics cluster were explored and plotted using ggplot2 v3.3.3 (Wickham, 2016; McIver et al., 2018).

### Metagenome Analysis

After sequencing, the raw reads were filtered in order to remove adapters, contaminants and low-quality reads (Supplementary Table 1). Removal of human DNA contamination was performed using Kneaddata v0.7.4 (McIver et al., 2018; Ma et al., 2020) which allowed merging of paired end reads. Taxonomical and functional profiling was performed using VIRGO non-redundant gene catalogue and tool (Ma et al., 2020; Oksanen et al., 2022). The alpha diversity of each sample was calculated using the R package vegan v2.5–7 (Langfelder and Horvath, 2012; Oksanen et al., 2022), using Shannon’s index on the metagenome counts. A Wilcoxon rank sum test was performed on the alpha diversity indexes to test for a pairwise comparison between the group of samples in transcriptomics Cluster 1 versus Clusters 2–4.

### Weighted Gene Correlation Network Analysis

Weighted gene correlation network analysis (WGCNA) was used to cluster genes from the rlog transformed cervical neutrophil gene expression data to identify genes which are highly correlated across the samples and group them into modules, each labelled with a colour, using WGCNA’s unsupervised algorithm (Langfelder and Horvath, 2008, 2012). Parameters were set up to include a signed network, a soft thresholding power of 18 and a minimum module size of 30 genes. Modules with a correlation > 0.75 between their module eigengenes were merged.

Associations were then determined between module eigengenes, which represent each module, and selected microbiota taxa and other maternal quantitative clinical data, using Pearson’s correlations (*p* < 0.05). The GO terms characterising the modules were calculated using the anRichment v1.20 (Langfelder, 2021). Modules with GO terms that had Bonferroni-corrected *p*-values ≤ 0.05 and were associated with microbiota and clinical traits of interest were examined.

### Study Approval

The INSIGHT study was approved through by the NHS Human Research Authority (HRA), London—City and East Research Ethics Committee (13/LO/1393). Informed written consent was obtained from all participants.

## Results

### Immune Cells in the Cervix of Women at High Risk of Delivering Preterm

**Figure 1:**
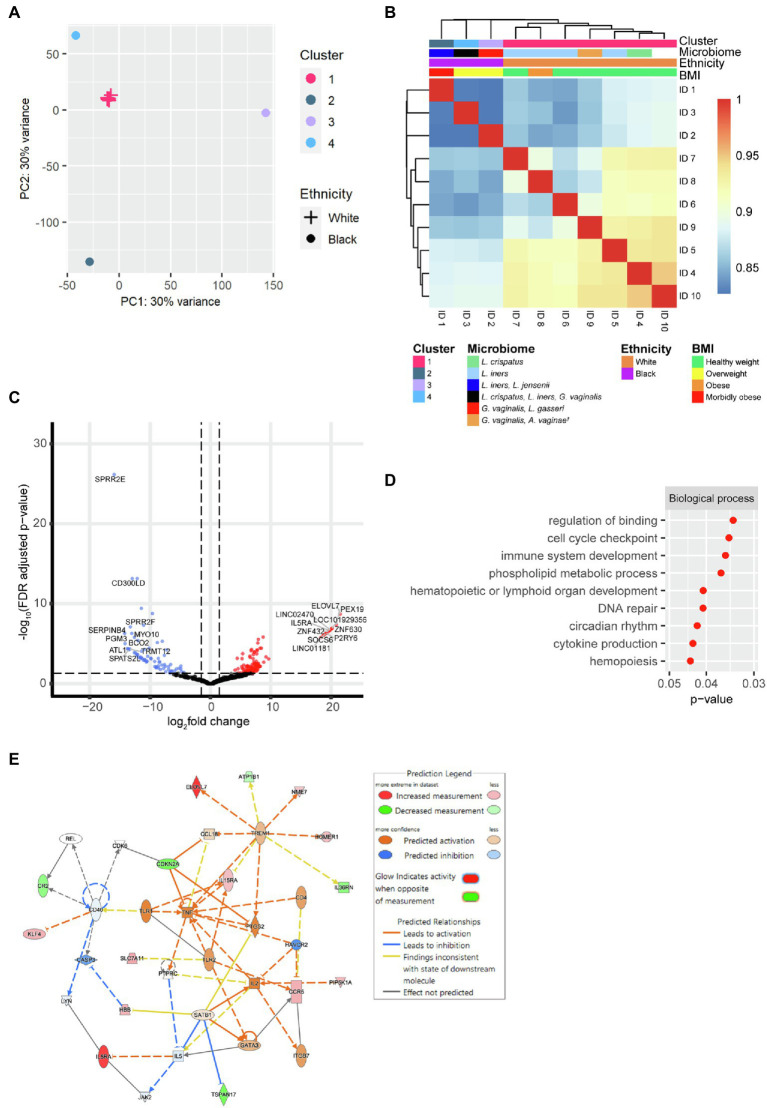
Cross sectional analysis of immune cells present in cytobrush samples **(A)** Representative flow cytometry gating strategy on single cell suspension from a cytobrush sample taken at 10–24 gestation. Cells were gated on live CD45+ prior to hierarchical gating on T cells, B cells, NK cells, neutrophils and monocytes. **(B)** Mean proportion of CD45+ immune cells in the cervicovaginal environment (*n* = 51 samples from *N* = 46 women). **(C)** Immune cell proportions from cytobrush samples by term (*N* = 40) and preterm (*N* = 6) outcome. There was no statistical difference in immune cell proportions by pregnancy outcome (Wilcoxon rank sum tests). **(D)** Immune cell profile across gestation (sampling window from 10 to 24 weeks’, *n* = 51 samples from *N* = 46 women). *p* values were assessed by linear regression (^*^p < 0.05).

In this cross-sectional study of women at high risk of delivering preterm, as described in Table 1, epithelial cells were the dominant cell type found in cytobrush samples (10–24 weeks gestation) from all women, and neutrophils were the dominant immune cell (Figures 1A,B). Following gating for CD45 + cells and labelling with different surface markers, CD66b^+/high^ neutrophils were readily detected in all samples (*n* = 51) with variable levels of CD56^+^ NK cells (39/51), CD19^+^ B cells (22/51), CD3^+^ T cells (15/51) and HLA-DR^+^ monocytes (33/51). Samples were analysed prior to pregnancy outcome being known but no significant differences in the percentage of neutrophils (*p* = 0.42), NK cells (*p* = 0.4), B cells (*p* = 1), T cells (*p* = 0.078), patrolling monocytes (*p* = 0.87), intermediate monocytes (*p* = 0.94) or classical monocytes (*p* = 0.82) were observed in cervical samples from women who delivered preterm (*n* = 6) or at term (*n* = 40; Figure 1C). However, there were significant differences in the proportion of neutrophils (*p* = 0.0303, coefficient = −1.45) and T cells (*p* = 0.0165, coefficient = 0.180) depending on gestational age (Figure 1D).

### Gene Expression in Cervical Neutrophils

**Figure 2:**
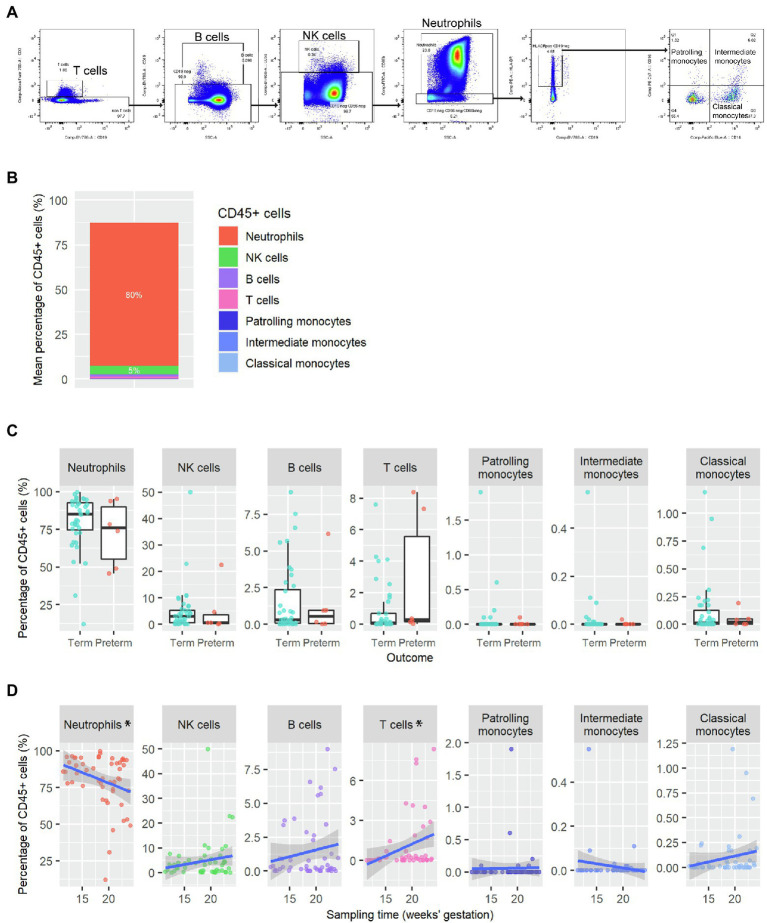
Gene expression of cervical neutrophils (*N* = 10). **(A)** Principal component analysis (PCA) of the transcriptional profile of cervical neutrophils. Cluster 1 has a membership size of 7 and Clusters 2–4 are composed of one woman each. **(B)** Pearson’s pairwise correlations were calculated between all samples with a cluster dendrogram showing the hierarchical clustering between samples. **(C)** Volcano plot of differential gene expression of samples in Cluster 1 versus samples in Clusters 2–4. The 127 significantly upregulated genes (fold change ≥ 1.5, FDR < 0.05) are shown in red and the 73 significantly downregulated genes (fold change ≤ −1.5, FDR < 0.05) are shown in blue. The top ten upregulated and top ten downregulated genes are labelled. **(D)** GO analysis of the differentially expressed genes between samples in Cluster 1 versus samples in Clusters 2–4. Selected GO terms which were significantly upregulated in Cluster 1 are shown. **(E)** IPA network analysis of differentially expressed genes of samples in Cluster 1 versus Clusters 2–4. Statistically significantly differentially expressed genes (fold change ≥ |1.5|, value of *p* < 0.05) which are upregulated or downregulated in Cluster 1 are shown in red and green, respectively. Orange indicates predicted activation and blue indicated predicted downregulation of genes and pathways. Solid lines indicate direct pathway, whilst dotted lines indicate indirect pathways. ^†^ *Atopobium vaginae* is also known as *Fannyhessea vaginae* as per new nomenclature.

As neutrophils were the dominant leucocyte population detected, neutrophils were sorted from *N* = 10 samples for bulk RNA-seq. Both *k*-means clustering silhouette analysis and hierarchical clustering with Euclidean distance and complete linkage of the rlog gene expression, indicated that the transcriptional profiles clustered into an optimal four clusters (*k* = 4). Cluster 1 comprised data from seven women (including one woman on progesterone), and the remaining three clusters had a membership size of one each. The samples in Cluster 1 clustered closely in PCA, while the three remaining samples showed a more distinct transcriptional profile to each other (Figure 2A). The clustering was associated with ethnicity, BMI and the microbiome (Figure 2B). Cluster 1 was composed of samples from White women with lower BMIs (healthy (18.5 ≥ BMI ≤ 24.9) *N* = 6, obese (30 ≥ BMI ≤ 39.9) *N* = 1), while Clusters 2–4 comprised samples from Black women with higher BMIs (overweight (25 ≥ BMI ≤ 29.9) *N* = 2, morbidly obese (BMI > 40) *N* = 1). The clustering was also associated with different alpha diversity scores of the cervicovaginal microbiome, where the women in Cluster 1 had a significantly lower Shannon’s diversity index than the women in Clusters 2–4 (*p* = 0.024; Supplementary Figure 1A). The difference in neutrophil RNA expression between the samples that clustered together in Cluster 1 (*N* = 7) versus the remaining three samples in Clusters 2–4 were then examined.

There were 200 differential expressed genes in the cervical neutrophils when comparing samples in Cluster 1 versus the samples in Clusters 2–4 (FDR *p* < 0.05, FC ≥ |1.5|). A total of 127 genes were upregulated and 73 were downregulated (Figure 2C and Supplementary Table 2). SOCS6, IL5RA and P2RY6 (suppressor of cytokine signalling 6, interleukin 5 receptor subunit alpha and pyrimidinergic receptor P2Y6 respectively) were amongst the top ten most upregulated genes by fold change in samples in Cluster 1. CD300LD (CD300 molecule like family member d) was amongst the top ten most downregulated in samples in Cluster 1 by fold change.

Gene ontology analysis of the differentially expressed genes suggested that cytokine production and immune cell development pathways were significantly upregulated in the neutrophils of samples in Cluster 1 (Figure 2D). IPA network analysis predicted women in Cluster 1 would have an increased activation of TNF, IL2, IL5 and TLR1 compared to samples in Clusters 2–4 (Figure 2E).

### Interactions Between the Cervical Neutrophil Transcriptome, Cervicovaginal Metagenome and the Quantitative Clinical Data

**Figure 3:**
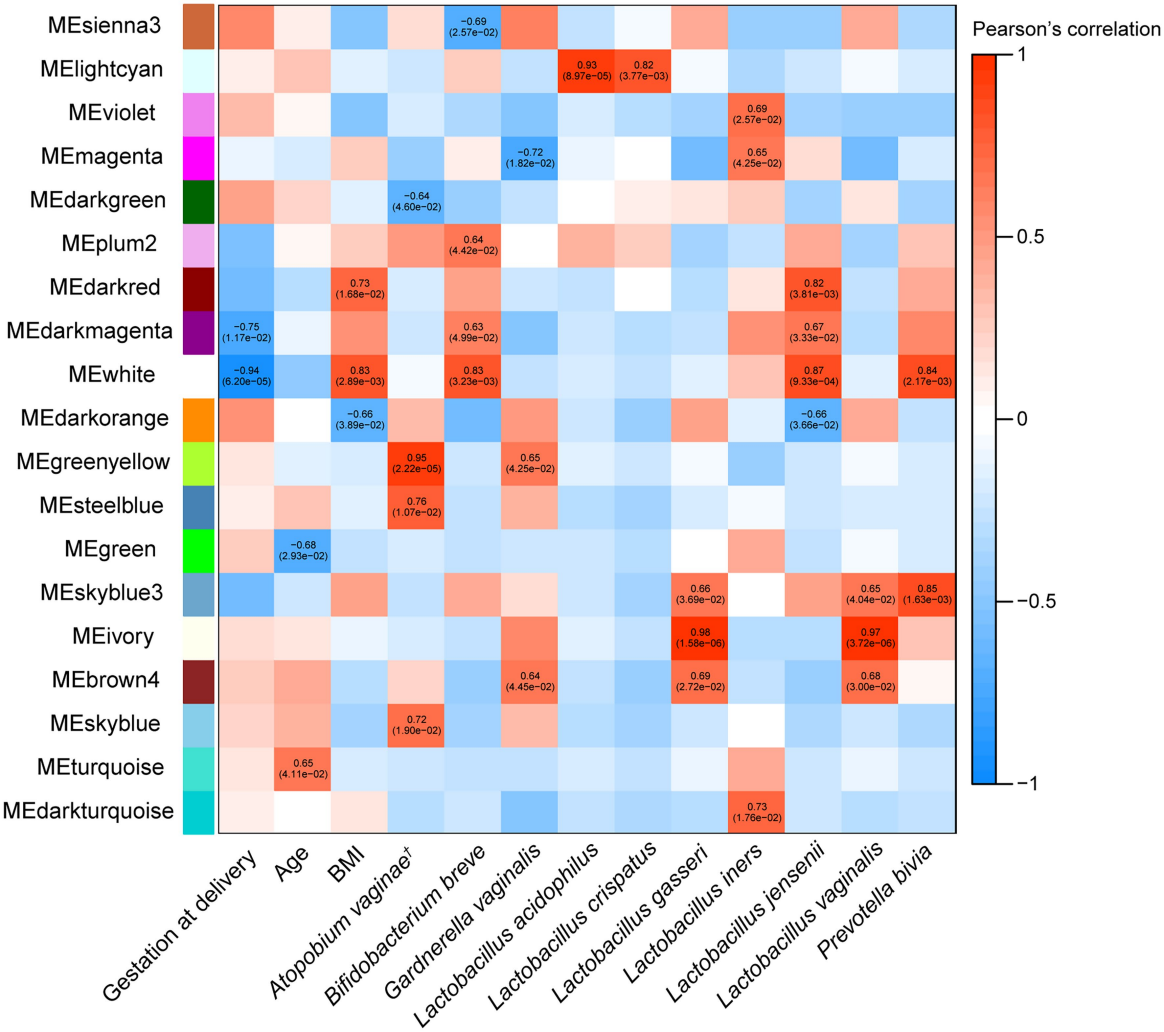
Weighted gene correlation network analysis (WGCNA) heatmap of correlations between module eigengenes (MEs), which represent each cluster of expressed cervical neutrophil genes, and microbiota taxa relative abundance and other maternal quantitative clinical data in all women (*N* = 10). Significant Pearson correlations (*p* < 0.05) between MEs and traits have text with the correlation value, followed by the *p*-value in brackets. Modules with no correlations between their MEs and microbiome or clinical data are not shown. The list of gene in each module is in Supplementary Table 3. ^†^ *Atopobium vaginae* is also known as *Fannyhessea vaginae* as per new nomenclature.

Shotgun metagenomic sequencing allowed investigation of the associations between cervicovaginal microbiota species and neutrophil gene expression (*N* = 9). WCGNA revealed statistically significant associations between 19 out of 45 module eigengenes (representing clusters/modules of genes encoding for the expressed neutrophil mRNAs) and specific species from the cervicovaginal microbiota as well as some quantitative clinical data (Figure 3). *L. crispatus* and *L acidophilus* are associated with a lower risk of sPTB (Flaviani et al., 2021), and were both positively associated with the “light cyan” module eigengene. Gestational age at delivery was negatively correlated with the “dark magenta” and “white” module eigengenes, indicating that there could be an inverse relationship between pregnancy duration and the expression of these genes in cervical neutrophils.

**Figure 4:**
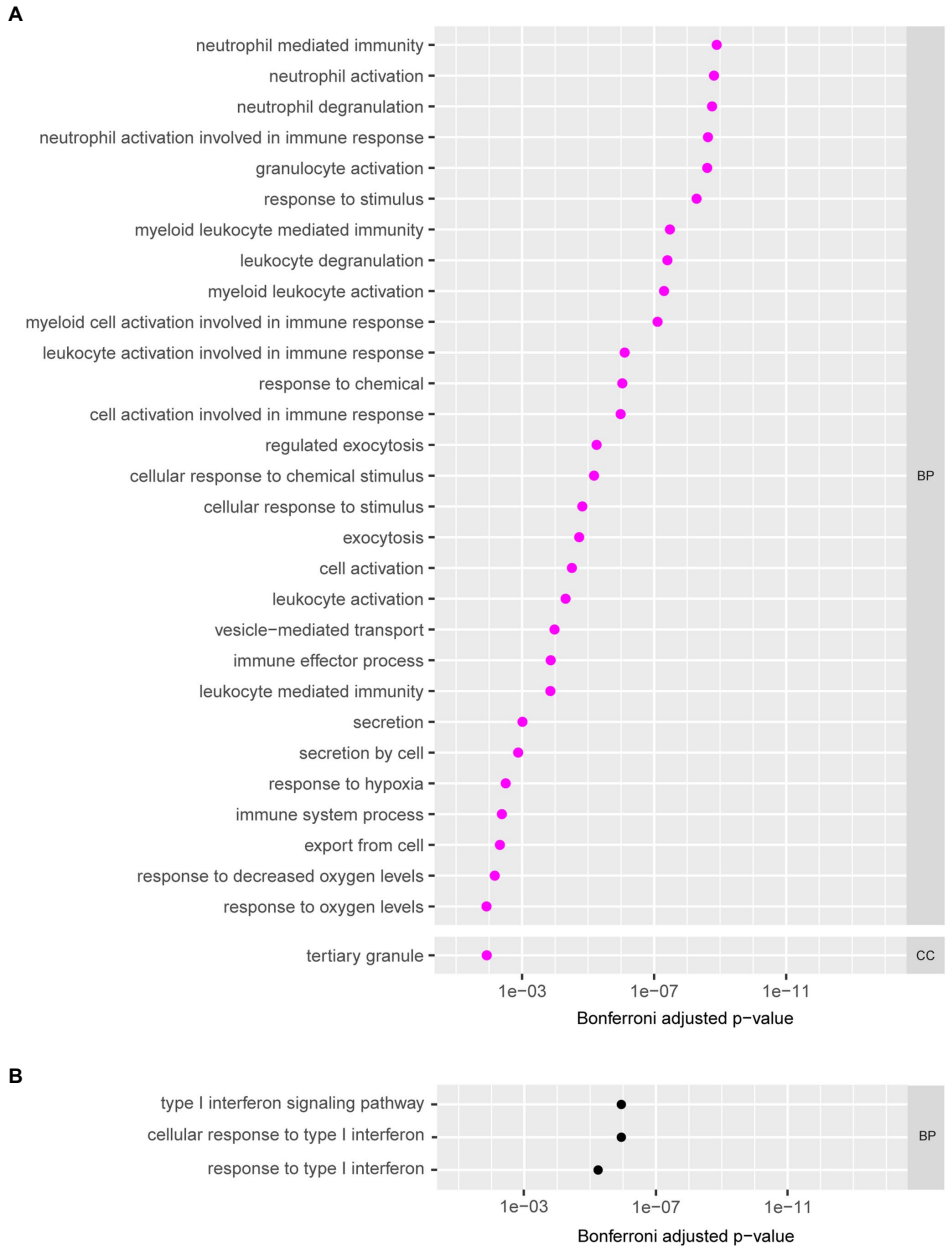
The gene ontology (GO) terms of the modules grouped according to biological process (BP), molecular function (MF) and cellular component (CC). **(A)** GO analysis of the genes in the “magenta” module of the WGCNA analysis on cervical neutrophil gene expression. Selected GO terms with Bonferroni corrected *p*-values (*p* ≤ 0.05) are shown. **(B)** GO analysis of the genes in the “ivory” module of the WGCNA analysis on cervical neutrophil gene expression. All GO terms with Bonferroni corrected *p*-values (*p* ≤ 0.05) are shown.

Gene ontology analysis was then performed on the expressed genes in the modules (Supplementary Table 3) to determine the functions and pathways associated with each module. Eight modules had GO terms associated with them that were significant (Bonferroni corrected *p* ≤ 0.05). Two out of eight of these modules (“magenta” and “ivory”) were associated with microbiota taxa or clinical data. GO analysis revealed the “magenta” module was composed of genes involved in neutrophil mediated immunity, neutrophil activation, neutrophil degranulation, and other immune pathways (Figure 4A). This “magenta” module eigengene was not associated with any quantitative clinical data, but it was negatively correlated with *G. vaginalis* (−0.72, *p* = 1.82 × 10^−2^) and positively correlated with *L. iners* (0.65, *p* = 4.25 × 10^−2^; Figure 3). The “ivory” module was associated with the type I interferon signalling pathway and response to type I interferon (Figure 4B), and this module eigengene was positively correlated to two variables: abundance of *L. gasseri* (0.98, *p* = 1.58 × 10^−6^) and *L. vaginalis* (0.97, *p* = 3.72 × 10^−6^; Figure 3).

## Discussion

Preterm birth is a complex disease and the identification of unifying risk factors are difficult to ascertain. This exploratory study examined the relationship between cervicovaginal neutrophil gene expression and cervicovaginal microbiota in paired samples. Profiling immune cells between 10 and 24 weeks of gestation identified neutrophils as the predominant subtype in cytobrush samples. Subsequent RNA-seq from FACS neutrophils from these samples revealed the presence of a distinct cluster of women with lower alpha diversity, lower BMI and of White ethnicity. Neutrophils that closely clustered together demonstrated upregulation of inflammatory signalling genes/pathways which is suggestive of neutrophil priming and a potentially more effective host response. Investigation of potential interactions between the host neutrophils and the cervicovaginal environment, revealed unique associations between cervical neutrophil gene expression and specific species within the resident microbiota.

Our finding, that neutrophils were the most abundant immune cell found in cytobrush samples, supports a previous reports from non-pregnant (Mohammadi et al., 2020) and pregnant women (Hunter et al., 2016); this differs from samples collected from biopsies (Trifonova et al., 2014). Neutrophils play important role in cervical ripening and can infiltrate the myometrium and cervix during labour (Osman et al., 2003; Rinaldi et al., 2014). Although our study was not specifically designed to examine differences in neutrophil proportions by pregnancy outcome, we did note that proportions were similar between samples that subsequently resulted in term and preterm birth. However, it is known that many women who deliver preterm exhibit more diverse microbiota profiles (Freitas et al., 2018; Fettweis et al., 2019) and several studies have shown that microbial products can influence circulating neutrophil phenotype (Clarke et al., 2010; Nauseef and Borregaard, 2014; Zhang et al., 2015; Xie et al., 2020). To this end we further examined the correlation between neutrophil gene expression and the vaginal microbiome in *ex vivo* samples.

To the best of our knowledge, this is the first report of RNA sequencing of an enriched cervical neutrophil cell population. Clustering of the RNA expression data showed that there was a cluster of women with similar expression profiles (*N* = 7) in Cluster 1, while the three remaining women had distinct neutrophil profiles; these two groups differed by BMI, microbiome and ethnicity. The top mRNAs that were significantly differentially expressed and upregulated in the neutrophils from women in Cluster 1 included SOCS6, IL5RA and P2RY6. SOCS6 has been shown to be upregulated in response to G-CSF and IL-2 in circulating neutrophils *in vitro* (Ratthé et al., 2007) and IL-5 receptor alpha (IL-5Rα) is reported to be expressed on neutrophils extracted from bronchoalveolar lavage of asthmatic children with inflammed lungs (Gorski et al., 2019). IL-5Rα has also been reported to be enhanced in lipopolysaccaride (LPS) primed neutrophils (Linch et al., 2012). An increase in IL-5 protein has also been associated with depletion in the abundance of *Lactobacillus, G. vaginalis* and *Ureaplasma* in the cervicovaginal microbiome (Campisciano et al., 2018). Pyrimidinergic receptor (P2RY6) gene activation leads to production of proinflammatory cytokines, IL-8 and IL-6 (Hao et al., 2014). Based on these significantly differentially expressed genes, pathway analysis predicted that TNF, IL-2, IL-5 and TLR1 were upregulated in women in Cluster 1. Together, these data suggested that neutrophils in women within Cluster 1 are potentially more mature and primed to suppress infection (Hsu et al., 2019; Mistry et al., 2019; Carissimo et al., 2020).

We hypothesised that the differences in neutrophil gene expression might be related to the resident microbiome (Zhang and Frenette, 2019). Innate immune cells including neutrophils that are constantly being exposed to various pathogens or commensal bacteria are able to adapt to the environment either to increase or decrease their responsiveness (Clarke et al., 2010; Deshmukh et al., 2014; Zhang et al., 2015) and subsequently shape their environment. Our attempts to further understand the interaction between neutrophils and cervicovaginal microbiota revealed that genes associated with neutrophil activation, degranulation and responses were negatively correlated with *G. vaginalis*, positively correlated with *L. iners,* and were not associated with maternal age or BMI. This suggests that bacteria can modify the host response within the vaginal environment or vice versa, as supported by previous studies (Beghini et al., 2013; Mehta et al., 2020; Florova et al., 2021). Metha et al. reported the associations between vaginal species such as *G. vaginalis* and *L. iners* using GWAS, with pathways associated with innate immune responses and neutrophil degranulation. Florova et al. identified an inverse relationship between *G. vaginalis* and the chemokine CXCL10 (Florova et al., 2021) while Beghini et al. identified the association between bacterial vaginosis (BV) and higher expression of vaginal neutrophil CD16 (which is associated with neutrophil maturity and activation; Beghini et al., 2013). Finally, a study using human vaginal epithelial cells, showed that *L. iners* directly induced pattern-recognition signalling activity in vaginal epithelial cells (Doerflinger et al., 2014), and this could also be true in neutrophils. There is also evidence that bacterial metabolites and other stimuli can influence neutrophil function, with much of the evidence originating from studies of the intestinal mucosa interface (Zhang and Frenette, 2019).

According to Zhang and colleagues the microbiota are a source of low-grade stimuli (e.g., LPS) that prime neutrophils *via* TLR/Myd88 pathways for vigorous response to inflammation (Zhang et al., 2015). Our data suggest prediction of upregulation in TLR pathway in Cluster 1 fits with this observation. Metabolites including short chain fatty acids produced by microbiota are also suggested to modulate neutrophil recruitment and activation (Doerflinger et al., 2014). It is possible that in less diverse microbiomes, such as the cervicovaginal immune system (Amabebe and Anumba, 2020), the microbiota (through the generation of short chain fatty acids and other metabolites) are also able to establish immune tolerance and prime neutrophils to be more mature and ready for robust innate and adaptive immune responses during infection. Indeed, in parallel studies, we have shown in pregnant human cervicovaginal fluid that there is a shift in metabolites depending on the dominant CST (Flaviani et al., 2021). The next step would be to determine how this specific shift in metabolites impacts neutrophil activation.

A major strength of our study is the focus on neutrophils, which are challenging to study due to the acknowledged fragility of this cell type if stored prior to analysis. Here we have demonstrated, in this prospective study, the feasibility of freshly isolating neutrophils from cervical samples from women at high risk of sPTB for RNA-seq. This is despite the relatively limited material that can be obtained using cytobrush sampling. To circumnavigate this, we successfully utilised a cell enrichment protocol for neutrophils prior to bulk RNA sequencing to mimic single cell approaches (Xie et al., 2020). The next step would be to optimise protocols that could overcome the limited cell numbers to allow for single cell RNA-seq. Our study was not without limitations, most notably the small number of participants and bias towards white Women with low BMI and Black women with higher BMI. As proof of principle, however, it was sufficient to detect different clusters of neutrophil RNA expression and correlate this with abundance of bacterial species within the vaginal environment. Going forward, we continue to prospectively collect samples from women to more fully evaluate phenotypic differences in early pregnancy immune cell profiles with pregnancy outcome (term versus preterm birth) and evaluation of protocols that allow for stabilisation and storage of cells prior to analysis. This would allow us to select samples on a case: control basis once pregnancy outcome is known.

In summary, our study reports a relationship between cervical neutrophils and specific microbiome species. This provides insight into how the vaginal environment is shaped by host–microbe interactions and potentially how a pro-inflammatory state can develop. The work herein also highlights the importance of further study of immune cell-microbe interactions in relation to cervical shortening and sPTB risk with a larger sample size.

## Data Availability Statement

The raw sequences are available at the European Nucleotide Archive (ENA) under accession number PRJEB51569. Further inquiries can be directed to the corresponding author.

## Ethics Statement

The studies involving human participants were reviewed and approved by NHS Human Research Authority (HRA), London City and East Research Ethics Committee (13/LO/1393). The participants provided their written informed consent to participate in this study.

## Author Contributions

AMZ and RMT conceived and designed the study with input from AJM and DG. AMZ, YH, and JM performed laboratory-based studies with input from DG. DF and GD supported recruitment and sample collection. AMZ analysed flow cytometry data. AH and FF undertook bioinformatics analysis with input from MS and RMT. AH and AMZ undertook statistical analysis. AMZ, AH, and RMT drafted the manuscript with input from all other authors. All authors contributed to the article and approved the submitted version.

## Funding

AH (Doctoral fellowship), AZ, FF, and MS are all funded by the NIHR Biomedical Research Centre (BRC) based at Guy’s and St. Thomas’ National Health Service Foundation Trust. Funding for the INSIGHT cohort that provided samples and data for this study was provided from Tommy’s Charity (Reg charity no. 1060508); NIHR Biomedical Research Centre (BRC) based at Guy’s and St. Thomas’ National Health Service Foundation Trust, the Rosetrees Trust (no. 298582, M303-CD1), Borne Foundation (no. 1167073) and Action Medical Research grant held by DG (GN2790).

## Author Disclaimer

The views expressed are those of the author(s) and not necessarily those of the NHS, the NIHR, or the Department of Health and Social Care.

## Conflict of Interest

The authors declare that the research was conducted in the absence of any commercial or financial relationships that could be construed as a potential conflict of interest.

## Publisher’s Note

All claims expressed in this article are solely those of the authors and do not necessarily represent those of their affiliated organizations, or those of the publisher, the editors and the reviewers. Any product that may be evaluated in this article, or claim that may be made by its manufacturer, is not guaranteed or endorsed by the publisher.
