# Supplementary material for: Neutrophils Dominate the Cervical Immune Cell Population in Pregnancy and Their Transcriptome Correlates With the Microbial Vaginal Environment
Source: Front Microbiol. 2022 Jun 14;13:904451. doi: 10.3389/fmicb.2022.904451 (PMC9237529; doi:10.3389/fmicb.2022.904451)
Supplement: Supplementary file 1 [file Data_Sheet_1.docx]

Supplementary Material

# Supplementary Figures and Tables

## Supplementary Figures


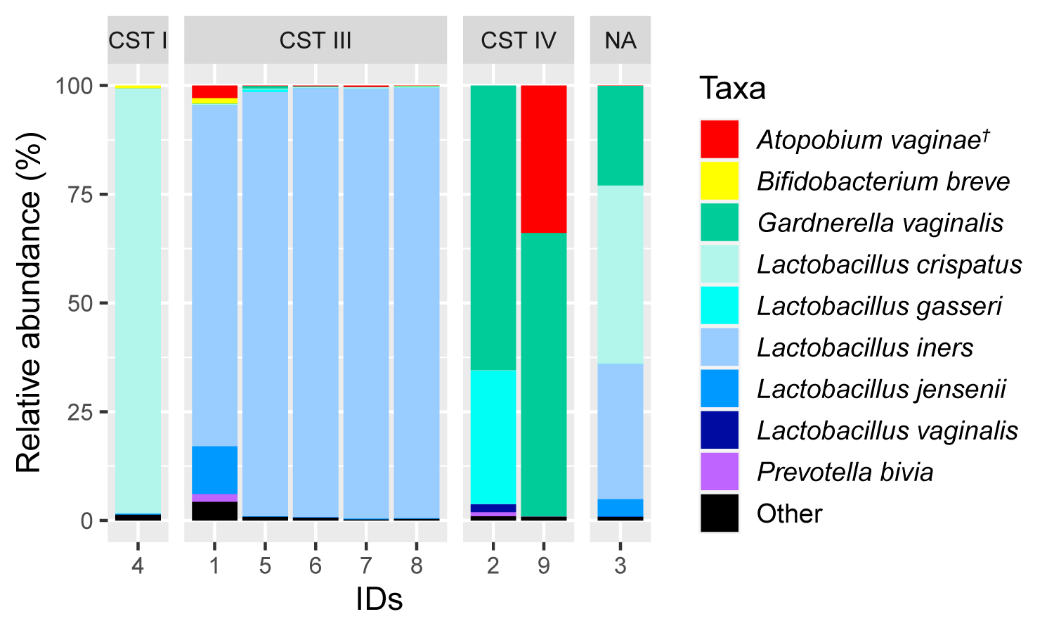

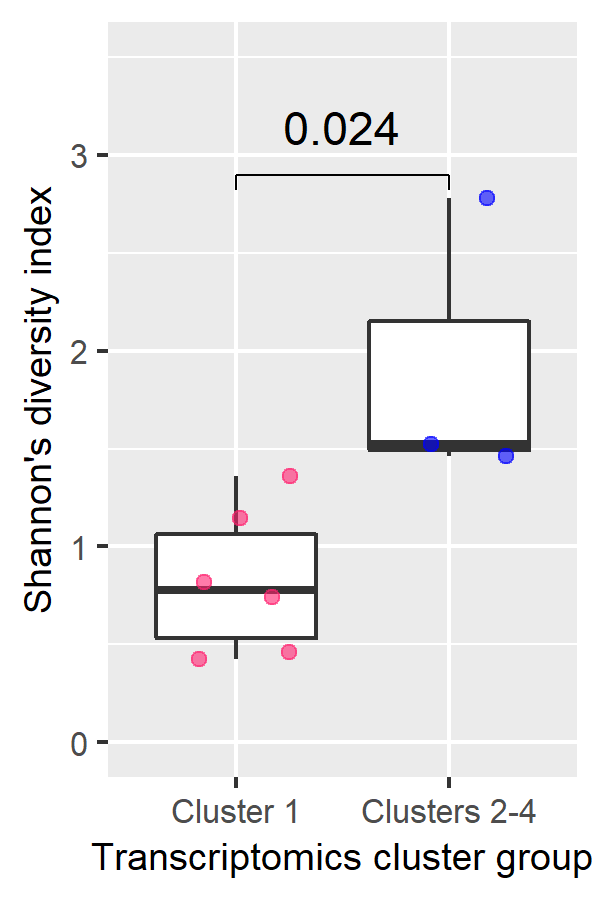


**A**

**B**

**Supplementary Figure 1:** Shotgun metagenomics of the vaginal microbiota (n=9) was performed on samples from the same women at same timepoint as cytobrush collection. (A) Alpha diversity scores, as calculated by Shannon’s diversity index by transcriptomics cluster group (p=0.024, Wilcoxon rank sum test). (B) Relative abundance of taxa present in each sample, grouped by community state type (CST). Taxa which were not present at >1% relative abundance in at least one sample have been grouped into an “Other” taxa category. The sample which does not fit into any CST category is labelled NA. ^†^ *Atopobium vaginae* is also known as *Fannyhessea vaginae* as per new nomenclature.

## Supplementary Tables

**Supplementary Table 1:** Metagenomic read counts

| Sample ID | Reads count from clean reads provided by BGI | Reads count after removal of Human reads with kneaddata |
| --- | --- | --- |
| ID_4 | 61845662 | 941879 |
| ID_5 | 61618802 | 1726226 |
| ID_9 | 61995584 | 1017000 |
| ID_6 | 61785680 | 385908 |
| ID_7 | 61676066 | 663590 |
| ID_1 | 61797246 | 1490087 |
| ID_2 | 61666698 | 1207138 |
| ID_8 | 61670304 | 625125 |
| ID_3 | 61507830 | 657434 |

**Supplementary Table 2:** Differentially expressed genes of Cluster 1 versus Clusters 2-4 (FDR p<0.05 and fold change (FC) ≥ |1.5|)

| Gene | log2FoldChange | padj |
| --- | --- | --- |
| ELOVL7 | 21.51647 | 2.01E-09 |
| PEX19 | 20.88268 | 4.32E-08 |
| ZNF630 | 20.19653 | 1.17E-07 |
| LOC101929356 | 20.12108 | 1.24E-07 |
| LINC02470 | 19.87564 | 1.82E-07 |
| IL5RA | 19.72674 | 2.22E-07 |
| ZNF432 | 19.54301 | 2.92E-07 |
| P2RY6 | 19.22803 | 4.89E-07 |
| SOCS6 | 19.06101 | 5.54E-07 |
| LINC01181 | 18.76475 | 8.80E-07 |
| MFAP3L | 18.74797 | 8.80E-07 |
| PIGN | 18.29699 | 1.67E-06 |
| HRH1 | 9.644565 | 7.80E-04 |
| UCHL5 | 9.218566 | 4.31E-04 |
| SLC7A11 | 8.842852 | 2.03E-03 |
| CSNK2A1 | 8.749569 | 1.46E-06 |
| DCK | 8.444332 | 3.51E-05 |
| PAX8-AS1 | 8.29161 | 6.54E-03 |
| CDK12 | 8.242851 | 3.51E-05 |
| CCP110 | 8.157669 | 7.46E-03 |
| DMTF1 | 8.101985 | 5.52E-03 |
| HECTD1 | 8.09629 | 7.29E-03 |
| GPN1 | 8.041913 | 7.08E-03 |
| ZNF136 | 7.955897 | 2.91E-03 |
| PLIN4 | 7.937423 | 2.78E-06 |
| KLF4 | 7.858974 | 4.65E-03 |
| ZGPAT | 7.857084 | 8.01E-06 |
| CCR5 | 7.850105 | 1.61E-02 |
| CHPT1 | 7.83026 | 3.86E-03 |
| LOC105375036 | 7.795871 | 7.56E-03 |
| ATP2C1 | 7.773293 | 2.94E-03 |
| HBB | 7.765819 | 1.82E-02 |
| ATP8A1 | 7.76511 | 7.06E-05 |
| RBBP4 | 7.756907 | 5.72E-05 |
| C3orf38 | 7.714774 | 6.92E-03 |
| LOC105377272 | 7.67663 | 5.52E-03 |
| MIS18BP1 | 7.674626 | 1.83E-02 |
| ZCCHC18 | 7.673598 | 1.78E-04 |
| NCR3LG1 | 7.655217 | 2.64E-02 |
| SND1 | 7.628114 | 3.73E-03 |
| SLC24A4 | 7.62081 | 5.54E-03 |
| CLINT1 | 7.600503 | 1.57E-03 |
| KPNA3 | 7.526156 | 5.65E-03 |
| TRMT6 | 7.495162 | 9.62E-03 |
| LOC107985869 | 7.468095 | 1.41E-02 |
| TIGD7 | 7.419124 | 9.18E-03 |
| ATG2B | 7.402605 | 2.77E-02 |
| CTDSPL | 7.382257 | 2.94E-03 |
| GJB2 | 7.322946 | 1.81E-02 |
| BEX4 | 7.307879 | 3.91E-03 |
| PEX16 | 7.282217 | 1.16E-04 |
| KCNH7 | 7.274996 | 1.03E-04 |
| ZNF805 | 7.241055 | 1.29E-02 |
| PIP5K1A | 7.214191 | 3.36E-03 |
| LOC105373637 | 7.2104 | 1.79E-03 |
| SENP1 | 7.137934 | 1.64E-02 |
| GAB1 | 7.124587 | 2.77E-02 |
| CDC27 | 7.104342 | 2.26E-04 |
| TTL | 7.102032 | 1.82E-02 |
| TOB2 | 7.094926 | 1.15E-02 |
| SNUPN | 7.088343 | 4.68E-02 |
| MGST1 | 7.085293 | 7.23E-04 |
| SPRTN | 7.054118 | 3.45E-02 |
| ZBED5 | 7.036671 | 3.20E-02 |
| CCDC149 | 6.991255 | 9.35E-03 |
| ZNF331 | 6.979646 | 4.91E-02 |
| CCDC43 | 6.976638 | 1.22E-02 |
| DRC7 | 6.907025 | 3.92E-03 |
| BLCAP | 6.869575 | 1.16E-02 |
| GCC1 | 6.830606 | 5.19E-05 |
| PCGF1 | 6.810303 | 7.56E-03 |
| TRMT10B | 6.798667 | 9.99E-03 |
| SHLD1 | 6.782135 | 1.74E-02 |
| MRPS5 | 6.743088 | 3.14E-02 |
| NIP7 | 6.736474 | 4.52E-02 |
| KIFBP | 6.735859 | 7.38E-05 |
| C14orf28 | 6.716487 | 2.72E-02 |
| TBC1D25 | 6.645957 | 2.67E-02 |
| MFSD8 | 6.602379 | 4.72E-02 |
| FAM122C | 6.600997 | 3.71E-02 |
| ARHGAP12 | 6.59402 | 5.08E-04 |
| SMIM3 | 6.53256 | 3.60E-04 |
| BCDIN3D | 6.532105 | 1.83E-02 |
| LOC112268043 | 6.531972 | 2.21E-02 |
| LEO1 | 6.517414 | 3.47E-02 |
| IL15RA | 6.475149 | 3.44E-02 |
| WDR5B | 6.466662 | 2.12E-02 |
| HOMER1 | 6.445285 | 2.47E-02 |
| ZNF699 | 6.438423 | 9.37E-03 |
| SLC41A2 | 6.42763 | 2.72E-02 |
| TAZ | 6.3791 | 6.33E-03 |
| TPCN2 | 6.292284 | 3.71E-02 |
| LOC105379087 | 6.291248 | 4.36E-02 |
| KDM7A-DT | 6.277989 | 3.47E-02 |
| ZNF737 | 6.273836 | 4.59E-02 |
| EPOR | 6.232662 | 2.46E-02 |
| SAP30L | 6.218299 | 7.56E-03 |
| MADCAM1 | 6.169486 | 2.15E-02 |
| SMPDL3B | 6.143386 | 4.99E-02 |
| LOC105378041 | 6.133138 | 5.64E-03 |
| EYS | 6.127913 | 4.01E-02 |
| RPL36A | 6.12788 | 2.75E-02 |
| C5orf51 | 6.098095 | 6.33E-03 |
| ACOX3 | 6.073169 | 1.99E-02 |
| CYB561A3 | 6.046011 | 4.99E-02 |
| KCTD13 | 6.014351 | 3.71E-02 |
| ZNF160 | 5.95619 | 1.42E-02 |
| SLC35A4 | 5.929573 | 6.40E-03 |
| VAMP1 | 5.82438 | 3.24E-02 |
| LOC339874 | 5.820421 | 3.47E-02 |
| LOC107985481 | 5.781077 | 3.71E-02 |
| ATP8B4 | 5.772533 | 3.20E-02 |
| LOC105379173 | 5.77048 | 4.42E-02 |
| NME7 | 5.675555 | 4.72E-02 |
| RPRD2 | 5.543906 | 2.91E-02 |
| LOC105370547 | 5.517336 | 4.71E-03 |
| LACC1 | 5.485197 | 1.36E-02 |
| THBS3 | 5.440757 | 4.91E-02 |
| CST3 | 5.352477 | 1.73E-04 |
| STK35 | 5.306464 | 1.29E-02 |
| FAF2 | 5.244063 | 7.13E-04 |
| CAMKK1 | 5.177845 | 7.31E-03 |
| ZNF687-AS1 | 5.015661 | 4.69E-02 |
| BTBD19 | 4.658434 | 4.72E-02 |
| HS2ST1 | 4.582038 | 1.34E-02 |
| LOC105374746 | 4.445697 | 6.69E-04 |
| MAP4K2 | 4.093854 | 2.02E-02 |
| LOC107987462 | -4.38428 | 3.77E-02 |
| SPRR2A | -4.80566 | 2.97E-02 |
| ATP1B1 | -4.88942 | 6.01E-03 |
| GPR42 | -4.8955 | 1.36E-02 |
| GALNT2 | -5.21218 | 1.22E-02 |
| PRMT3 | -5.62733 | 1.41E-02 |
| TMPRSS11B | -5.93738 | 2.46E-02 |
| PTK6 | -6.40845 | 2.75E-02 |
| LINC00954 | -6.44091 | 2.51E-02 |
| STAG3L4 | -6.58097 | 3.92E-03 |
| FOXC1 | -6.67131 | 3.24E-02 |
| CLCA4 | -6.69537 | 4.91E-02 |
| CBX8 | -6.74033 | 4.01E-02 |
| LOC101928343 | -6.92852 | 6.98E-03 |
| LOC644936 | -6.93808 | 1.61E-02 |
| LINC02019 | -7.28283 | 5.72E-05 |
| ITFG2 | -7.39464 | 3.17E-02 |
| OTOAP1 | -7.48016 | 6.65E-03 |
| KRT4 | -7.60107 | 3.96E-03 |
| LOC102725180 | -7.62226 | 2.82E-04 |
| LOC105370964 | -7.76859 | 1.25E-02 |
| KRT17 | -7.98127 | 4.94E-06 |
| CR2 | -8.04923 | 4.65E-03 |
| ZNF117 | -8.293 | 1.65E-02 |
| IL36RN | -8.39541 | 1.80E-03 |
| CBY1 | -8.48658 | 2.03E-03 |
| PRSS27 | -8.61107 | 1.09E-02 |
| ITPRIPL2 | -8.61353 | 5.80E-03 |
| NR1D2 | -8.62753 | 6.87E-04 |
| HOPX | -8.79312 | 7.04E-06 |
| KLK7 | -8.96261 | 7.99E-03 |
| ATP1A1-AS1 | -8.98374 | 8.89E-03 |
| LOC100128494 | -9.01232 | 1.01E-02 |
| NDRG2 | -9.22808 | 7.12E-03 |
| LOC107986469 | -9.24862 | 1.62E-03 |
| KLK10 | -9.5961 | 1.67E-09 |
| OTOA | -9.80419 | 7.23E-04 |
| WFDC21P | -9.89633 | 6.72E-04 |
| LOC102723729 | -10.1725 | 3.51E-05 |
| TSPAN17 | -10.4054 | 3.79E-04 |
| CDKN2A | -10.515 | 1.58E-03 |
| STXBP5-AS1 | -10.5452 | 1.57E-03 |
| STAP2 | -10.6321 | 1.86E-03 |
| IDO2 | -10.8817 | 1.11E-03 |
| WFDC2 | -10.8891 | 9.65E-04 |
| ALDH1A3 | -10.9127 | 5.14E-05 |
| LRRC75B | -11.05 | 1.15E-03 |
| MAB21L4 | -11.0668 | 4.86E-08 |
| LOC105374902 | -11.3285 | 6.89E-04 |
| SLC12A8 | -11.3834 | 8.36E-05 |
| FGD6 | -11.3928 | 6.18E-04 |
| LOC105372803 | -11.4491 | 3.81E-10 |
| VSIG10L | -11.5982 | 6.31E-04 |
| PNMA1 | -11.5985 | 4.30E-04 |
| GLO1 | -11.7579 | 5.49E-04 |
| LIMA1 | -12.0055 | 3.94E-06 |
| TMPPE | -12.0596 | 2.14E-04 |
| UCKL1-AS1 | -12.1325 | 7.06E-14 |
| CYP51A1 | -12.1899 | 2.95E-04 |
| TRUB2 | -12.2057 | 2.06E-04 |
| KRT15 | -12.4079 | 1.47E-04 |
| KRT18 | -12.6063 | 1.57E-06 |
| SLFN12 | -12.6234 | 1.24E-04 |
| CD300LD | -12.9662 | 7.06E-14 |
| MYO10 | -13.031 | 4.95E-07 |
| SPRR2F | -13.2769 | 7.88E-08 |
| TRMT12 | -13.3272 | 4.68E-05 |
| SPATS2L | -13.601 | 5.72E-05 |
| BCO2 | -13.6085 | 4.45E-05 |
| ATL1 | -13.7525 | 4.01E-05 |
| PGM3 | -14.1606 | 9.42E-06 |
| SERPINB4 | -14.2234 | 4.95E-07 |
| SPRR2E | -15.9505 | 7.15E-27 |

**Supplementary Table 3:** Genes clustered into the weighted gene correlation network analysis (WGCNA) modules

| Module | Gene |
| --- | --- |
| bisque4 | ANKRD54, APC2, ARFGEF2, C3orf20, C6orf223, COQ9, DLEU1, DNLZ, DYRK3, ECI1, ERMARD, FAHD1, FBL, FBXO22, FBXO5, GATAD1, GPR143, GRAMD4, HCFC1, HEATR3, HSPA4L, HTR7P1, HYAL2, IRF5, KIF2C, LOC100996724, LOC102723324, LOC105376933, LOC105379437, LOC107984436, LOC107985246, LOC112268051, LOC729609, LPAR1, MAK16, MBNL1-AS1, MCM6, MFSD13B, MISP3, MPHOSPH10, MSANTD4, MYC, NHEJ1, NUP62, PIGM, PLA2G6, PNO1, PTTG2, RBM4B, SEMA7A, SLC52A2, SMAD7, SNORD36A, STT3A-AS1, TCERG1, THTPA, TIGD4, TIPIN, TMEM169, TNFAIP1, UGGT2, USP24, USP32P2, WDR77, ZDHHC4, ZNF142, ZNF320, ZNF584, ZNF815P |
| black | ABHD13, ABHD18, ABHD5, ABRACL, ABRAXAS1, ACBD3, ACSL4, ADD1, ADI1, AGO4, AHCTF1, AHCTF1P1, AIF1, AMFR, ANAPC13, ANKIB1, ANKRD42, ANP32B, ANXA11, ANXA3, AP3B1, AP3S1, APOL6, ARHGAP45, ARHGEF11, ARL3, ARL6IP4, ARPC4, ARPC5, ASH2L, ATAD1, ATF4, ATG4B, ATOX1, ATP5F1E, ATP5IF1, ATP5MC2, ATP5ME, ATP5MF, ATP5MG, ATP5MPL, ATP5PB, ATP7A, ATRX, BAG1, BBIP1, BICD2, BICRAL, BLOC1S2, BLZF1, BRCA1, BTBD10, BUD31, C11orf54, C19orf53, C2orf76, C4orf3, C4orf48, C5AR2, C6orf89, CAB39, CAB39L, CABP7, CALM1, CALM2, CALM3, CAMTA1, CARS2, CASC4, CASTOR2, CBFA2T3, CBFB, CBWD1, CBX1, CCDC13, CCDC146, CCDC170, CCDC71L, CCDC9, CD37, CD59, CDC40, CDK11A, CDKN2D, CEMIP2, CEP19, CEP57, CEP63, CFAP45, CFDP1, CHCHD7, CHD8, CHFR, CIAO1, CIAO2A, CINP, CIR1, CLEC12B, CLEC2B, CLMP, CLN8, CMTM2, CNBP, CNOT7, COMMD2, COMMD6, COP1, COPS5, COX14, COX17, COX5A, COX5B, COX6A1, COX6B1, COX8A, CPEB2, CPSF7, CREB5, CSNK1G3, CSTA, CTCF, CTDNEP1, CUL4B, CUX1, CXorf38, CYB561D2, CYB5B, CYB5R1, CYSLTR1, CYTOR, DAD1, DAPK1, DAPP1, DBI, DCAF7, DEF6, DEK, DENND10, DENND10P1, DIP2B, DNAJA2, DNAJB14, DNAJB6, DPH3, DPP8, DRC1, DTX3L, DUSP22, DYNLRB1, DYNLT1, EAF2, EEF1B2, EEPD1, EGLN1, EIF2S2, EIF4EBP1, EIF4EBP2, ELMOD2, ELOB, EMBP1, EMSY, ENY2, ERF, ESD, EVI2A, EVI2B, EXOSC3, FAM133B, FAM136A, FAM160B1, FAM200B, FAM204A, FAM49A, FAM76B, FAR1, FAS, FAU, FBXL3, FBXL4, FCGR1B, FIG4, FIS1, FLI1, FOCAD, FOSB, FOXJ2, FOXJ3, FRAT2, FUT4, GABARAP, GALM, GDAP2, GIMAP4, GLRX, GLRX3, GLYR1, GMFG, GNAQ, GNG5, GOLT1B, GPN3, GPR141, GPR27, GPS2, GPSM3, GRIPAP1, GRK3, GSN, GSR, GSTK1, GSTP1, H2AC6, H2AZ1, H2BC4, H3-3A, H4C8, HADHB, HLA-B, HLX, HMCES, HMGB1, HMGN2, HMGN4, HNRNPA1, HNRNPDL, HRH2, HSBP1, HSP90B2P, HSPB11, HSPD1, HTATIP2, IFI6, IFIT5, IFITM2, IGFLR1, IGSF6, IL17RA, INTS8, IRF1, ISCU, ITM2B, ITPR2, JPT1, KCTD12, KIAA1143, KIAA2013, KIDINS220, KIF2A, KPNA4, KPNB1, LAGE3, LAMTOR2, LAMTOR4, LAMTOR5, LARP7, LASP1, LEPROT, LEPROTL1, LIN7C, LINC00623, LINC00910, LINC01138, LINC01506, LINC02067, LINC02158, LIX1L, LLPH, LOC100506023, LOC100507507, LOC101928595, LOC102723373, LOC102723878, LOC105372421, LOC105372499, LOC105373098, LOC105373582, LOC105374102, LOC105374121, LOC105374985, LOC105375713, LOC105376244, LOC105376287, LOC105376512, LOC105376786, LOC105377067, LOC105378819, LOC107984747, LOC107986158, LOC107987225, LOC112268267, LOC643072, LPIN2, LSM3, LST1, LXN, LY96, LYPLA1, LYRM1, LYST, LYZ, MACROH2A1, MALSU1, MAN1A2, MAP2K4, MAPK1, MBD2, MCTS1, MED10, MED11, MED13L, MED25, METTL14, MICOS10, MIR142, MIR22HG, MMP25, MPC2, MRPL50, MRPL53, MRPS31, MRPS36, MS4A6A, MSRA, MTHFD2, MTMR1, MYLIP, NAA38, NACA4P, NAT9, NCF2, NDRG3, NDUFA1, NDUFA12, NDUFA2, NDUFA4, NDUFA5, NDUFA7, NDUFB1, NDUFB3, NDUFB6, NDUFV3, NEDD8, NEK7, NFRKB, NFYA, NLRC5, NMNAT1, NOP10, NOTCH1, NSD3, NSMCE1-DT, NTMT1, NUDT16, NUTF2, OARD1, OGFR, ORC4, ORMDL2, OSER1, OST4, OXR1, P2RY13, PABPC1, PAIP2, PARP14, PCMT1, PDCD4-AS1, PDPK1, PDPR, PELI2, PGLS, PGM2, PGM5, PHF21A, PHKB, PIGX, PIK3CD, PIN4, PLEKHA3, PLPBP, PNPT1, POLR2I, POLR2J, PPM1A, PPM1F, PPM1M, PPP2R5C, PQBP1, PRDX5, PRPF18, PRPF4B, PRR34-AS1, PRRG4, PSMA5, PSMA7, PSMB3, PSMB7, PSMB8, PSMD10, PSMD6, PSME1, PSME2, PSME4, PSTPIP1, PTBP2, PTENP1, PTOV1-AS2, PTPRN2, PXN, PYCARD, RAB33B, RAB5IF, RABAC1, RABGAP1, RABIF, RALBP1, RALY, RANBP3, RARA, RARS2, RASGRP2, RASSF3, RBM17, RBM26, RBP7, RCBTB2, RCOR1, RELCH, REM2, RFX1, RGS14, RHOT1, RNF114, RNF141, RNF145, RNF181, RNF20, RNF213, RNF38, RNF44, RNFT1, RNPEP, ROPN1L, RP2, RPA3, RPL13, RPL21, RPL23A, RPL24, RPL26, RPL27A, RPL29, RPL34, RPL35A, RPL36AL, RPL37, RPL38, RPL7A, RPL9, RPS11, RPS12, RPS15, RPS19, RPS19BP1, RPS24, RPS26, RPS27A, RPS3, RPS7, RPS9, RRP12, RSRP1, RTN4, RUFY2, S100A11, S100A12, S100A4, S100A6, S100A8, S100A9, S1PR4, SAP18, SAR1A, SBF2, SCAF8, SCARNA10, SCFD1, SCNM1, SDHB, SDHD, SEC11A, SEC22C, SEMA4A, SEMA4D, SENP5, SENP6, SERTAD3, SF3A1, SF3B5, SF3B6, SFPQ, SFSWAP, SGPL1, SH3GLB1, SIAH2, SLC25A5, SLC31A1, SLC35A1, SLC44A2, SLC45A4, SLC48A1, SLC8A1-AS1, SLCO3A1, SMARCA5, SMIM14, SMIM25, SMIM26, SMIM27, SMIM7, SNAPC5, SNF8, SNORA105A, SNORD141B, SNORD14D, SNORD14E, SNRK, SNRNP25, SNRNP27, SNRNP70, SNRPE, SNTB1, SNX2, SNX3, SP140, SP140L, SPCS1, SPIDR, SPTLC2, SREK1IP1, SRP14, SRSF9, SS18L2, ST3GAL6, STAG1, STAMBP, STAT2, STEAP4, STX3, STXBP5, SULT1B1, SUPT16H, SVBP, SWAP70, SYF2, TACC1, TAF10, TAF12, TARDBP, TBC1D1, TBCA, TBL1X, TFEB, TGFBR2, THAP1, THOC2, TIAL1, TIMM8B, TIPRL, TMA7, TMCO1, TMCO6, TMEM107, TMEM131L, TMEM144, TMEM14B, TMEM159, TMEM199, TMEM219, TMEM30A, TMEM50A, TMEM70, TMSB4X, TOPORS, TOR1AIP2, TPRG1L, TPST2, TRADD, TRAF3IP2-AS1, TRAPPC1, TRAPPC6B, TRDMT1, TRIOBP, TRMT10A, TRMT112, TSPAN2, TTC1, TTC17, TTC9C, TUBA1C, TUBGCP4, TUSC2, TXN, TXNDC17, TXNDC9, TXNIP, TXNL4B, TYROBP, U2AF1L5, UBA3, UBA52, UBC, UBE2D1, UBE2K, UBE2L3, UBE2R2, UBR3, UNC93B1, UPF2, UQCR10, UQCR11, UQCRC1, USP25, USP47, UTP6, VAMP3, VAMP4, VAMP5, VAPA, VNN2, VPS29, VPS36, VPS41, WBP4, WDR82, WDR83OS, WIPF1, WIPI1, XAF1, XPNPEP3, YIPF1, YPEL5, YY1, ZBTB18, ZBTB34, ZBTB8OS, ZCCHC9, ZDHHC17, ZFAS1, ZFP36L2, ZFP91, ZMAT2, ZMAT5, ZNF200, ZNF271P, ZNF284, ZNF516, ZNF641, ZNF700, ZNF770, ZNF776, ZNF90, ZNHIT1 |
| blue | ABALON, ABCC11, ABCD3, ABHD16A, ACAA1, ACOT12, ACP7, ACRBP, ACSL1, ACSL3, ACTR3, ADAM17, ADAM20, ADAM9, ADAMTS13, ADAMTS4, ADARB1, ADAT1, ADCY9, ADGRF3, ADORA2A-AS1, ADTRP, AGBL3, AGPAT3, AGRN, AICDA, AK3, AKAP5, ALPL, AMPD3, AMY1B, ANKFN1, ANKH, ANOS1, ANPEP, ANTKMT, ANTXR2, AP3S2, APLP1, APLP2, ARF1, ARF4, ARFGAP3, ARHGAP15-AS1, ARHGAP26, ARHGEF35, ARMC12, ARRDC2, ARRDC3, ASAP3, ASB11, ASCC2, ASPH, ASTL, ASXL3, ATF1, ATP6AP1, ATP8B2, ATP9B, AZIN1-AS1, B4GALT1, BAIAP2L1, BAIAP3, BASP1, BCAT1, BCKDK, BCL2L13, BCL3, BCL9, BEST2, BICD1, BID, BNIPL, BOLA3, C12orf54, C17orf58, C1orf61, C1orf74, C1QTNF7, C2CD4B, C3AR1, C6orf99, CACHD1, CACNA2D1, CAHM, CALML4, CAND1.11, CANX, CAPN7, CASP1P2, CATIP-AS1, CATIP-AS2, CBLIF, CCBE1, CCDC24, CCDC36, CCDC82, CCDC83, CCL18, CCM2L, CCNB1, CCNE2, CD177, CDC37, CDC42SE1, CDC7, CDH23, CEACAM7, CELF1, CEP83, CEP83-DT, CERT1, CFAP44, CFL1P1, CFLAR-AS1, CHAC1, CHRNE, CKAP2L, CLDN17, CLRN1, COL1A1, COL7A1, COMMD4P2, CORIN, CROCC, CRYAB, CRYBA4, CSKMT, CT75, CTAGE10P, CTB-178M22.2, CTDSPL, CTNNA1, CTNNBL1, CTSB, CUBN, CXCL5, CXorf40B, CYB5R2, CYFIP1, CYGB, CYLD, DAND5, DBNL, DCTN4, DDAH2, DDX27, DDX50, DEFB4B, DENND1A, DENND4C, DFFB, DHCR7, DHDH, DHX16, DHX34, DLEU2L, DLG4, DLGAP1, DLGAP1-AS1, DLGAP1-AS2, DMXL2, DNAJB5, DNAJC4, DND1, DPCD, DPH1, DUSP5P1, DXO, DYSF, EBI3, ECE1, EFCAB7, EID3, EIF2S3B, EIF4E, EIF4H, EIF6, ELK1, ELL3, ELOVL6, EML2, ENTPD7, ERV3-1-ZNF117, FABP12, FAHD2B, FAHD2CP, FAM120B, FAM122B, FAM153CP, FAM160A1, FAM180A, FAM66C, FAM71D, FBXO42, FCAR, FCN2, FERMT3, FKBP1A-SDCBP2, FKBP6, FLG, FLOT1, FNDC3A, FNTA, FOSL1, FPR1, FPR2, FRMD6-AS1, GAB2, GABRR1, GBAP1, GCH1, GET4, GGCT, GINS4, GJB2, GK3P, GLDN, GLTP, GNA15, GOLGA7, GPAT4, GPATCH3, GPR160, GPR21, GPR35, GPRC5D, GREB1L, GRIK1, GRK5-IT1, GRK6P1, GSTM3, H3C1, HCG17, HCG20, HCG9, HCK, HDGF, HEXA, HIF1AN, HIVEP1, HMOX2, HOMER3-AS1, HOXA3, HPSE, HRH1, HSD3B1, HSD3B7, HSF4, HSP90AB4P, HSPA1L, HSPB9, HTR2B, HTRA3, IAH1, IBA57-DT, IGSF3, IL10RB, IL18R1, IL19, IL31RA, ILK, INCA1, INE1, INF2, IPO5P1, IQCD, IRAK1, IRS2, ITGA7, IVL, IZUMO4, JAKMIP2, KCTD20, KDF1, KDM4C, KIAA1755, KIF5A, KL, KLHL29, KPNA5, KREMEN1, KRT13, KRT6C, LACC1, LACTB2, LAT, LATS2, LBX2-AS1, LIF-AS1, LIMK1, LIMK2, LIMS1-AS1, LINC00051, LINC00310, LINC00365, LINC00449, LINC00528, LINC00540, LINC00545, LINC00676, LINC00894, LINC00908, LINC01004, LINC01061, LINC01128, LINC01147, LINC01187, LINC01224, LINC01339, LINC01362, LINC01465, LINC01521, LINC02009, LINC02193, LINC02259, LINC02345, LINC02348, LINC02354, LINC02470, LINC02597, LINC02733, LMLN, LMO7, LOC100128966, LOC100129617, LOC100129878, LOC100130027, LOC100130691, LOC100130987, LOC100131635, LOC100505501, LOC100506679, LOC100996442, LOC101927044, LOC101927045, LOC101927393, LOC101927897, LOC101928045, LOC101928055, LOC101928077, LOC101928344, LOC101928386, LOC101928445, LOC101928841, LOC101929004, LOC101929141, LOC101929290, LOC101929305, LOC101929524, LOC101929894, LOC102723413, LOC102723530, LOC102723604, LOC102723701, LOC102723914, LOC102724351, LOC102724467, LOC102724545, LOC102724584, LOC102725035, LOC102725112, LOC105369312, LOC105369403, LOC105369656, LOC105369699, LOC105369779, LOC105369949, LOC105370034, LOC105370087, LOC105370108, LOC105370525, LOC105370677, LOC105370816, LOC105370829, LOC105370877, LOC105371044, LOC105371159, LOC105371251, LOC105371403, LOC105371729, LOC105371814, LOC105371819, LOC105371870, LOC105371965, LOC105372211, LOC105372234, LOC105372380, LOC105372448, LOC105372498, LOC105372501, LOC105372705, LOC105372825, LOC105372945, LOC105372988, LOC105373023, LOC105373172, LOC105373211, LOC105373428, LOC105373552, LOC105374234, LOC105374383, LOC105374418, LOC105374464, LOC105374724, LOC105374927, LOC105374981, LOC105375068, LOC105375211, LOC105375231, LOC105375409, LOC105375508, LOC105375566, LOC105375633, LOC105375769, LOC105375857, LOC105375914, LOC105375936, LOC105376025, LOC105376196, LOC105376224, LOC105376479, LOC105376505, LOC105376866, LOC105376956, LOC105377160, LOC105377291, LOC105377549, LOC105377621, LOC105378009, LOC105378443, LOC105378629, LOC105378776, LOC105378843, LOC105378925, LOC105378939, LOC105378943, LOC105378948, LOC105378988, LOC105379087, LOC105379321, LOC105379725, LOC107983996, LOC107984063, LOC107984128, LOC107984129, LOC107984184, LOC107984220, LOC107984346, LOC107984516, LOC107984558, LOC107984635, LOC107984647, LOC107984652, LOC107984698, LOC107984709, LOC107985227, LOC107985308, LOC107985314, LOC107985334, LOC107985485, LOC107985505, LOC107985785, LOC107985940, LOC107986004, LOC107986162, LOC107986364, LOC107986431, LOC107986465, LOC107986776, LOC107986798, LOC107986812, LOC107986982, LOC107987272, LOC107987311, LOC107987463, LOC112267866, LOC112268089, LOC112268145, LOC112268166, LOC112268269, LOC112268419, LOC112268447, LOC148709, LOC283922, LOC285847, LOC286059, LOC339874, LOC644656, LRRC17, LRRC23, LRRN3, LTBP2, LTO1, LYPD3, LYPLA2P2, LZTS1, LZTS1-AS1, MAGT1, MAMLD1, MAP1B, MAP3K11, MAP3K13, MAP3K19, MAP4K4, MAPK13, MAPK1IP1L, MAPRE1, MARCKS, MAS1, MBNL2, MCCC2, MCEMP1, MCPH1-AS1, MDGA2, MED20, MFSD11, MFSD6, MIR12122, MIR194-2HG, MIR3161, MIR3945HG, MIR4313, MIR4526, MIR4748, MIR5700, MIR6075, MIR6732, MIR6855, MIR938, MIR9902-1, MIR9902-2, MIS18A, MITF, MLLT6, MLPH, MMACHC, MMP14, MOB3B, MORN3, MPP1, MR1, MRNIP, MROH1, MRPL4, MSANTD3, MST1, MSTO2P, MTFP1, MTHFD2L, MTMR2, MTMR6, MTRNR2L2, MUC21, MUS81, MYH9, MYL6B, MYO1B, MYOM2, N4BP3, NAF1, NBEAL1, NCOR2, NCR3LG1, NEB, NEBL, NECAP1, NEK8, NEURL3, NFASC, NINL, NKAPL, NLRP3, NLRP7, NME9, NOS2P3, NPIPA5, NPIPB4, NPPA, NPPA-AS1, NR1H2, NRROS, NSUN6, NTSR1, NUCB1, NUMB, OBI1, OPLAH, OR10P1, OSCAR, OXSM, PABPC3, PABPN1L, PACSIN2, PARD6G-AS1, PDXP, PDYN-AS1, PEBP1, PEG13, PEX11G, PFN1, PGK1, PHEX, PHF1, PHF20, PIGA, PILRB, PLB1, PLD6, PLPP6, PLVAP, PMS2P3, PNPLA1, PNPLA6, POPDC2, POTEF, POTEJ, POU6F1, PPARG, PPIAL4C, PPM1H, PPP2R3B, PPP4R1-AS1, PPP6R2, PPRC1, PREX1, PRKAG1, PRNP, PRRT4, PSG9, PTGES, PTPN7, PTPRD, PUS7, PUSL1, QPCTL, QSOX1, RAB3C, RAB6C, RAB6D, RABEP1, RAD54L, RASA3, RASA4, RASA4CP, RASD1, RASL11A, RAVER2, RB1, RBM23, RD3L, REPIN1, RETREG1, RFPL1S, RGL3, RILP, RIT1, RMDN3, RNF126, RNF144B, RNF165, RNF227, RNU6-9, RNVU1-3, RPA4, RPTN, RRAD, RTCA-AS1, RTF2, RTRAF, RXRA, RYBP, SAMSN1-AS1, SBSN, SCARNA4, SCG3, SEC23B, SERINC3, SERPINA1, SERPINA2, SERPINB9, SERTAD1, SESN2, SETD5, SGPP2, SH2B2, SH2D2A, SH2D6, SH3TC2, SHISA4, SLA, SLC10A1, SLC15A3, SLC16A3, SLC22A18AS, SLC25A17, SLC26A5, SLC35B2, SLC35E4, SLC39A7, SLC43A2, SLC7A5, SLC9A4, SLFNL1-AS1, SLX1B, SMIM13, SMPD1, SNORA101B, SNORA10B, SNORA118, SNORA14A, SNORA19, SNORA7B, SNORD127, SNORD143, SNORD164, SNORD168, SNORD2, SNORD7, SNORD71, SNORD74, SNORD98, SNU13, SNX20, SNX27, SNX29P2, SNX32, SNX9, SPACA6, SPAG7, SPATA6L, SPDYE6, SPECC1L-ADORA2A, SPI1, SPIN4, SPINK9, SPTBN5, SQLE, SQOR, SRR, SSH1, ST13, ST20-AS1, ST3GAL1, ST6GALNAC3, ST7L, STAT4, STK26, STK40, STRIP2, STXBP2, STXBP5L, SULT2A1, SUPT5H, SYNE4, SYNPO2L, TAPBP, TAS1R1, TBC1D22A-AS1, TBC1D3E, TBC1D3J, TBC1D3K, TBK1, TBX6, TCAF2, TDRD9, TFDP1, TFRC, TGIF2, THAP7, TICAM1, TIPARP, TKFC, TKTL1, TLN1, TLNRD1, TM9SF4, TMED3, TMEM117, TMEM120B, TMEM132A, TMEM254-AS1, TNC, TNFAIP2, TNFRSF14-AS1, TNFRSF21, TNFSF13, TNFSF8, TOMM40L, TOR1B, TPK1, TRAF3IP3, TRAPPC10, TREM2, TRIB3, TRIM26, TRIO, TRMT44, TRPS1, TSG101, TSHR, TSPYL2, TSSK4, TTC28, TTLL3, TTPAL, TUBA4B, TUBB, TUBB2B, TWIST2, TWISTNB, TYRO3, UBA6, UBAP2L, UBL3, UBR1, UBTD2, UGT2B11, UPK3B, UPP2, UQCC1, URGCP, USF3, USP28, USP32P3, VAC14-AS1, VASN, VAV1, VAV3, VAV3-AS1, VEGFB, VIL1, VMAC, VPS13D, VPS33A, WASHC2A, WDR93, XIAP, YTHDF3, ZBTB1, ZBTB17, ZBTB25, ZBTB37, ZCWPW1, ZEB1-AS1, ZFAND1, ZFP62, ZMYND11, ZNF107, ZNF131, ZNF207, ZNF232, ZNF252P-AS1, ZNF254, ZNF273, ZNF281, ZNF34, ZNF385B, ZNF410, ZNF429, ZNF441, ZNF517, ZNF556, ZNF596, ZNF674, ZNF681, ZNF705A, ZNF76, ZNF850, ZSCAN12P1 |
| brown | ABCC2, ABCF3, ABCG1, ABI3BP, ACBD7, ACIN1, ACSF3, ACSL3-AS1, ACTG1P20, ACYP2, ADAM28, ADGRE2, ADGRG1, ADGRV1, ADIPOR2, ADM, AGAP12P, AGK, AGPAT1, AKAP10, AKIRIN1, AKR1A1, ALAD, ALDH3A1, AMPD2, ANG, ANGPTL1, ANKRD10, ANKRD11, ANKRD17, ANKRD20A11P, ANKRD27, ANKRD36, ANKRD36B, ANKRD39, ANO7, AP4B1-AS1, APEH, APOC1, APP, APTX, ARFRP1, ARG1, ARHGAP40, ARHGEF10, ARHGEF18, ARID5A, ARNTL2, ARNTL2-AS1, ARRDC1-AS1, ASB14, ATAD2B, ATAD3B, ATL1, ATOH8, ATP1B1, ATPSCKMT, ATRN, AUP1, AZU1, B3GALT5, B3GNT4, B3GNT7, BBS1, BCL11A, BCL7B, BMP8A, BNC1, BORCS7-ASMT, BRICD5, BRIP1, BRPF3, BSDC1, BTBD9-AS1, C10orf126, C11orf21, C19orf12, C19orf54, C1QA, C20orf197, C22orf39, C22orf46, C4BPA, C5orf15, C5orf46, C8orf44-SGK3, C8orf76, C9orf139, CAP2, CAPN5, CARS1, CASC3, CASP10, CASP17P, CASP5, CASTOR3, CBX4, CC2D1B, CCDC15, CCDC85A, CCEPR, CCL2, CCL5, CCL8, CCNA2, CCNJ, CD300LD, CDC42EP2, CDH26, CDH3, CDK11B, CDK5RAP3, CDK7, CDS1, CELA2A, CELF2-AS1, CEP44, CEPT1, CERS6, CFAP410, CFD, CHMP1A, CIB1, CLCA4, CLCN5, CLDN1, CLDN11, CLDN23, CLECL1, CLK2, CLK2P1, CLOCK, CLPP, CLSTN3, CMYA5, CNTN2, COG7, COMT, COPRS, COQ4, CPSF4L, CPXM2, CREG1, CRTC3, CRYL1, CRYM, CSPP1, CTB-3M24.3, CTBS, CTRC, CUL2, CUL4A, CWC27, CYCSP52, CYP17A1, CYP17A1-AS1, CYP24A1, DAAM1, DAB1, DALRD3, DARS2, DBT, DCUN1D2, DDTL, DDX3X, DDX6, DERL1, DESI1, DHX9, DISC1, DISC1-IT1, DLG5, DLGAP4, DLL4, DMAC2L, DNAJB2, DNAJB4, DNAJB9, DNAJC5B, DNAL1, DOCK7, DPAGT1, DSP, DTX2P1-UPK3BP1-PMS2P11, DUOX1, DYNC2H1, EBNA1BP2, ECHDC2, EDC4, EDRF1-AS1, EFCAB5, EFHB, EGFL7, EHBP1L1, EIF1AX, EIF3D, EIF5, EMG1, EMP1, EMX2OS, ENDOV, ENTPD1-AS1, ENTPD4, EPHA1-AS1, EPS15L1, ERAL1, ERP44, ESRP2, ETNK1, EXTL2, EXTL3, FAAP100, FAM122A, FAM133CP, FAM13B, FAM156A, FAM156B, FAM157A, FAM160A2, FAM168A, FAM174B, FAM215B, FAM237B, FBLN7, FBN2, FDPSP2, FFAR3, FHL2, FKBP11, FKBP15, FKBP9P1, FMNL1, FMNL3, FNDC9, FOXP2, FOXRED2, FUBP1, FUT2, GABBR1, GABRE, GAS5, GATD1, GBP6, GCC2, GDF15, GGCX, GIGYF1, GLMN, GLRX5, GMDS-DT, GNE, GOLGA4, GOLM1, GON4L, GPR18, GPR34, GPR42, GRPEL2, GRWD1, GTF2IP23, GTPBP2, GTPBP6, GUCA1B, HACD2, HDAC3, HDGFL3, HDHD2, HECW2-AS1, HEXD, HIVEP3, HLA-DQA1, HLA-DQB1-AS1, HLA-DRB1, HLA-DRB5, HLA-DRB6, HMBOX1, HMGN1, HOOK3, HSD17B13, HYOU1, ICE1, ICMT, IFI27L2, IFITM10, IGSF5, IKBKE, IL12B, IL12RB2, IL2RA, IL6ST, IMMP1L, INHBE, INPP5E, IP6K1, IP6K2, IPCEF1, ITGAV, ITGB6, ITLN1, ITPRIPL1, JAK3, JMJD4, KARS1, KCNK1, KCNK5, KCTD5, KDM5A, KDSR, KHDC4, KIAA0586, KIAA0753, KIF15, KLHL12, KLK10, KLK11, KMT2B, KRI1, KRT17, KRT23, KRT7, LAMC2, LCOR, LDB3, LENG8, LHFPL2, LHFPL3, LIG4, LILRA3, LILRA6, LIME1, LINC00266-3, LINC00304, LINC00342, LINC00563, LINC00862, LINC00954, LINC01001, LINC01002, LINC01270, LINC01348, LINC01512, LINC01821, LINC01857, LINC02018, LINC02019, LINC02028, LINC02284, LINC02487, LINC02570, LINC02605, LINC02736, LINC02803, LIPK, LIPM, LIX1L-AS1, LMBR1L, LNPK, LOC100130283, LOC100268168, LOC100287015, LOC100288203, LOC100505728, LOC100506606, LOC100506974, LOC100996583, LOC101926944, LOC101926994, LOC101927143, LOC101927400, LOC101927727, LOC101927751, LOC101927822, LOC101927960, LOC101927963, LOC101928166, LOC101928331, LOC101928696, LOC101928786, LOC101928816, LOC101929129, LOC101929331, LOC101929528, LOC101929595, LOC101929705, LOC101929707, LOC102723406, LOC102723409, LOC102724159, LOC102724250, LOC102724808, LOC102724960, LOC102724995, LOC102725180, LOC105369344, LOC105369382, LOC105369397, LOC105369468, LOC105369953, LOC105370117, LOC105370118, LOC105370202, LOC105370401, LOC105370447, LOC105370522, LOC105370871, LOC105370984, LOC105371341, LOC105371380, LOC105371461, LOC105371581, LOC105371753, LOC105372256, LOC105372275, LOC105372814, LOC105372877, LOC105372956, LOC105372997, LOC105373142, LOC105373170, LOC105373847, LOC105374310, LOC105374768, LOC105374852, LOC105375056, LOC105375668, LOC105375768, LOC105375938, LOC105375943, LOC105376018, LOC105376217, LOC105376292, LOC105376478, LOC105376767, LOC105377275, LOC105377299, LOC105377615, LOC105377814, LOC105377924, LOC105378286, LOC105379227, LOC105379252, LOC105379337, LOC105379444, LOC105379513, LOC105379857, LOC107983971, LOC107984010, LOC107984026, LOC107984202, LOC107984214, LOC107984221, LOC107984388, LOC107984432, LOC107984741, LOC107984758, LOC107984917, LOC107985063, LOC107985193, LOC107985239, LOC107985315, LOC107985513, LOC107985915, LOC107985986, LOC107986084, LOC107986177, LOC107986361, LOC107986476, LOC107986595, LOC107987043, LOC107987118, LOC107987141, LOC107987240, LOC107987281, LOC107987433, LOC112267942, LOC112268014, LOC112268070, LOC112268100, LOC112268177, LOC112268202, LOC112268322, LOC114224, LOC145474, LOC286437, LOC389831, LOC390877, LOC441081, LOC644285, LOC646626, LOC646652, LOC652276, LOC653513, LOC728613, LOC729732, LOC729737, LOC729870, LOC729970, LOC91370, LPEQ6126, LPO, LRCH1, LRMDA, LRP2BP, LRP6, LRRC3, LRRC37A3, LRRC77P, LSM11, LSM5, LTC4S, LY75-CD302, LYPD2, LYPLA2, MAP11, MAP3K4, MAPK8IP3, MARCHF5, MARCO, MARS1, MAT2A, MCMDC2, METTL22, METTL25, MFSD14A, MICAL1, MICOS10P1, MIER2, MIGA1, MIPOL1, MIR12136, MIR186, MIR222, MIR3064, MIR4420, MIR4453HG, MIR4639, MIR4742, MIR548AC, MIR604, MIR6758, MIR6837, MLXIP, MLYCD, MOCS3, MORC3, MPV17L, MRI1, MRPL14, MRPS22, MSN, MSTO1, MT1L, MTA2, MTG1, MTG2, MTO1, MUC3A, MUC4, MUC5B, MYLK, MYO18A, MYO1C, MYO1E, MYO5B, MYOM1, MYOZ3, MZF1, NADSYN1, NAV1, NCAPG2, NCSTN, NDC80, NDUFS2, NEGR1, NEIL2, NELFB, NEPRO, NHLRC2, NINJ2-AS1, NKG7, NLGN1, NLRC4, NLRP1, NMD3, NNMT, NOA1, NOP56, NPIPB13, NPR3, NPTN, NQO1, NRBP1, NRTN, NSRP1, NSUN2, NTPCR, NTRK2, NUDT13, NUDT17, NUP85, OR13C8, OR7E5P, ORMDL3, OSER1-DT, OSMR, OTUD5, OVOL1, P2RX7, P2RY2, P3H2, PADI1, PAFAH2, PAGR1, PARD6B, PARVB, PATL2, PAXX, PCF11, PCGF3, PCNX4, PCOLCE2, PCP2, PCYT1A, PDE3B, PDE5A, PDIA3P1, PDZD11, PEX13, PHKG2, PHLDA1, PHYKPL, PIF1, PIKFYVE, PITRM1, PITX1, PLBD2, PLIN3, PLK4, PMS2P2, PMS2P5, PMS2P7, POC1B-GALNT4, POLR2J4, POMZP3, POU2F1, PPHLN1, PPIEL, PPP1R12C, PPP1R1C, PPP1R35, PPP4C, PPP4R3B, PPP6R3, PPWD1, PRDX6, PRG4, PRICKLE3, PRKAR1B, PRKD3, PROCR, PRPSAP1, PRR25, PRR7, PRSS22, PSMD6-AS2, PSME3IP1, PTAR1, PTBP1, PTPA, PTPN3, PTRH2, PUS10, PUS7L, PWP2, RAB12, RAB25, RAB2A, RAB3B, RAB42, RAB6A, RALGAPB, RAP1B, RAPGEF3, RBM28, RBM3, RBM4, RER1, RFNG, RFTN2, RIC8B, RIN3, RINL, RIPPLY3, RMDN2, RNASE1, RNASE2, RND1, RND3, RNF149, RNF222, RNF43, ROCK1P1, ROM1, RPL12, RPS18P9, RPS6KA2, RRAGB, RRN3P3, RRP9, RSKR, RSPH3, RSRC2, SAA1, SAA2, SAR1B, SBF2-AS1, SBNO1, SCARNA21, SCYL1, SDCCAG8, SDF4, SDHAF3, SDHC, SDR9C7, SDS, SEC14L1, SECTM1, SELENOM, SELENOS, SEPHS2, SEPTIN11, SEPTIN7P2, SERPINA11, SERPINB13, SESN1, SGTA, SH3BGRL2, SH3BP2, SHARPIN, SHB, SHC1, SHISAL2A, SIDT1, SINHCAF, SIPA1L2, SLC13A5, SLC15A1, SLC15A2, SLC16A10, SLC16A6, SLC1A2, SLC1A4, SLC25A22, SLC25A23, SLC25A37, SLC26A9, SLC2A13, SLC30A5, SLC39A6, SLC5A9, SLC6A4, SLC7A6, SLX4IP, SMAD2, SMARCC2, SMG1P1, SMG1P3, SMN1, SND1-IT1, SNORA103, SNORA48, SNORC, SNORD13E, SNORD14A, SNORD16, SNORD44, SNORD47, SNORD75, SNORD76, SNORD77, SNORD78, SNORD79, SNORD80, SNORD81, SNORD87, SNRNP48, SNX13, SNX17, SNX29P1, SNX30, SOCS1, SOCS5, SPACA5B, SPATA16, SPDYE16, SPDYE18, SPIRE1, SPPL3, SPRR2G, SPSB3, SPTLC1, SRP68, SRRM1, SRRM2, SRRM4, SRSF11, SRSF5, SS18L1, STAG3L4, STAU2, STK36, STK38, STK4, STX1B, SUGP2, SULF2, SULT1A2, SUMO1P3, SUV39H2, SYN1, SYT16, TAF1C, TAF9B, TAS2R38, TAS2R60, TBC1D10A, TBC1D3P1-DHX40P1, TBC1D8, TBCCD1, TBL1XR1, TBP, TBRG1, TCF20, TCN1, TFG, TFPT, TGM2, THAP5, THNSL2, TJP3, TLCD2, TMC5, TMCO3, TMCO5A, TMEM126A, TMEM129, TMEM131, TMEM165, TMEM167B, TMEM230, TMEM240, TMEM35B, TMEM39A, TMEM67, TMEM71, TMEM9B, TMPRSS2, TMUB1, TNFRSF1A, TNFSF4, TNIK, TNKS1BP1, TOE1, TP53RK, TPBG, TPM3, TRAK1, TREML5P, TRIM3, TRIM31, TRIM6, TRPA1, TRPM7, TSFM, TTBK2, TTC4, TTYH3, TUBBP5, TUFM, TULP4, TUT1, TWF1, TWSG1, TXLNB, TXNRD1, TYW5, UBE2D4, UCKL1-AS1, UHRF2, UNC119, UNC13C, UPF1, UPK3BL2, UQCRBP1, USP2, USP21, USP30, USP33, UTP14A, VPS13B-DT, VPS50, VPS8, VSIG2, VSIG4, WDFY1, WDR43, WFDC2, WHAMMP1, WHAMMP3, WIPF2, XPO1, XRCC1, YRDC, YTHDF2, ZBTB16, ZBTB20, ZBTB24, ZBTB40, ZBTB49, ZBTB8A, ZC3H11B, ZC3H13, ZCCHC24, ZDHHC18, ZDHHC6, ZFAND4, ZKSCAN8, ZMAT3, ZMIZ2, ZNF224, ZNF230, ZNF41, ZNF414, ZNF45, ZNF513, ZNF518A, ZNF564, ZNF585A, ZNF597, ZNF598, ZNF644, ZNF689, ZNF69, ZNF709, ZNF738, ZNF804A, ZNF841, ZNRF3, ZP3, ZPR1, ZRANB1, ZRANB2-AS1, ZSCAN18, ZZEF1 |
| brown4 | ABCD1, ABHD17A, ACTR3C, ADK, AGGF1, AKAP9, ANGPT1, ANKRA2, AP2A1, ARF3, ARHGEF37, ATF7, ATMIN, C14orf93, C5orf22, C9orf85, CASP8AP2, CCDC77, CCN3, CCR3, CLCN4, CLEC9A, COIL, DACH1, DDX1, DGKZ, DICER1-AS1, DOCK8-AS1, DUS2, EFHC2, EIF2AK4, EML3, ENTPD3-AS1, ERCC1, ERH, FAM111A, FAM241A, FAM43A, FUT7, G6PD, GSDMD, HFE, IL12RB1, IMPAD1, IRF3, ISL2, KAT2A, KRIT1, LCAT, LGALS3BP, LINC02705, LOC100289230, LOC101927100, LOC102724957, LOC105369219, LOC105371948, LOC105375035, LOC105375112, LOC107984561, LOC107986751, LRBA, MEGF6, MFAP3, MGRN1, MRPS10, MRPS15, NUDT16P1, ODF2L, PDHA1, PFDN4, PHB, PHKA2-AS1, PIN1, PKP4, PPP2R1A, PRDX2, PRRC2A, PSMB10, PSMG4, PTER, RDH11, REPS2, RINT1, RP9P, RPL10A, RPRD1B, S100PBP, SCAT8, SEC24B, SELL, SFN, SLC22A5, SMC1A, SOX6, TBC1D5, TEX30, TMEM147, TMEM243, TMEM41A, TRAK2, TRAPPC2, TSPAN32, TXNDC15, TXNRD2, USP53, YBEY, YPEL2, ZDHHC12, ZDHHC2, ZNF185, ZNF580, ZNF626, ZNF787 |
| cyan | ABCA1, ABCG2, ABHD14A, ABHD14B, ACSBG2, ACSL5, ADAMTS9-AS1, ADAMTSL4-AS1, ADAMTSL4-AS2, ADCY5, ADCY8, ADRA1A, AGAP10P, AGAP7P, AGPAT4, AKR1C6P, ALOX12-AS1, ANKRD37, APOBEC2, AQP4-AS1, ARHGAP10, ARHGAP22, ARMCX4, ASIC4-AS1, ATF5, BCAP29, BCAS1, BCAS3, BEST1, BEST4, C2, C5orf58, C9orf72, CACNB2, CASP12, CBARP, CCAR2, CCL3L3, CCNL1, CCSER2, CDC42-AS1, CDCP1, CELF4, CENPBD1P1, CENPO, CEP41, CHADL, CHD1, CHRM3, CHRNA10, CHST1, CIAO3, CLDN7, CNST, COMMD4P1, CORO7, CPEB4, CRNN, CSNK1D, CTAGE15, CTAGE6, CWC22, CWC25, CXCL17, DARS1, DBF4, DCAF10, DENND4A, DGAT1, DIRAS1, DNAH17, DSEL, DUSP28, E2F8, ECE1-AS1, EEF2KMT, EGF, EIF2A, EIF3I, ELF3, ENPP7, EPHA7, ERI1, FAM209A, FAM85B, FAM86B3P, FLJ22447, FLT1, FMO2, FNIP2, FOLR1, FTX, GASK1B, GCLM, GCNT2, GCNT7, GGT7, GLIS3, GNA12, GNA13, GORASP1, GOSR1, GP6, GPR137B, GPR61, GRTP1, GS1-124K5.11, GS1-279B7.1, H2BC7, HAS1, HDAC11, HGSNAT, HLA-DQA2, HLA-DQB1, HLA-DQB2, HLA-J, HMGXB4, HNRNPA3, HORMAD1, HPCA, HPN, HS3ST3A1, HSD17B7P2, HSPA13, IARS1, IER3, IFRD1, IFT74, IL18BP, IL1R2, IL1RAP, IL1RL2, IL36G, IMPA1P1, IPO11-LRRC70, IPPK, JRKL, KCNT1, KIAA0825, KIF1A, KIF25, KRT19, KRT78, KYNU, LARP6, LCE5A, LDAH, LILRB5, LINC00265, LINC00581, LINC00641, LINC01262, LINC01267, LINC01277, LINC01347, LINC01550, LINC02063, LOC100132062, LOC100132287, LOC100133091, LOC100134040, LOC100507291, LOC100652833, LOC101927243, LOC101927300, LOC101927668, LOC101928438, LOC101929709, LOC101929863, LOC101930085, LOC102723414, LOC102724907, LOC105369318, LOC105369632, LOC105369722, LOC105369771, LOC105369812, LOC105370203, LOC105370355, LOC105370965, LOC105371026, LOC105371414, LOC105371551, LOC105371777, LOC105372321, LOC105372411, LOC105372412, LOC105372511, LOC105372768, LOC105373086, LOC105373415, LOC105374152, LOC105374209, LOC105374298, LOC105374493, LOC105374925, LOC105374942, LOC105375051, LOC105375822, LOC105376026, LOC105376078, LOC105376204, LOC105376544, LOC105376577, LOC105376781, LOC105377054, LOC105377303, LOC105377406, LOC105377750, LOC105377979, LOC105378085, LOC105378104, LOC105378204, LOC105378408, LOC105378457, LOC105378504, LOC105378606, LOC105378620, LOC105378726, LOC105379003, LOC105379185, LOC105379331, LOC107105282, LOC107984088, LOC107984156, LOC107984567, LOC107984918, LOC107985080, LOC107985180, LOC107985203, LOC107985233, LOC107986016, LOC107986148, LOC107986185, LOC107986363, LOC107986532, LOC107986646, LOC107986780, LOC107987063, LOC107987145, LOC107987437, LOC107987462, LOC112267984, LOC112268061, LOC112268097, LOC112268174, LOC112268337, LOC399900, LOC400682, LOC653653, LOC728715, LOC729603, LPAR6, LRRC14B, LRRC70, LRRC74B, LYSMD3, MAGEB10, MAGI1-AS1, MCF2L2, MECP2, MED1, MED14, MEF2C, MEF2D, MEIS3P1, MGA, MIR2052HG, MIR221, MIR570HG, MIR611, MIR636, MIR6876, MIRLET7BHG, MMAA, MSI2, MYSM1, NAA35, NAB2, NBEA, NECAB1, NENF, NFE2L3, NIPA1, NKIRAS1, NLGN4Y, NMUR1, NOL8, NPIPA3, NUDT4B, NXF3, OR1F12, OR2T33, OR6C68, P2RY14, PACERR, PATL1, PDCD1LG2, PFN4, PIK3C2B, PIK3C2G, PITRM1-AS1, PLA2G4A, PLEKHA4, PLSCR2, PLTP, POLA2, POLR2J3, PPP1R3G, PRDM16, PRDM16-DT, PRDX1, PRKN, PROSER2, PSCA, PTGS1, PTPRE, PYROXD1, RAB5A, RAD21L1, RASA1, RASA2, RASGEF1A, REC8, RGS17, RGS9BP, RHBDF2, RLIM, RP1, RPL13P5, RPL23AP87, RUSC1, SAMD10, SAT1, SATB1, SATB2, SCAPER, SCN11A, SCN1B, SCN3B, SCRT1, SDHAP3, SEC14L3, SEC22B3P, SEC31A, SENP2, SEPTIN7P9, SERPINA3, SFMBT2, SH3BP5-AS1, SH3D19, SIK3, SLC12A3, SLC22A17, SLC22A6, SLC25A33, SLC25A53, SLC26A11, SLC35A2, SLC35F2, SLC4A3, SLC7A9, SLCO4A1, SLCO4A1-AS1, SNHG29, SNORA63, SNORA67, SNORD10, SNORD13, SNORD144, SNX7, SPACA5, SPIB, SPINK1, SPINK5, SPP1, SPRR1A, SPRR4, SPTY2D1, SRSF3, STARD4, TBC1D15, TBC1D23, TBC1D3C, TBC1D3I, TBC1D7-LOC100130357, TBCD, TCTA, TEDC2, TJAP1, TMEM158, TMPRSS11D, TP53INP2, TRAF5, TRIM67, TRPC1, UBE2V1, UFSP2, ULK2, USP45, VWA3B, WAKMAR2, WBP2NL, XCR1, YEATS2, YES1, YTHDC1, ZC3H3, ZFPM2, ZFR2, ZFYVE27, ZNF222, ZNF337-AS1, ZNF454, ZNF554, ZNF653, ZNF7, ZNF704 |
| darkgreen | ACP6, ADAMTSL4, ADGRG3, AGAP2, AIMP2, ALDOA, ALDOC, AMN1, ANKHD1-EIF4EBP3, AP1S2, AP2B1, AQP9, ARAF, ARID5B, ARMCX6, ASCC1, ATP6V0B, B3GNTL1, BIN3, BORCS5, BSG, BTLA, C2orf49, CACNA1E, CAP1, CASP7, CBX6, CCDC130, CCDC159, CCL20, CCR1, CD2BP2, CD300LB, CDKL3, CDKN1A, CEACAM3, CETN2, CHI3L1, CLDND1, CLK3, COPA, CPQ, CRTC2, CSGALNACT1, CTB-41I6.1, CYP27A1, CYTH1, DCP1A, DENND1C, DNAJA4, DSTNP2, DTX2, DUSP1, ECD, EDEM1, EFR3A, ELF1, ELMOD3, EMD, EPG5, ERGIC3, EWSR1, EZR, FAM174A, FAM228B, FBRS, FBXL13, FOXO3, GAB3, GBA, GHRL, GNS, GPX3, GUSB, HAX1, HBS1L, HCLS1, HIF1A, HLA-E, HP, HPS1, ICAM2, IL10RA, IL2RG, IL6R, INAFM2, ITPRIP-AS1, JMJD6, KAT7, KBTBD7, KCNJ15, KDM5C, KDM7A, KIF3B, KXD1, KYAT3, LAPTM5, LCP1, LINC00398, LINC00659, LINC00877, LINC00884, LINC01481, LIPN, LOC100419170, LOC101927723, LOC101927789, LOC101928361, LOC105371763, LOC105372716, LOC105376714, LOC105377156, LOC105378342, LOC107984247, LOC107984754, LOC107985047, LOC107985074, LOC107985392, LOC107985746, LOC107985939, LOC107986432, LOC107986477, LOC107986649, LOC107987223, LOC107987425, LOC284454, LOC388780, LOC441155, LPXN, LRG1, LSP1P5, LUCAT1, M6PR, MAP3K20, MARCHF3, MCOLN1, MELTF, MFF, MICU1, MIR7848, MKNK1-AS1, MLF2, NAPA, NCAPH2, NECAB2, NFE4, NOL12, OSTM1, PCBP1-AS1, PILRA, PIP4P1, PLEKHG2, PLK3, POTEE, PPL, PPP1R10, PRDM2, PSAP, PSEN1, PSMD8, RAB7A, RALGAPA1P1, RAPGEF1, RBM34, RESF1, RHOC, RNF13, RRN3P2, SEC61A1, SERPINB8, SFR1, SH3GL1, SHOC2, SIPA1L1, SIRT2, SIRT6, SLC11A1, SLC22A4, SLC30A7, SLC35A5, SLC39A1, SLC39A8, SLC6A6, SLC9A8, SNORA1B, SNRPB, SPART, SPATA13, SREBF1, SRRT, ST13P4, STAT3, STK3, STT3A, SUPT6H, SWSAP1, SZRD1, TBC1D10B, TECPR2, TFEC, TFIP11, TIMP1, TMEM140, TMEM164, TMUB2, TNF, TNFRSF8, TNFRSF9, TOR1AIP1, TP53BP1, TPT1-AS1, TRAF1, TRIM39, U2SURP, UBAP1, UBE2C, UBE2N, UBXN1, USB1, USP4, UTRN, VASP, VPS37B, WASHC2C, WBP2, WDR74, ZCCHC14, ZHX2, ZNF316, ZNF438, ZNF655, ZRANB2 |
| darkgrey | ABLIM1, ACADVL, ANAPC11, ANKRD13A, ANKRD44-IT1, AP4B1, APTR, ARID1B, ARID4A, ARIH2, ARMC2, ARPIN, ASAP1, ATG101, ATG12, ATP2A3, ATP5MC3, BCL2L11, BRCC3, BRD1, BTF3L4, C16orf72, CALR, CCDC125, CCNI, CCT6P1, CCZ1, CD84, CEACAM8, CEP95, CHD7, CITED2, CIZ1, CMIP, COX7A2, CPNE3, CRABP2, CRELD2, CSTF2T, CTBP1, DAXX, DCAF4L1, DCAF5, DCAKD, DDX20, DEFA1, DIDO1, DIMT1, DPEP3, DPM3, DPP7, DPYSL2, DRAM2, EFHD2, EIF3J, ELAVL1, ELMSAN1, EMB, ERLEC1, ETFBKMT, FAM174C, FBXO48, FGD5-AS1, FLVCR1, FNBP1, FUZ, GABPB2, GALE, GET1, GMPPB, GNAS, GOSR2, GPAT3, HERC4, HERPUD2, HMGB2, HNRNPA1P10, HNRNPD, HNRNPL, IGF2R, INTS1, IPO7, IQCE, ITCH, KCMF1, KCND3, KIAA1191, KLHDC8B, KLHL22, KMT2D, KTN1, LARP4, LARP4B, LDB1, LFNG, LINC01888, LINC02580, LIPT1, LMF2, LOC101929703, LOC102724200, LOC102724740, LOC102724955, LOC102725228, LOC105371100, LOC105378040, LOC107985944, LOC107986211, LOC107986526, LOC254896, LRCH4, LSM10, LUC7L, LYAR, LZTFL1, MAN2A1, MAP1S, MAP7D1, MAPRE3, MATR3, MDN1, MED31, MED7, METTL7A, METTL9, MIR1244-2, MKRN2, MPC1, MPP7, MPRIP, MRE11, MRPL35, MRPL51, MRPS6, MSL2, MZT2B, NBEAL2, NBPF26, NCLN, NDUFA11, NDUFA13, NDUFC1, NDUFS3, NET1, NFU1, NISCH, NOL7, NOTCH2, NPL, NUAK2, NUBP1, NUFIP2, NUP50-DT, NUP93, ORC3, PAICS, PAPOLG, PCBP1, PCNP, PDCD2, PDCD6IPP2, PDE12, PEAK1, PEX1, PEX5, PHC1, PHTF2, PLEKHG3, PNN, POLR2K, POLR2L, PPID, PPIE, PPP4R1, PPP4R2, PRDM1, PRKACA, PRKD2, PRKXP1, PRPSAP2, PSD, PSMD14, PTMA, PTMS, PYGL, QKI, RAB11A, RAB30-DT, RAB40C, RABGAP1L, RAC1, RAD21, RANBP1, RANGAP1, RAP1A, RARS1, RBMS1, RETN, REX1BD, RFX2, RIPK1, RNF11, RNPEPL1, RPL22L1, RPL23, RPL32, RPL7, RPL7L1, RPS25, RPS6, RRBP1, RSBN1, RSPRY1, RUNDC1, RYK, SCOC, SELENOH, SERBP1, SFT2D2, SFXN3, SIN3A, SIRT3, SLC15A4, SLC25A11, SLC35F5, SLC37A1, SLC5A3, SLC6A14, SLF2, SLTM, SNORA8, SNRPD1, SNX11, SNX5, SON, STT3B, STYK1, STYX, STYXL1, SUMO2, SYNE3, TAF1D, TET2-AS1, TEX261, TLE4, TMEM175, TMEM186, TMEM258, TMEM91, TRAPPC4, TRAPPC8, TRIM13, TSC2, TXK, UBE2G1, UBE2J1, UBL5, UBN1, UBXN6, UCHL3, USP22, UTP18, VAPB, VIM, WAC, WDR19, WDR26, WDR76, WSB2, YWHAB, YWHAE, ZC3H10, ZC3H4, ZCCHC17, ZCCHC8, ZDHHC3, ZMAT1, ZNF552, ZNF706, ZNF720, ZNF766, ZNF812P, ZYG11B |
| darkmagenta | ABCD4, ABRAXAS2, ACADM, ACSF2, ACTR1B, ADAP2, ADCY10, ADH5, AFDN, AFG3L1P, ALDH3A2, ALKBH7, AMACR, ANKRD34B, ANXA2P1, ANXA7, APPL1, ARFIP2, ARHGEF1, ARV1, ATP11A, ATP2B4, ATPAF2, ATXN7L1, BCORL1, BMPR2, BORCS6, BPI, BRF2, BRMS1L, BTD, C12orf43, C1orf35, C1QC, C20orf27, C22orf34, CCDC91, CCR2, CD24, CDCA8, CLK4, CNOT8, CNR2, COQ6, COX7A2L, CREB3L2, CUTALP, DCTD, DDI2, DDT, DHODH, DHX8, DMWD, DNAJB7, DNPH1, DYNLL2, EIF2B1, ELP4, ENKD1, ENO1-AS1, ENOPH1, ENTR1, ERP27, EXOSC2, F13A1, FAH, FAM220A, FBXO31, FCRL1, FEM1B, FHOD1, FOXM1, FXYD5, FZD1, FZR1, GALNT6, GCNA, GIT2, GON7, GPBP1L1, GPR157, GSDMB, GYS1, GZMB, H1-10, H2BC20P, H2BC21, H2BC8, H6PD, HACL1, HAGH, HAVCR2, HDHD5, HEATR6, HGS, HLA-DPA1, HMGB3, HMGCR, HMGN5, HNMT, HOXA-AS2, HRAT5, HRH4, IL1RL1, IL32, IMMT, IMPA1, IMPACT, ING1, KBTBD11, KDM1A, KDM4B, KHSRP, KIAA0930, KIAA1107, KLC1, KMO, KMT2A, LEAP2, LENG1, LETM1, LHPP, LIN37, LINC01003, LINC01948, LINC02033, LOC100506142, LOC100506302, LOC101926967, LOC101927344, LOC101927954, LOC101929464, LOC102723872, LOC105369536, LOC105369565, LOC105369968, LOC105370165, LOC105370489, LOC105371649, LOC105372497, LOC105372976, LOC105373262, LOC105375001, LOC105377155, LOC105377884, LOC107984834, LOC107984895, LOC107985235, LOC107985550, LOC107985567, LOC107985885, LOC107986596, LOC107986853, LOC112267956, LOC112268021, LOC112268292, LOC339803, LRFN1, LRR1, LRRC42, LSM4, LYL1, LYSMD2, MAP3K14, MAP3K7CL, MAPK7, MAPKAP1, MARCHF7, MBP, MCM5, MERTK, METAP2, MFN1, MFSD9, MICOS13, MIF4GD, MIR7-1, MKNK2, MPG, MRPL17, MRPL32, MRPS12, MRPS34, MS4A3, NATD1, NBPF12, NBPF20, NDUFB2, NKD1, NPM1, NUDCD3, NUS1, OIT3, OTUD4, PARP1, PATZ1, PCBD2, PDCD11, PHF13, PHF8, PIGP, PIK3CD-AS1, PIK3R6, PLAG1, PMVK, PNKD, POLK, POLR1D, POLR3B, POM121C, POP7, PRKCZ, PRKRIP1, PSRC1, PTPN6, PUF60, PUM3, RABGGTB, RASSF4, REPS1, RFWD3, RNF187, RNF4, RPAIN, RPH3AL, RRP7A, SCD, SCN9A, SCRN3, SEH1L, SELENOI, SELENOT, SGSM2, SH3BP1, SHPRH, SIL1, SLAMF6, SLC24A3, SLC25A29, SLC28A1, SLC37A2, SLC50A1, SMARCD1, SNHG19, SNORD49A, SNRNP40, SNW1, SORT1, SPPL2B, SRXN1, STON2, SUPT3H, SURF6, SUSD3, TARS1, TBCC, TBRG4, TGFBRAP1, TIMM21, TMEM121B, TMEM185A, TMEM242, TMEM39B, TMEM54, TMEM65, TOR2A, TRAF6, TRAPPC3, TRIM62, TSPAN14, TSR3, TTC39C, TTI1, TUBA4A, TXLNG, TYK2, UBA2, UBIAD1, URI1, USP19, USP37, USP6NL, VEZF1, VMO1, VPS51, WASH2P, WDR53, WFDC3, YAF2, YDJC, YIF1A, ZBTB47, ZFAND5, ZNF25, ZNF317, ZNF395, ZNF557, ZNF765, ZNF778, ZNF836, ZNRF1, ZSCAN25, ZSWIM7 |
| darkolivegreen | ABT1, ADIPOR1, AHCYL1, APRT, ARRB2, ARRDC3-AS1, AVIL, B4GALT5, BISPR, BTNL8, CCDC117, CCDC144B, CCDC18-AS1, CCDC57, CLIC1, CNPPD1, COL17A1, COPE, CRKL, CRYBB2P1, DCBLD2, DEDD2, DGAT2, DHRS1, DLEU7, DLG1, DSTN, FBP1, FCGRT, GALNT10, GOLGA2, GRB2, H1-0, H3-2, HIBCH, HLA-DRB4, HLA-G, HPS4, IER3IP1, IFT43, IL27, ING5, ITPKB-IT1, KAT5, KLHL9, KRBOX4, LOC100506282, LOC100996664, LOC102724050, LOC102724642, LOC105369669, LOC105373077, LOC105378969, LOC107985568, LOC107985946, LOC107986525, LOC285074, LOC730268, LOXHD1, LSP1, MAZ, MIAT, MIR3174, MLH1, MMP24-AS1-EDEM2, MRPL49, MYD88, MYOSLID-AS1, NADK, NBPF10, NIPAL4, NKIRAS2, NMRAL1, NMRK1, NOL10, NOL4L, NUCB2, PHLPP1, PI4K2B, PLK2, POGZ, POLH, PPME1, PPT1, PRKAA1, PRKAR1A, PRPS1, PTPN11, PWWP2A, RASGRP3, RBM12, RBMXL1, RBPMS, RNF34, RPL17, RPP25L, RRM2, SAE1, SASH3, SCARB1, SDHAP1, SETD3, SH2B3, SIGLEC7, SLC25A36, SMARCD3, SMIM35, SNORA63B, SNORD50A, STARD8, STK16, SYN2, TBC1D16, TENT5C, THAP4, TMBIM6, TMC4, TMED1, TMEM134, TOR1A, TSC22D4, UBR7, UCKL1, UNC13D, VSIR, YIPF3, ZBED6, ZEB2-AS1, ZNF396, ZNF451, ZNF587, ZNF613, ZNF646, ZNFX1 |
| darkorange | AASDHPPT, ACP1, AGMO, AMZ2, ANKRD6, ANP32A-IT1, ANXA4, AP5B1, ARHGAP17, ARHGEF6, ARNT, ATRAID, BRAP, BTK, C11orf42, CATIP, CCDC18, CDC73, CDH2, CDK4, CFAP54, CHORDC1, CHRNB1, CIB3, CLPX, CNPY2, COA3, COA5, COG5, COMMD9, COX15, CRYZL1, CUEDC1, CUL9, CXorf21, DCTN1, DDX46, DEGS1, DHX29, EBP, EIF2AK2, EIF3J-DT, ERLIN1, EXOC5, FAM104B, FAM117A, FAM185BP, FAM98C, FEZ2, FLJ37453, FRYL, G2E3, GDPD3, GIMAP2, GMFB, GNB4, GPATCH8, GRK5, H2BS1, H3C4, HPGD, HSPC102, ID2-AS1, ID3, IFT20, IFT88, ILKAP, INO80D, INPPL1, IREB2, ITGAL, ITPKB, JTB, KIAA0319L, KLF11, KLF16, LCMT1, LETM2, LINC00639, LINC01145, LINC01176, LINC01765, LINC01816, LINC01852, LINC02217, LMBR1, LOC100128770, LOC101927441, LOC101927898, LOC101928906, LOC101928963, LOC102724608, LOC105370201, LOC105370866, LOC105372233, LOC105373386, LOC105375061, LOC105377313, LOC105378669, LOC105378867, LOC107984529, LOC107986991, LRRC58, MAP3K3, MCRS1, MEMO1, MFSD14B, MGME1, MIRLET7A1, MRPL13, MRPS18A, MRPS18C, MT2A, MTIF3, NAP1L4, NCOR1, NDFIP1, NDUFAF3, NDUFB5, NFE2L1, NKAP, OSTF1, PARP12, PARP8, PEX11B, PGLYRP1, PGLYRP2, PHF3, PHKA2, PIK3CA, PIN4P1, PKNOX1, POLD3, PSMD4, PTGES3, RAB28, RBBP5, RBM42, RMDN1, RNF144A, RPL13AP5, RUNX3, SART1, SCAF4, SEMG1, SEPTIN9, SETDB1, SHFL, SKP1P2, SLAIN2, SLC20A2, SLC25A24, SLC9A1, SMIM20, SNHG12, SNHG9, SNRPB2, SOS1, SPATA33, SPECC1, SRGAP2B, TAF11, TAF8, TANGO2, TATDN3, TCEANC2, TIMM23, TIMMDC1, TMCC1, TMOD2, TNNI2, TRAF2, TRIM37, TSPAN16, TSR2, TTC31, UBXN2A, ULK1, ULK4, USP42, VIPAS39, VPS25, VPS26C, VPS4A, VSIG10, WASHC3, WDR13, ZBED5-AS1, ZC3HAV1, ZCCHC2, ZFYVE21, ZNF697 |
| darkorange2 | AAMDC, ACTA2, ADPRH, AGPAT2, ALG10, ANAPC15, ANAPC2, ANKDD1A, ANXA6, ARL5A, ASB3, ATP5F1A, BLCAP, C1QB, C9orf106, CA12, CARD6, CASZ1, CCDC122, CCDC153, CCL7, CD86, CENPW, CEP162, CHPT1, CHST2, CLC, CLNS1A, CMTM7, COG4, COQ3, CRADD, CREBZF, CREG2, CRISPLD2, CWF19L2, CX3CR1, CYP2D7, DDX47, DESI2, DPY30, DSC2, DSTYK, DUSP6, DYNC1I2, ETS1, EYS, FAM13A, FAM13A-AS1, FBXL18, GADD45A, GATD3A, GATD3B, GSTT1, GTF2E1, GUCY2C, H2BC11, HDDC2, HENMT1, HEY1, HIPK2, ICAM4, IFFO1, IMP4, ITPK1, KCNQ1, KIF9, KRT10, KSR1, LCLAT1, LINC00671, LINC01032, LINC01089, LINC01127, LINC01410, LINC02776, LIPA, LOC100288798, LOC100289511, LOC105369469, LOC105369811, LOC105370524, LOC105372503, LOC105375902, LOC105376805, LOC105378358, LOC107984990, LOC107987095, LOC283788, LOC389641, LRP1, MARCHF1, MARK4, MBOAT1, MCAM, MDH2, MIER1, MIR103A2, MKLN1, MPZL2, MRFAP1, MRPL47, MYH11, NCOA2, NFIC, NGDN, NME1, NSF, NUDT2, NUP153, PABPC4, PADI2, PAQR7, PCMTD2, PEX6, PGBD4, PGM2L1, PGRMC2, PHF11, PIGF, PIGK, PLOD1, PML, POLE, PPIL3, PPP1R18, PPP3CB, PRRC1, PRXL2C, PSMB9, PSMG2, PTPRS, PUS3, RAB19, RAD50, RAE1, RANBP10, RAP1GDS1, RGS1, RIPK4, RNF139, RNF216, RNLS, SEC11C, SESN3, SLC33A1, SLC9B2, SMC6, SNHG8, SNORD56B, SNORD99, SRPK2, STMN1, TAF1, TGFB1, THBS3, TIRAP, TMED8, TMEM161B, TMEM272, TMPO, TOX2, TP53, TPGS2, TPST1, TRAF7, TRIM24, TRIM27, TSN, TTC28-AS1, TXNL4A, UBA7, UBE4B, UQCC2, WDR41, WDR48, ZC3H14, ZER1, ZMYND8, ZNF3, ZNF487, ZNF75D |
| darkred | A2ML1, ABHD17B, ACSS2, ADGRE1, AEN, AKAP8, ANKRD13D, ANKRD36C, ANO10, AOC2, AP2S1, APPBP2, ARMH3, ARRDC4, ASAP1-IT2, ASB16-AS1, ASF1B, ATG16L1, AVL9, BAP1, BCO2, BMP6, BOLA2B, C2CD5, C5orf24, CALU, CAPN1, CAPN2, CARD14, CBY1, CCDC107, CCL23, CCT2, CEBPZOS, CIAPIN1, CISD3, CLASRP, CLDN4, CLEC16A, CLPB, CMBL, CNOT11, CR2, CTSH, CTSL, CYB561D1, CYCS, CYHR1, CYP20A1, DEFB4A, DELE1, DENND3, DNAJC10, DNAJC14, DUOXA2, DYNC1LI2, EBLN2, ECM1, EFTUD2, EID1, EIF2B5, EIF3F, ELP2, EPN1, ETHE1, EXOC8, F11R, FAM199X, FBXO28, FKBP3, FOXC1, GALNT2, GAPVD1, GLT1D1, GPR132, GRPEL1, GSKIP, GTPBP4, HAMP, HBEGF, HERC1, HGF, HOPX, HSD17B10, HTRA2, IFI27, IL23A, IL36A, IL36RN, INPP5A, INTS6L, ITFG2, ITGB8, ITIH2, KANSL3, KLK13, KLK7, KRT16, KYAT1, LARP1B, LEP, LINC01137, LOC100506358, LOC101926963, LOC101927131, LOC101928402, LOC102723729, LOC102725044, LOC105371131, LOC105372803, LOC105373126, LOC107984656, LOC107986469, LOC107987304, LOC112268058, LOC391322, LOC644634, LOC645513, LTN1, MCEE, MDP1, MEAK7, MED15, MED16, MED21, MIR5047, MKKS, MKNK1, MLLT1, MTHFD1L, MTMR10, MUL1, MYO10, N4BP2L1, NARS1, NBPF14, NCBP2, NDOR1, NDRG2, NDUFA9, NDUFV1, NELFCD, NF1, NLGN3, NOP58, NPIPB3, NPIPB5, NR1D2, NR2C2, NR2F1-AS1, NRAS, NSL1, OGA, OGFOD1, ORC2, OTOA, OTOAP1, OTULIN, PABPN1, PBXIP1, PELI1, PHACTR4, PI4KA, PIK3CB, PLD2, PLEKHM1, PMPCB, POLG, POLM, POLR1C, PRCP, PRSS27, PSMG3, PTPN2, RAB13, RBMS2, RGP1, RNASE7, RNF113A, RNF121, RNF40, RPL36, RPL4, RPN2, RPRD1A, RPS13, RPS5, RTL8C, S100A7, S100B, SCAP, SCEL, SDC4, SEC16A, SEMA3B, SERPINB4, SF3A3, SIK1, SLAMF1, SLC1A3, SLC25A46, SLC2A1, SLC38A1, SLC38A10, SLC38A2, SMPD2, SNORD14B, SNORD35B, SNX31, SOCAR, SPRR2A, SPRR2C, SPRR2E, SPRR2F, STAP2, STRN4, SYNCRIP, TARS2, TCP1, TMEM143, TMEM187, TMOD3, TMPRSS11B, TREML4, TSC1, TSPAN13, UBE2W, UBTF, UBXN11, UCK1, UNKL, UTP11, UXS1, VPS37A, VPS54, VTI1A, VWA5A, YBX3, ZC3H18, ZMYM5, ZNF385A, ZNF576 |
| darkturquoise | ACAD9, ADA, ADPRHL2, AGTRAP, AOAH-IT1, AP1G2, AP1M1, APLF, ATAD5, ATG9A, B3GNT8, BASP1-AS1, BBS9, BCL2L1, BCOR, BLOC1S3, C11orf71, C16orf70, C1QBP, C1RL-AS1, CAPZB, CCDC154, CCDC73, CCNYL1, CDKN3, CENPBD1, CEP295NL, CHD4, CHM, CLTCL1, CLUAP1, CNTROB, COL15A1, COPS7A, CRIM1, CRSP8P, CRTAP, CRY1, CTNS, DCUN1D5, DDX19A, DDX49, DENND6A, DGCR6L, DNAJC27, DPH3P1, DPP9, ENC1, ENGASE, ERLIN2, EXOSC1, FADD, FADS1, FAM122C, FAM20C, FAM219A, FASTK, FASTKD1, FHDC1, FKBP4, FKBP8, FKRP, FLII, GADD45GIP1, GAR1, GHRLOS, GSTCD, GTF2I, GUCD1, GUF1, H2AC21, HDAC4-AS1, HMBS, HPS3, HS1BP3, HSD17B12, IDNK, IFNGR1, IGHMBP2, IL10, INIP, IQCB1, ITGB4, JAG1, JAGN1, KATNB1, KCNE1, KCTD13, KLHL36, KLLN, LGMN, LIG3, LINC00677, LINC00852, LINC02576, LOC100129917, LOC100287896, LOC101927402, LOC101928465, LOC102724463, LOC102724985, LOC105369975, LOC105372165, LOC105372407, LOC105374466, LOC105376517, LOC105377023, LOC105377663, LOC105378586, LOC107984315, LOC107984387, LOC107985874, LOC107986862, LOC112268184, LOC221946, LOC646471, LOC729296, LRRC25, LRRC8B, MAD2L1BP, MAML1, MBTD1, MCFD2, MCM4, MED27, MIATNB, MICAL2, MIR4263, MIR548E, MOAP1, MRPL15, MRPS26, MSC, MTCH2, MTFR1, MTMR14, MYOSLID, NAPRT, NAPSA, NAXD, NCR1, NDST1, NECTIN4, NORAD, NPLOC4, NRIP1, OR4F16, P2RX4, PAPSS1, PAX8-AS1, PAXBP1, PCDH12, PCSK5, PDXDC1, PGAP6, PGM1, PHF6, PIAS4, PIDD1, PIGQ, PIK3R2, PKD1L3, PMS2CL, POFUT1, POM121, PPIL2, PPP2R5B, PREPL, PRKCE, PRUNE2, PTGER2, PTGR1, PTPRJ, PTRHD1, RAB44, RABGGTA, RAP2C-AS1, RCC2, REC114, RFC1, RFT1, RFX5, RGPD1, RGPD8, RILPL1, RIOK1, RMC1, RNASEH1, RNF25, RPTOR, RRAGA, S1PR3, SCML1, SDR39U1, SELENOO, SETD4, SETD6, SETMAR, SFXN4, SLC10A3, SLC16A5, SLC22A18, SLC23A2, SLC25A34, SLC35E1, SLC35E2A, SLC35E3, SLC4A2, SLC51A, SLC9A6, SLC9A7, SNHG16, SNORA104, SNORA70G, SPATA2, SPDYE1, SPG7, SPTBN1, SRPK1, SSSCA1-AS1, STARD7, SUMO3, SUSD1, SYNE1, SYNJ2, TADA3, TCEAL8, TCFL5, TCOF1, TENM1, TIMM10B, TMEM106A, TMEM115, TMEM189, TMEM218, TMEM248, TMEM43, TNNT1, TOMM34, TOP2A, TPCN2, TPM1, TXNDC16, U2AF1L4, UBOX5, UHRF1, UNC50, USP32P1, XKR3, XYLT1, YJU2, YLPM1, ZADH2, ZBTB2, ZBTB26, ZBTB48, ZCCHC3, ZFYVE9, ZNF121, ZNF155, ZNF160, ZNF182, ZNF189, ZNF324, ZNF397, ZNF425, ZNF506, ZNF507, ZNF622, ZNF675, ZNF680, ZNF821, ZSCAN29 |
| floralwhite | AFF2, ALDH6A1, APOOL, BAG5, BUD13, C12orf60, C5, CCDC30, CD38, CFLAR, COA6-AS1, CPNE5, CTR9, DDX19B, DHRSX, DYNC1H1, EDC3, FCN1, FRRS1, FSTL4, GDF11, GJB6, GORASP2, GPR183, HARS2, HAUS7, IFT46, IPO5, ITGB1, ITPKC, KDM2B, LINC00938, LINC00957, LOC100132057, LOC100996351, LOC101927202, LOC101927730, LOC101927770, LOC101928421, LOC101929767, LOC105369601, LOC105371036, LOC105376090, LOC105378647, LOC107985207, MINDY3, MTRR, MVB12A, NAPB, NCKAP5L, NDUFAF2, PAPLN, PARP11, PCBP3, PDIA4, PIK3IP1-AS1, PLCB3, PRDM4, PTPRC, PWWP3A, RANBP6, REXO1, RFFL, SERINC4, SERPINB2, SIRT4, SLC35D1, SPAG5-AS1, SUPT7L, TAZ, TFDP2, TGM1, TMIGD3, TMX3, TSTA3, TTC32, UBE2S, VENTX, ZNF138, ZUP1 |
| green | AAR2, ABCB6, ABHD4, ABHD8, ABI2, ABLIM3, ACOT1, ACVR1B, ACVRL1, ADAM15, ADAMTSL1, ADGRB1, ADGRB3, AFAP1L2, AHDC1, AJUBA, AKAP17A, AKR1B1, AKR1C4, ALDH5A1, ALG1, ALKBH8, ALPK3, ANKMY1, ANKS3, APOBEC3H, ARHGAP35, ARL4A, ASCL2, ASPHD1, ATP2B2, ATP2C2-AS1, ATP6V1C2, ATP6V1E2, ATP6V1FNB, AURKC, B4GALT7, BAALC, BAALC-AS1, BACH1-IT2, BACH2, BCAR3, BCAT2, BEX3, BIRC5, BNC2, BNIP1, BOD1, BORA, BTBD18, BYSL, BZW2, C11orf97, C15orf65, C19orf44, C19orf67, C1QTNF1-AS1, C1QTNF12, C5orf66-AS1, C8orf37, C9orf64, CA11, CA3-AS1, CAPN10-DT, CAPN14, CARNMT1-AS1, CAVIN1, CAVIN2-AS1, CBLN3, CBX2, CCDC157, CCDC80, CCDC9B, CCN1, CCND1, CD163L1, CD200, CD22, CD5, CD80, CDCA2, CDCA3, CDH1, CDH6, CDH7, CDK6, CDKN2B, CDO1, CELF5, CENPH, CENPI, CES1, CFAP43, CH507-9B2.8, CHIC1, CIART, COBLL1, COL16A1, COL1A2, COL4A2-AS2, COL6A1, COLQ, CPNE9, CRB2, CRHR2, CTHRC1, CYP2A6, DDR1, DEFB124, DEPDC1, DGKH, DHX37, DIAPH3, DIS3L2, DKK1, DLGAP5, DLL1, DLSTP1, DNER, DOCK3, DPYD-AS1, DSG2, DSG3, DST, DTNB-AS1, DUSP8, E2F6, EDDM3A, EGOT, EMC1, EML2-AS1, ENO2, EPB41L4A, EPB41L5, EQTN, ERI2, ESPL1, EXOC3L4, FABP4, FAM166A, FAM21EP, FAM25C, FAM25G, FAM83H-AS1, FAM87A, FAM87B, FAT1, FBXO10, FBXW7-AS1, FCHO1, FCRLB, FGF2, FHL1, FKBP1B, FKTN, FN1, FOXD3-AS1, FOXL1, FSCN1, FST, FUNDC2P2, FXYD1, FXYD6, GAS6, GAS6-AS1, GASAL1, GDPD4, GGT2, GJA1, GJC1, GMCL2, GMPS, GNG11, GNRHR2, GPLD1, GPM6B, GPR1-AS, GPR176, GRID2IP, GS1-124K5.4, H1-3, H2AC8, HCG14, HCRT, HDAC5, HEXA-AS1, HIF1A-AS1, HIGD1C, HLA-DOB, HLA-F-AS1, HLTF-AS1, HNF4A, HPN-AS1, HPS6, HPX, HRAT17, HSDL1, HSPA2, HTD2, HTRA4, IDH2-DT, IDH3A, IDUA, IFT172, IFT80, IGF2BP2, IGIP, IGLL3P, IGSF11, IL36B, IL9R, INMT, INTS14, IPO13, ITPKA, JRK, KATNAL1, KCNAB3, KCNH4, KCNJ16, KCNN1, KCTD7, KIAA0355, KIF20A, KIR2DL5B, KIZ-AS1, KLHL42, KRBA1, LAMA3, LAMC1, LAMTOR5-AS1, LANCL2, LAPTM4A-DT, LCN12, LEO1, LIMD1-AS1, LINC002481, LINC00426, LINC00487, LINC00578, LINC00597, LINC00640, LINC00856, LINC01010, LINC01011, LINC01181, LINC01184, LINC01283, LINC01291, LINC01293, LINC01471, LINC01602, LINC01657, LINC01932, LINC01963, LINC02150, LINC02246, LINC02621, LINC02679, LINC02762, LINC02800, LIPE, LLCFC1, LMOD3, LOC100287049, LOC100287290, LOC100505664, LOC100506076, LOC100652758, LOC100996404, LOC100996506, LOC101926886, LOC101927012, LOC101927021, LOC101927098, LOC101927245, LOC101927536, LOC101927712, LOC101927863, LOC101927947, LOC101927966, LOC101928000, LOC101928266, LOC101928398, LOC101928576, LOC101928711, LOC101928716, LOC101928824, LOC101929174, LOC101929178, LOC101929280, LOC101929574, LOC101929918, LOC101930026, LOC101930071, LOC102723468, LOC102723722, LOC102724475, LOC102724618, LOC102724638, LOC102724965, LOC102725238, LOC105369177, LOC105369323, LOC105369363, LOC105369378, LOC105369438, LOC105369484, LOC105369595, LOC105369745, LOC105369890, LOC105370178, LOC105370192, LOC105370457, LOC105370556, LOC105370887, LOC105370910, LOC105371126, LOC105371409, LOC105371468, LOC105371493, LOC105371642, LOC105371651, LOC105371710, LOC105371781, LOC105371857, LOC105371873, LOC105372282, LOC105372296, LOC105372555, LOC105372671, LOC105372709, LOC105372733, LOC105372785, LOC105372942, LOC105372960, LOC105373134, LOC105373155, LOC105373209, LOC105373514, LOC105373851, LOC105374382, LOC105374476, LOC105374534, LOC105374662, LOC105375028, LOC105375198, LOC105375448, LOC105375489, LOC105375509, LOC105375771, LOC105376034, LOC105376176, LOC105376197, LOC105376246, LOC105376410, LOC105376485, LOC105376627, LOC105376650, LOC105376934, LOC105376952, LOC105376995, LOC105377177, LOC105377277, LOC105377321, LOC105377492, LOC105377626, LOC105377871, LOC105377972, LOC105377973, LOC105377975, LOC105378328, LOC105378480, LOC105378662, LOC105379599, LOC105379827, LOC107984016, LOC107984043, LOC107984242, LOC107984264, LOC107984324, LOC107984340, LOC107984384, LOC107984473, LOC107984578, LOC107984661, LOC107984837, LOC107984840, LOC107984980, LOC107985040, LOC107985130, LOC107985139, LOC107985216, LOC107985410, LOC107985446, LOC107985524, LOC107985558, LOC107985595, LOC107985656, LOC107985870, LOC107985933, LOC107985936, LOC107985947, LOC107986086, LOC107986175, LOC107986332, LOC107986401, LOC107986630, LOC107986671, LOC107986872, LOC107987000, LOC107987010, LOC107987074, LOC107987107, LOC107987164, LOC107987276, LOC107987285, LOC110091768, LOC112267965, LOC112268015, LOC112268098, LOC112268143, LOC112268150, LOC112268296, LOC389834, LOC642361, LOC643387, LOC644090, LOC644135, LOC646736, LOC646762, LPAR5, LRPPRC, LRRC19, LRRC32, LRRN2, LSS, LY6K, LYG2, MAL2, MAP1LC3C, MAP3K14-AS1, MATN4, MC1R, MEIOC, MELK, MEP1A, MET, METAP1D, METTL14-DT, METTL15, METTL7B, MFAP5, MICA, MIP, MIR1-1HG-AS1, MIR200CHG, MIR4295, MIR5196, MIR548AT, MIR548AY, MIR583HG, MIR6772, MIR6854, MIR761, MIR8075, MMADHC-DT, MMP1, MMP2, MMP3, MOB1B, MOCOS, MOGAT1, MORN2, MOSMO, MRC2, MRM3, MRPL46, MSANTD2, MT1E, MT1F, MT1H, MT1HL1, MT1M, MTHFD1, MTMR4, MUTYH, MYCL, MYO15A, NAGS, NCAPH, NDUFB2-AS1, NEXN, NHP2, NID1, NMB, NOC3L, NOG, NOP14-AS1, NOP16, NPHP4, NPIPB6, NPIPB8, NR1I3, NRAV, NRIR, NSDHL, NUDCD1, NUF2, OR2A20P, OR7E24, ORC1, P2RY6, PACRGL, PAIP2B, PALB2, PARD3, PBX4, PCCA, PDE4DIPP1, PDGFC, PDGFRA, PEA15, PEPD, PER3, PET117, PGAP1, PHGDH, PHKG1, PIMREG, PKD2L1, PLCE1, PLS3, PMS2, PNLDC1, PNMA2, POLQ, PPIAL4A, PPP1R13L, PPP1R14B, PPP1R36, PRCD, PRG2, PRKAA2, PRKCI, PRKCQ-AS1, PRMT5-AS1, PRORSD1P, PRSS12, PRSS23, PSEN2, PSG4, PSG5, PTCH2, PTGES2, PTPN18, PTPRG-AS1, PTPRM, PXDN, PYGB, RAB38, RAB39A, RAD17, RAD51C, RALY-AS1, RBFOX3, RETSAT, RFC3, RFESD, RGS12, RHOBTB2, RND2, RNU5B-1, RNVU1-4, ROR1, RORA, RPL19P12, RPL39L, RRN3P1, RSPH10B, RTKN2, RXYLT1, S100A3, SAAL1, SAMD4A, SATB1-AS1, SCARNA6, SCARNA8, SCNN1G, SENP8, SERPINA6, SERPINB5, SERPINC1, SETDB2-PHF11, SFRP2, SGCB, SGK3, SGO2, SIGMAR1, SIRPG, SIRT1, SIX4, SLC13A4, SLC14A2, SLC16A12-AS1, SLC25A18, SLC25A19, SLC25A25-AS1, SLC26A2, SLC30A1, SLC39A10, SLC4A8, SLC5A6, SMC2, SMCR8, SMG7-AS1, SMIM38, SNAI1, SNAI2, SNHG1, SNORA1, SNORA27, SNORA40B, SNORA62, SNORA69, SNORD139, SNORD27, SNORD28, SNORD29, SNORD30, SNORD38C, SNRPA, SNUPN, SOD2-OT1, SPATA20, SPATA9, SPATS2, SPDYE17, SPINK4, SPOCK1, SPRY2, SPX, SRFBP1, SRGAP3, SRP54-AS1, SRSF10, SSTR2, STOX2, SULT6B1, SUPV3L1, SYT17, TAF5L, TBC1D3H, TBX18, TBX3, TEX26-AS1, THBS4, THSD1, TIMM44, TMEM109, TMEM200A, TMEM44, TMEM51, TMEM60, TMEM9B-AS1, TMOD4, TPI1P2, TPRA1, TRIB2, TRIM16, TRIM36, TSR1, TSTD3, TTC37, TTLL5, TUBB2A, TUSC3, TWNK, UAP1L1, UBE2E2, UBE2L5, UCHL1, UGDH-AS1, ULK3, UROS, USP30-AS1, USP50, UTP3, VSIG1, VTRNA1-2, WDR35, WFS1, WNT5A, WNT7B, WRN, YAP1, YPEL1, ZBTB20-AS3, ZC3H12C, ZCCHC4, ZFY, ZMIZ1-AS1, ZMYND10, ZNF124, ZNF23, ZNF235, ZNF257, ZNF283, ZNF286A, ZNF432, ZNF433, ZNF436-AS1, ZNF442, ZNF460-AS1, ZNF57, ZNF593, ZNF605, ZNF610, ZNF664, ZNF684, ZNF765-ZNF761, ZNHIT6, ZWILCH |
| greenyellow | A1BG, AARS1, ACAA2, ACTB, ADAMDEC1, ADAMTS5, ADAT3, ADGRF1, ADRA2B, AGFG2, AHI1, AKR1C1, ALG1L, ALYREF, AMY1C, ANKUB1, ANO9, ANTXR1, AOC1, AP1G1, APBA1, APOD, ARHGAP15, ARHGAP32, ATP13A2, ATP8B1, AURKAP1, B4GALT2, BAG2, BCL10, BRF1, BTG2, C1D, C1orf210, C22orf31, C3orf35, C4orf33, C5orf60, C9orf40, CAPZA1, CARNMT1, CATSPER3, CAVIN2, CBLB, CCDC163, CCDC184, CCNB2, CCNE1, CD55, CDC16, CDC25C, CDC26, CDC42, CDCA4, CDR2, CEACAM19, CENPS, CHML, CHMP6, CHRNA5, CHSY1, CHUK, CLCA2, CLDN12, CLEC18B, CNGA4, CNN3, CRACR2A, CRELD1, CSNK1A1, CSNK1G2-AS1, CSNK2B, CST6, CSTF1, CTB-30L5.1, CTH, CTIF, CYTH3, DCDC2, DDX17, DEPDC5, DGCR9, DGKQ, DMKN, DNAJC21, DNAJC9-AS1, DOCK1, DOCK5, DRAXIN, DRC7, DSCC1, DTD1-AS1, DTWD2, EEF1AKMT1, EFCAB3, EGR1, EGR2, EIF3B, ELOC, EP300-AS1, ERCC2, ERO1A, ERRFI1, FAM102B, FAM110D, FAM117B, FAM74A6, FAM86DP, FAM86HP, FANCD2, FAXDC2, FBLN5, FBXL19-AS1, FBXO40, FBXW9, FEN1, FIGNL2, FLJ44635, FOLR2, FRG1, FRG1EP, FRG1FP, FYN, G3BP2, GAGE1, GAGE13, GALNT16, GCGR, GNAI3, GOLGA2P5, GOLGA8N, GPR22, GPR89B, GTF2IP13, GTF3C4, GUSBP3, HBE1, HBG1, HBZ, HNRNPA2B1, HNRNPH1, HNRNPU, HSD52, HUS1B, ICA1L, ICK, IER3-AS1, IER5L, IGF2BP1, IGLL1, IKBKB, IQCN, IRAIN, ISL1, ITPK1-AS1, JMJD1C, JUN, JUND, KCNJ12, KHDRBS3, KIR3DL2, KLF13, KMT2C, LHFPL4, LILRA5, LINC00334, LINC00683, LINC00968, LINC00987, LINC00992, LINC01578, LINC01664, LINC01841, LINC01991, LINC02141, LINC02207, LINC02213, LINC02680, LINC02798, LINC02828, LINGO3, LITAF, LMO7-AS1, LOC100128568, LOC100996709, LOC101927432, LOC101927627, LOC101927858, LOC101928034, LOC101928096, LOC101928143, LOC101928352, LOC101928377, LOC101928572, LOC101929140, LOC101929297, LOC101929774, LOC101929823, LOC101929828, LOC102723444, LOC102723624, LOC102723899, LOC102724020, LOC102724438, LOC102724908, LOC102724968, LOC105369183, LOC105369673, LOC105369735, LOC105369820, LOC105370051, LOC105370190, LOC105370449, LOC105370532, LOC105371219, LOC105371229, LOC105371279, LOC105371406, LOC105372097, LOC105372479, LOC105372491, LOC105372618, LOC105372801, LOC105372817, LOC105372990, LOC105373191, LOC105373215, LOC105373547, LOC105373811, LOC105374376, LOC105374494, LOC105374593, LOC105374748, LOC105375417, LOC105375542, LOC105375751, LOC105375767, LOC105376058, LOC105376271, LOC105376829, LOC105376830, LOC105377319, LOC105377950, LOC105378100, LOC105378228, LOC105378448, LOC105379250, LOC105379525, LOC105379539, LOC105379565, LOC105379719, LOC105379839, LOC107983949, LOC107984035, LOC107984254, LOC107984333, LOC107984875, LOC107985072, LOC107985238, LOC107985529, LOC107986063, LOC107986198, LOC107986236, LOC107986265, LOC107986292, LOC107986350, LOC107986412, LOC107986426, LOC107986482, LOC107986556, LOC107986593, LOC107987245, LOC112268238, LOC613038, LOC646127, LOC646976, LOC728024, LOC93622, LRIF1, LRIG2-DT, LRRC34, LRRC4C, LRRTM2, LVRN, MACROD1, MAFB, MAFIP, MAP3K10, MAP3K6, MCL1, MED26, MEDAG, MICAL3, MIR10527, MIR302A, MIR302B, MIR302C, MIR302CHG, MIR302D, MIR4444-2, MIR4458HG, MIR4489, MIR4645, MIR646HG, MIR6858, MOGAT3, MPP4, MPZL3, MRPS25, MRTFA-AS1, MSS51, MT1B, MYL12A, MYL6, MYLK3, MYNN, NAA11, NACC1, NBAS, NEDD1, NEDD4, NEUROD1, NFIA, NONO, NPEPL1, NRXN1, NUTM2B-AS1, NXPH2, OCLN, OPA3, OVCH1, PAF1, PBX2, PCDH19, PDHX, PDIA5, PDLIM4, PF4V1, PFN2, PGM5-AS1, PHF20L1, PHTF1, PIGW, PIP5K1C, PKN2-AS1, PLCB4, PLK1, POFUT2, POLD1, POMGNT2, POMP, POTEM, PPIL1, PPIL6, PPM1L, PPP1CB, PPP3CA, PPP3CB-AS1, PPP3R1, PROK2, PRRC2C, PRXL2A, PSMA3-AS1, PSTPIP2, PTEN, PURB, R3HDM2, RABL2A, RAD51-AS1, RAPGEFL1, RASGRP1, RASSF6, RBM18, RBM5-AS1, RCC1, RCN1, RFC5, RGMB, RGPD6, RHBDL2, RHPN1, RNU6-2, RPS10, RRAS2, RSPH14, S100P, SARM1, SC5D, SCARNA2, SCYL2, SDF2, SEM1, SERPINE1, SIAE, SIK2, SKI, SLA2, SLC2A9, SLC30A10, SLC38A5, SLC6A13, SMIM24, SMTN, SNHG11, SNORA80E, SNORD22, SNORD31, SNORD49B, SNORD63B, SNX25, SPA17, SPATA45, SRF, SRGAP2D, SRP14-AS1, SRPX2, SRSF2, SSPO, SSRP1, STRAP, SUMO1, SVIL, SYP, TDRD6, TESK1, TEX12, THBD, TINCR, TLCD1, TLK1, TLR4, TM9SF3, TMED4, TMEM116, TMEM119, TMEM135, TMEM176B, TMEM18, TMEM191B, TMEM220, TMEM72-AS1, TOP1P1, TRA2A, TRAP1, TRIM55, TRIM66, TRPV5, TSC22D2, TTYH2, U2AF1, UAP1, UBE2B, UBXN8, UGCG, URB1, USP5, USP8, UVRAG-DT, WDR81, WNT4, WT1, YBX1, YWHAZ, ZBTB46, ZCCHC10, ZFC3H1, ZFPM2-AS1, ZGRF1, ZMYM1, ZNF208, ZNF263, ZNF318, ZNF423, ZNF493, ZNF514, ZNF528-AS1, ZNF577, ZNF582-AS1, ZNF618, ZNF627, ZNF717, ZNF750, ZNF761, ZNF773 |
| grey | ABAT, CORO1B, MVP, PPM1G, TCF7L2, TRNAU1AP |
| grey60 | ABCC1, ABI1, ACOT8, ACTR2, ADCY7, ADGRE3, ADRM1, AKTIP, ALCAM, ALG13, ALKBH5, ANAPC4, ANKFY1, ANKRD22, ANKRD49, APC, APIP, APOBEC3G, ARCN1, ARHGEF40, ARL15, ARL16, ARMT1, ARNTL, ARPP19, ARRDC1, ARSB, ATL2, ATL3, BECN1, BPGM, BRMS1, BTBD9, BTN2A1, C14orf119, C1GALT1, C1orf56, C3orf62, CAPRIN1, CASP1, CCDC174, CCNC, CCNL2, CCNT2, CCZ1B, CDC5L, CDK2AP2, CHST15, CKAP4, CNOT1, CNOT4, CSK, CTSS, CXXC1, CYB5D1, CYBA, DCTN2, DHX40, DNAJB11, DNAJC1, DNAJC7, DOK1, DOK3, DPY19L3, DUT, DYRK1A, EFCAB8, EIF2S3, EIF3C, EIF3CL, EIF3H, EIF4A2, EIF4G1, EIF4G3, ELF2, EPB41, EPC1, EVI5, EXOSC8, F2RL1, F8, FAM104A, FAM193A, FBXO38, FDPS, FEM1A, FES, FIBP, FICD, GAA, GABPA, GALNS, GALNT3, GAPT, GDI1, GET3, GHITM, GMIP, GOLGA3, GOLGA5, GRAMD1C, GTDC1, HAL, HAT1, HELB, HNRNPLL, HNRNPR, IKZF5, ING4, INO80, INTS12, ISCA1, ISCA2, ITGAX, JAK1, KAT8, KDM3A, KDM5B, KIAA0100, KIAA0556, KIF1B, KIF22, KLF7, LCORL, LEMD3, LGALSL, LINC00963, LINC02085, LINC02288, LNPEP, LOC101927759, LOC101928152, LOC105370579, LOC105371529, LOC107985087, LOC107985137, LOC107986707, LONP2, LRTOMT, LSM1, MAEA, MAPK8, MAPRE2, MARF1, MAST3, MBD1, MBD5, MCTP2, MDM2, MED19, MED30, MIA3, MIB1, MICU2, MID1IP1, MIR223, MIR3945, MRPL27, MSH6, MTF2, MXD3, MYDGF, MYO5A, N4BP2L2, NAA20, NAPG, NCBP3, NDUFS1, NECAP2, NPIPB12, NRDC, NRF1, NUCKS1, NUDT19, OAZ2, ODR4, OGDH, OS9, OSBPL2, OSBPL9, OSTC, OTULINL, P2RX1, PAM, PARP4, PARVG, PDAP1, PDCD10, PDCL3, PDIA3, PDZD8, PIAS1, PIK3C3, PIK3CG, PITPNM1, PLAGL1, PLCG1-AS1, POLI, POLR3GL, PPA2, PPIB, PPM1D, PPP1R11, PPP1R21, PPP2R3C, PPP6C, PRKCSH, PRKRA, PRMT2, PSMA3, PSMB2, PSMC2, PSPC1, PYCR2, R3HCC1L, RAB5B, RAD51B, RALB, RAPGEF2, RASAL3, RASGRP4, RBBP7, RC3H1, RCN3, REEP3, RETREG3, RNASE6, RNF7, RNPS1, RO60, ROCK1, RPF1, RPLP0, RPS8, RTN3, RUFY1, SAP30BP, SCAMP2, SCAND1, SCARNA20, SEC23A, SEC23IP, SF3B1, SF3B4, SGMS1, SIGLEC9, SIRT5, SLC25A3, SLC46A3, SMARCC1, SMC5, SMG1P2, SMG7, SMIM12, SMNDC1, SNRNP35, SPATA1, SRPRA, ST3GAL2, STAG2, STIM1, STK24, STRIP1, STRN, SUCLG1, SUGP1, SUPT20H, SVIP, TAF13, TAOK1, TAP1, TAX1BP1, TCAIM, TCEA1, TENT5A, TET2, TG, THEMIS2, TLE3, TMCC3, TMEM255B, TMEM81, TMF1, TNFAIP8, TNFRSF10B, TREML2, TRIM56, TRMT1L, TTF1, TTLL4, TULP2, TUT4, TXNL1, TYMP, UBE2E1, UBQLN1, UBR5-AS1, UHMK1, UHRF1BP1L, USO1, USP3-AS1, VPS13B, VSTM1, WDFY3-AS2, WDFY4, WDR20, WDR33, XAB2, XRCC6, YME1L1, YWHAQ, ZC3H7A, ZFYVE1, ZNF330, ZNF394, ZNF431, ZNF701, ZNRF2 |
| ivory | ABCE1, ABHD17C, ABI3, ACAP3, ACO1, ACTG1, ACTR1A, ACTR3-AS1, ADIRF, ADORA2B, ADORA3, ADSS2, AGAP11, AGPS, AHCY, AIMP1, AKAP12, AKR1C2, AKR1C3, ALG2, ALG8, ALKBH2, ALKBH6, ALPK1, ALS2, AMZ1, ANAPC1, ANAPC7, ANGEL2, ANKRD44-AS1, ANKRD52, AP1AR, AP1M2, AP3M1, APOA2, APOBEC3A, APOBEC3A_B, APOBEC3B, APOBEC3B-AS1, APOF, ARFGAP1, ARG2, ARHGAP24, ARHGAP29, ARHGAP5, ARHGEF2-AS1, ARL2, ARMC6, ARMC9, ARPC1B, ARRDC5, ARSA, ASPHD2, ASRGL1, ASS1, ATAD2, ATF3, ATG13, ATG3, ATG9B, ATP11A-AS1, ATP6V0E2, AURKA, AZI2, B3GNT9, BACE2, BAG6, BARD1, BATF2, BAZ2A, BBS7, BCAP31, BCDIN3D-AS1, BDNF, BET1, BEX2, BLM, BLMH, BLOC1S1, BMF, BPNT1, BST2, BTBD11, BTG3, BTN2A3P, BTN3A1, BTN3A2, C10orf105, C11orf68, C11orf96, C12orf4, C12orf45, C16orf96, C18orf21, C18orf61, C1orf116, C1orf162, C21orf58, C2orf74, C2orf88, C3orf86, C6orf132, C7orf61, C8orf33, CA2, CACNB4, CACTIN, CADM4, CALB1, CALML5, CAMSAP3, CARD16, CARHSP1, CASP4, CAT, CAV1, CBR1, CBWD2, CCDC120, CCDC124, CCDC181, CCDC194, CCDC28B, CCDC51, CCDC58, CCDC70, CCDC86, CCDC92, CCL3L1, CCM2, CCR4, CCT3, CCT6B, CD109, CD244, CD300H, CD33, CD74, CD99, CDC42P3, CDK5RAP2, CDKAL1, CEACAM5, CENPM, CENPP, CEP192, CERS2, CFL1, CFL2, CGNL1, CHAC2, CHMP3, CHMP5, CIDECP1, CISD1, CKAP2, CLCN2, CLDN15, CLDN9, CLEC12A, CLEC4D, CLIP4, CLN3, CLN6, CLTRN, CMPK2, CMTM4, CMTR1, CNGB1, CNIH3, CNOT3, CNPY3, CNTNAP1, COMMD7, COPZ1, CORO1A, CORO7-PAM16, CPED1, CPNE8-AS1, CPOX, CRB3, CREB3L1, CRHBP, CRLF2, CRTC3-AS1, CRYBG2, CSN1S1, CSNK1A1L, CTPS2, CTSO, CXCR2P1, CXorf40A, CYB5A, CYB5D2, CYB5R4, CYP19A1, CYP4F12, CYP51A1, CYP51A1-AS1, DAPK1-IT1, DAZAP2, DCAF16, DCLRE1B, DCPS, DDA1, DDIAS, DDN-AS1, DDX58, DDX60, DENND1B, DENND2B, DGUOK-AS1, DHH, DHRS3, DIXDC1, DLC1, DLST, DNAJA1, DNAJC15, DNAJC17, DNASE1L1, DNM3, DONSON, DPH5, DPY19L4, DPYD-AS2, DRAP1, DTX3, DUSP14, E2F4, EEF1G, EFNA1, EGFR, EIF3L, ELF5, ELFN1-AS1, ELOVL1, EMC6, EML6, ENOX2, ENPP2, EPB41L2, EPCAM, EPHA2, EPPK1, EPSTI1, ERAP2, ERCC5, ERGIC1, ERICH1, ERMAP, ERVK13-1, ESYT1, ETFB, ETV4, ETV6, EXOC3L1, EXOC4, EXOC7, EXOSC5, FAM160B2, FAM207A, FAM214B, FAM216A, FAM221A, FAM27E4, FAM3C, FAM81A, FAN1, FANCF, FBH1, FBXO9, FBXW4, FCGR1A, FCGR2C, FITM1, FLJ46875, FLT3LG, FOLR3, FOXN3, FRG1HP, FRG1JP, FRMD3, FRMD4B, FRS2, FSCN2, FTSJ1, FUS, FUT10, FUT8, FXYD3, GABRP, GALNT7, GAS2L3, GBP1, GBP1P1, GBP4, GBP5, GCHFR, GCNT1, GEMIN6, GHDC, GIT1, GLB1L, GNG10, GPATCH1, GPC4, GPHA2, GPHN, GPR107, GPR3, GRIK1-AS2, GRSF1, GTPBP8, GVINP1, GXYLT2, H3-3B, H3-5, HDAC7, HDDC3, HECTD4, HELZ2, HERC2, HERC5, HES1, HINFP, HINT2, HLA-C, HLA-F, HLA-H, HMG20A, HNRNPH2, HORMAD2, HOXA10-AS, HOXA9, HRK, HSH2D, HSPA5, IDS, IDS2, IFI35, IFI44, IFI44L, IFIH1, IFIT1, IFIT2, IFIT3, IFITM1, IFITM3, IFT27, IFT57, IL18RAP, IL6, IMP3, INHBA, INO80C, INO80E, INTS13, INTS4P2, INTS7, IPO11, IPO4, IRF1-AS1, IRF7, IRF9, IRS1, ISG15, ISG20, ISY1, ITLN2, ITM2A, ITPR3, ITSN1, JMJD7, JMJD8, JPX, KCND1, KDM8, KIAA0754, KIAA1614, KIAA1656, KIF3A, KITLG, KLHDC2, KLHDC7B, KNDC1, KNSTRN, KRTCAP2, L3MBTL2, LAMP3, LAPTM4B, LBH, LEKR1, LEPR, LGALS9, LGALS9B, LGALS9C, LILRB1, LILRB2, LILRB4, LINC00216, LINC00593, LINC00663, LINC00664, LINC00674, LINC00863, LINC00886, LINC00958, LINC01271, LINC01353, LINC01480, LINC01590, LINC01599, LINC01768, LINC01887, LINC01977, LINC01988, LINC02068, LINC02291, LINC02422, LINC02427, LINC02541, LINC02595, LINC02603, LINC02765, LINC02802, LINC02814, LIPH, LMAN1, LMLN2, LOC100128494, LOC100130331, LOC100131626, LOC100132077, LOC100289473, LOC100289495, LOC100506472, LOC101927080, LOC101927325, LOC101927377, LOC101927522, LOC101927830, LOC101927999, LOC101928047, LOC101928214, LOC101928234, LOC101928263, LOC101928343, LOC101928354, LOC101928424, LOC101928451, LOC101928474, LOC101928556, LOC101928731, LOC101928844, LOC101929512, LOC101929750, LOC101929819, LOC101929897, LOC102723458, LOC102723546, LOC102723648, LOC102723983, LOC102724008, LOC102724163, LOC102724197, LOC102724517, LOC102724572, LOC102724720, LOC102725258, LOC103344931, LOC105369214, LOC105369250, LOC105369332, LOC105369760, LOC105369767, LOC105369914, LOC105369950, LOC105370073, LOC105370265, LOC105370567, LOC105370651, LOC105370733, LOC105371064, LOC105371433, LOC105371636, LOC105371730, LOC105371849, LOC105371901, LOC105372238, LOC105372323, LOC105372427, LOC105372482, LOC105372493, LOC105372672, LOC105372930, LOC105372935, LOC105373033, LOC105373037, LOC105373207, LOC105373283, LOC105373480, LOC105373512, LOC105373780, LOC105373826, LOC105374171, LOC105374179, LOC105374264, LOC105374340, LOC105374764, LOC105374773, LOC105375020, LOC105375038, LOC105375433, LOC105375494, LOC105375523, LOC105376046, LOC105376137, LOC105376387, LOC105376481, LOC105376725, LOC105377035, LOC105377182, LOC105377199, LOC105377244, LOC105377676, LOC105377730, LOC105377771, LOC105378179, LOC105378218, LOC105378419, LOC105378858, LOC105378892, LOC105378913, LOC105378978, LOC105379013, LOC105379083, LOC105379091, LOC105379183, LOC105379524, LOC105379634, LOC107984203, LOC107984251, LOC107984302, LOC107984338, LOC107984389, LOC107984507, LOC107984679, LOC107984841, LOC107984847, LOC107984849, LOC107985007, LOC107985050, LOC107985184, LOC107985188, LOC107985364, LOC107985365, LOC107985402, LOC107985638, LOC107985678, LOC107985850, LOC107986067, LOC107986290, LOC107986433, LOC107986437, LOC107986495, LOC107986517, LOC107986522, LOC107986531, LOC107986589, LOC107986604, LOC107986631, LOC107986669, LOC107987075, LOC107987110, LOC107987119, LOC107987133, LOC107987181, LOC107987441, LOC112267861, LOC112267939, LOC112268036, LOC112268163, LOC112268272, LOC112268274, LOC112268467, LOC143666, LOC153684, LOC202181, LOC285819, LOC441204, LOC554206, LOC727896, LOXL1, LRPAP1, LRRC27, LRRC37A, LRRC37A8P, LRRC37B, LRRC8C, LRRK1, LTA, LUNAR1, LY6E, LY6G5B, MAB21L4, MAFK, MAGI3, MAN2C1, MANSC1, MAP3K12, MAP4, MARCKSL1, MARK2, MASP2, MASTL, MBOAT7, MCCC1, MCM2, MCRIP2, ME1, MED14OS, MED18, MEFV, MESP1, METTL16, METTL4, METTL5, MFAP1, MFSD10, MIIP, MIR10523, MIR17HG, MIR3124, MIR3188, MIR3654, MIR4257, MIR4259, MIR4271, MIR4470, MIR4713HG, MIR4738, MIR5187, MIR6859-1, MIR7109, MOB2, MOB3C, MOSPD1, MOV10, MPDU1, MPZ, MRPL1, MRPL12, MRPL42, MRPS14, MRPS24, MRPS30, MS4A6E, MTAP, MTREX, MTUS1, MX1, MX2, MYEOV, MYO1G, MYO5C, NAA10, NAA25, NAA40, NABP1, NABP2, NADK2, NAV3, NCKAP1, NCOA3, NDRG4, NEK1, NEK11, NEK4, NEK6, NELFA, NFIL3, NFYC, NHLRC3, NIBAN2, NKAPD1, NLRP6, NME4, NME6, NMU, NOC2L, NOD1, NOMO3, NOP2, NOS1AP, NPIPB11, NPIPB2, NPIPB9, NPTN-IT1, NQO2, NR2F2, NSFL1C, NSFP1, NSUN4, NT5C2, NTHL1, NUB1, NUBP2, NUMA1, NXT1, OAS1, OAS2, OAS3, OASL, OBSCN-AS1, ODF3B, OLA1, OTUB1, OVCA2, PANDAR, PAXIP1-AS2, PBDC1, PCGF5, PCIF1, PCYOX1, PCYT2, PDE6H, PDIA6, PDK4, PDP2, PDSS2, PERP, PFDN6, PGAM5, PGBD1, PGM5P2, PHLDB3, PHRF1, PI4KAP1, PIAS3, PIGV, PIPSL, PLAAT4, PLAAT5, PLAT, PLCD3, PLCG2, PLD3, PLEKHA1, PLEKHG6, PLPPR2, PMPCA, PNPO, POC1B-AS1, POGK, POLB, POLD2, POLD4, POLR2M, POM121L1P, POP5, POTEI, POU5F1P4, PP2D1, PPBPP2, PPM1K, PPP1R32, PPP1R35-AS1, PPP2R1B, PPP2R3A, PRADC1, PRAF2, PRDM11, PRIMPOL, PRNCR1, PRR13, PRSS3, PSENEN, PSMC6, PTGER4P2-CDK2AP2P2, PTPRF, PVR, PXT1, PYM1, QARS1, RAB31, RAB36, RAB39B, RAB43, RAB8A, RAD9B, RAP1GAP, RASA4DP, RASSF1-AS1, RBCK1, RBFOX2, RBM45, RECQL5, RGPD3, RGS2, RGS7, RIC8A, RICTOR, RIPK3, RNF138P1, RNF169, RNF215, RNF220, RNF5P1, RNGTT, RPA2, RPH3A, RPL13AP6, RPL15, RPL18A, RPL23AP53, RPP40, RRP15, RRP8, RSAD2, RSU1, RTP4, RUBCNL, RUSC1-AS1, RUVBL2, S100A2, SAMD9, SAMD9L, SAMHD1, SAP25, SAP30-DT, SAV1, SCAMP1-AS1, SCARB2, SCARNA5, SCARNA9, SCML4, SCNN1A, SDR42E2, SEC23A-AS1, SEMA6C, SEPSECS, SEPTIN10, SERPING1, SERPINI1, SH3PXD2A, SH3PXD2B, SHISA5, SHLD3, SHROOM4, SIK1B, SIVA1, SLC18A2, SLC1A5, SLC25A14, SLC26A4, SLC31A2, SLC37A4, SLC39A13, SLC49A3, SLC7A6OS, SLED1, SLURP2, SLX1A, SMAP1, SMARCAL1, SMC5-AS1, SMG5, SMIM22, SMIM4, SMIM5, SMTNL1, SMUG1, SMURF1, SNAPC4, SNCG, SNHG15, SNHG5, SNORA105C, SNORA12, SNORD46, SNORD50B, SNORD68, SNRPD3, SNX29, SORD, SORD2P, SOX15, SP2, SPATS2L, SPDYE5, SPINK8, SRD5A3, ST3GAL4, ST6GAL1, STARD13, STARD7-AS1, STBD1, STRADB, STX5, SUCLG2, SURF2, SUSD6, SYNGR2, SYT11, TAF1A, TAF1L, TAGLN2, TAPBPL, TBC1D14, TBC1D24, TBC1D3, TBC1D3D, TBC1D9B, TBL2, TBL3, TBX19, TCAP, TCEANC, TCF4, TCN2, TCTN1, TDRD7, TEC, TEFM, TEP1, TEPSIN, TEX14, TEX264, TFAP2A, TFAP2C, TFCP2, TFF1, TFPI, THOC7, TIMM10, TINF2, TM9SF1, TMED7-TICAM2, TMEM123, TMEM161B-AS1, TMEM184C, TMEM214, TMEM235, TMEM64, TMLHE-AS1, TNFSF13B, TNKS2, TNNT3, TOGARAM2, TOMM5, TOR4A, TPGS1, TPRG1, TPTE, TPTE2, TRABD, TRAFD1, TRANK1, TRIM14, TRIM2, TRIM21, TRIM22, TRIM38, TRIM69, TRIM72, TRPT1, TRPV2, TRUB2, TSPOAP1, TSSK3, TTC21A, TUBB6, TXLNA, TYW1, UBALD1, UBAP2, UBE2E4P, UBE2F, UBE2L6, ULBP3, UNC5A, UNG, UNK, UQCRH, UQCRHL, URB1-AS1, USP10, USP11, USP18, USP41, UTP4, UVSSA, VHLL, VPS16, VPS53, VSIG10L, WARS1, WASH8P, WASH9P, WBP11, WDR34, WDR70, WHRN, WNT2B, WWTR1, XPC, YJEFN3, ZBP1, ZDHHC16, ZFHX2, ZFP36, ZKSCAN4, ZMYM6, ZMYND15, ZNF133, ZNF195, ZNF341, ZNF384, ZNF420, ZNF439, ZNF440, ZNF480, ZNF483, ZNF546, ZNF559, ZNF578, ZNF581, ZNF687, ZNF724, ZNF767P, ZNF79, ZSCAN16-AS1, ZYX |
| lightcyan | ADNP-AS1, AGBL5-AS1, AIFM3, ALDH7A1, ALMS1, ALMS1-IT1, ALOX12P2, ANGPTL7, APOE, APOL3, AQP3, ARHGAP31-AS1, ASB1, BHLHE41, BOK, C1QTNF7-AS1, C4B, C6orf52, CABLES1, CACNA2D2, CADM1, CAVIN3, CCDC171, CCDC6, CCDC62, CD27-AS1, CDT1, CEBPB-AS1, CELA1, CEP131, CEP85, CGA, CIDEB, CLBA1, CLEC18C, CLEC4G, CLRN1-AS1, COBL, COCH, COL13A1, COX6B2, CPHXL, CRTC1, CYB561A3, CYP7A1, D2HGDH, DCST1-AS1, DCTPP1, DFFA, DHDDS-AS1, DHRS4L1, DNAH2, DNAJC18, EDARADD, EIF2D, ELOA-AS1, ELOVL7, ENOSF1, ESR1, FAM111A-DT, FANCL, FAS-AS1, FBXO21, FLJ13224, FLJ34503, FRAS1, FSIP1, GALNT4, GDF9, GEMIN8, GFOD1, GGT5, GIMAP5, GJA9-MYCBP, GJB7, GLYCTK, GNA11, GPATCH4, GPR87, GPT2, GSDMC, GSDME, GSN-AS1, H2BC9, HPD, HS3ST2, HSPE1-MOB4, IBSP, ID1, INAVA, INO80B, KCNK13, KDELR3, KIR2DS5, KLC4, LCA5L, LHFPL3-AS2, LIMCH1, LIN7B, LINC00111, LINC00999, LINC01133, LINC01220, LINC01232, LINC01344, LINC01354, LINC01431, LINC01617, LINC01714, LINC01764, LINC01907, LINC02212, LINC02376, LINC02539, LINC02617, LINC02669, LINC02777, LIPG, LMNA, LOC100288175, LOC100303749, LOC101926907, LOC101927278, LOC101927506, LOC101927588, LOC101928080, LOC101928466, LOC101929109, LOC101929285, LOC101929506, LOC102723795, LOC102723798, LOC102724727, LOC102724813, LOC102725023, LOC105369747, LOC105369933, LOC105369969, LOC105370062, LOC105370148, LOC105370397, LOC105370791, LOC105371597, LOC105371668, LOC105372141, LOC105372506, LOC105372706, LOC105373105, LOC105373171, LOC105373184, LOC105373421, LOC105373880, LOC105374013, LOC105374776, LOC105375363, LOC105375670, LOC105375782, LOC105375833, LOC105375839, LOC105375969, LOC105376033, LOC105376050, LOC105376228, LOC105376332, LOC105376731, LOC105376818, LOC105377106, LOC105377342, LOC105378018, LOC105378255, LOC105378305, LOC105378736, LOC105379053, LOC105379351, LOC105379395, LOC107984248, LOC107984263, LOC107984299, LOC107984329, LOC107984450, LOC107984475, LOC107984940, LOC107985256, LOC107985388, LOC107985898, LOC107986064, LOC107986129, LOC107986677, LOC107986762, LOC107986788, LOC107986954, LOC107987129, LOC107987132, LOC107987224, LOC107987250, LOC112267863, LOC112268069, LOC112268195, LOC400684, LPAR3, LPP-AS2, LRIG1, LRRC36, LRRC63, LRRC8D, LRRN4CL, LY9, LYPD4, MAF, MALL, MAPK6-DT, MFSD14C, MMP21, MRPL24, MVB12B, NANOG, NCKIPSD, NEURL4, NICN1, NLRP2, NRP1, NRP2, OR2A1-AS1, ORC6, OSBPL3, PALM2AKAP2, PCAT2, PCDH7, PCNX2, PCOLCE-AS1, PIR, PLGLB1, PMP22, POLR1B, PPP4R4, PRG1, PRR16, PRR7-AS1, PTMAP11, RAB34, RAB40AL, RABEP2, RAD9A, RANGRF, RNF213-AS1, RPS10P7, RPS18, RRN3, S1PR1, SBNO1-AS1, SCARNA17, SCN8A, SCUBE2, SERF2-C15ORF63, SERHL2, SGSM1, SLC22A14, SLC27A5, SLC2A14, SLC3A1, SLC44A3-AS1, SLC9A2, SLX1A-SULT1A3, SMPDL3B, SMYD3, SNORA11F, SNORA15, SNORA59A, SNORA68B, SNORD38A, SNORD3I, SNORD5, SNRPN, SNX24, SORBS2, SPACA6P-AS, SPAG5, SPARC, SPRY1, STK32B, SUCNR1, TCIM, TEX43, THAP7-AS1, THSD7A, TIMM8A, TMCO2, TMEM176A, TMEM212, TMEM217, TMEM63B, TNFRSF11A, TNFRSF18, TNNI1, TNS1, TOMM22, TRIM65, TRIM74, TSNARE1, TSPAN4, TTC39A, TULP3, TWIST1, UBD, UBE2NL, USP20, VPS45, WDR66, WEE2, ZBTB39, ZNF221, ZNF251, ZNF341-AS1, ZNF460, ZNF696, ZNF702P, ZNF703, ZNF713, ZNF875, ZRANB3, ZWINT |
| lightcyan1 | ACBD6, ACLY, AEBP2, AGTPBP1, AK9, AOC3, APAF1, ARHGAP26-IT1, ARHGEF7, ARID2, ARID4B, ARMC7, ASH1L, ATP11B, ATXN2, AUH, BMS1P20, BRD8, BTG1, C1RL, CABIN1, CAPZA2, CBR4, CC2D2B, CCDC115, CCDC186, CCDC59, CCDC7, CCDC88A, CCDC88B, CCNG1, CCNY, CD99L2, CDK14, CHCHD2, CHD3, CHRAC1, CKS1B, CLK1, CNEP1R1, CNNM2, CNOT2, COA1, COG3, COL9A3, COMMD8, COPB1, COQ10B, COX6C, CPSF3, CPT2, CREBBP, CRNKL1, CSF1R, CSNK1G1, CTNNB1, DAPK2, DCAF12, DCLRE1C, DEDD, DERL2, DGKG, DHX30, DKC1, DMAC2, DNASE1, DPM1, DUSP18, DYM, DYNC1LI1, EBPL, EIF1AD, EIF1B-AS1, EMC9, ERAP1, ERC1, ESRRA, EXOC1, FAM118B, FAM120A, FAM217B, FAM27C, FBXO11, FBXW5, FCHSD2, FRA10AC1, GALNT1, GALT, GATC, GDPGP1, GIN1, GPI, GSE1, H2AZ2, HACD4, HEBP2, HNRNPH3, HSD17B4, IER2, IFNAR2, INPP1, INSC, INTS6, ITGAM, JAK2, KANSL2, KAT6B, KBTBD3, KCNQ1OT1, KDELR1, KDM6A, KIAA2026, KIN, KLHL28, KNTC1, KRCC1, LINC00324, LINC01359, LOC101929185, LOC101929289, LOC105369180, LOC105371088, LOC105373064, LOC105375361, LOC105375492, LOC105376844, LOC105378967, LOC107986455, LOC107986463, LOC107986710, LOC107986856, LOC107987081, LOC112268042, LOC112268460, LRCH3, LRRC37A2, LRRC47, LRRC75A, LRRC8A, LSM14A, LTA4H, LTB4R, MARCHF6, MARK3, MAST4, MCM7, MDM4, ME2, MED17, MEF2A, MIA2, MIR29B2CHG, MPEG1, NASP, NDUFB9, NEMF, NFATC3, NIN, NUDCD2, ORAI1, PAN3, PANK2, PANK3, PAPOLA, PAQR6, PDE4D, PDE7A, PFKFB4, PHF12, PHF2, PHIP, PIBF1, PICALM, PIH1D1, PPP1R3B, PRPF3, PRPF38A, PRPF4, PRPF6, PSMD1, PTP4A2, PUM1, PUM2, RAB35, RALGPS2, RANBP9, RAP2B, RB1CC1, RBM25, RBM47, RBM7, RERE-AS1, RNF146, RNF166, RPGRIP1, RPL18, RPL19, RPL23AP82, RPP30, RPS21, RPS6KA3, RSRC1, RTCA, RTF1, RUNX1-IT1, SDHAF2, SEL1L, SENP7, SETD1B, SFI1, SFXN1, SH3KBP1, SLC22A1, SLC25A30, SLC25A40, SMG1, SNAI3, SNORA72, SNRPD2, SNX14, SP3, SP4, SPCS3, SRBD1, SREK1, SRSF6, SRSF7, SS18, SSNA1, STAU1, STK17A, STX16, STXBP3, SUZ12, SYNE2, SYNJ1, TATDN1, TBC1D22A, TGFBI, TMEM181, TMEM183A, TMEM92-AS1, TMTC3, TNFAIP8L2, TPM3P9, TPR, TRIM59-IFT80, TRIP11, TRIR, TTC9, UBAC1, UBR5, UGGT1, VAC14, VCPKMT, VTA1, WAPL, WASHC4, WDR25, WDR55, XPO7, XRCC5, YARS1, YTHDF1, ZC3H8, ZDHHC13, ZNF146, ZNF148, ZNF445, ZNF66, ZNF746, ZNF91 |
| lightsteelblue1 | AKT2, APOO, ATP5F1D, BABAM1, BAIAP2, BTBD19, C19orf38, CCDC149, CD93, CDIPT, CEBPE, CMTM1, COPS8, CST3, CYP4F3, DBN1, DDX23, DNAH14, EIF4G2, ETFA, EXOC2, EXT1, F5, FAF2, FBXW2, FOXN2, FOXP1-AS1, FRMD4A, GMNN, GNG7, HYPK, IL15RA, ING3, INSIG2, ITGA6-AS1, KLHL18, LILRB3, LINC00173, LINC00189, LINC02205, LINC02285, LOC102723663, LOC102724404, LOC105372272, LOC105374333, LOC105374746, LOC105375655, LOC105377272, LOC105377587, LOC105379308, LOC107984521, LOC107986939, LOC112268252, MAGIX, MAML3, MCU, MEAF6, METAP1, MME, PAIP1, PIGG, PLIN4, POLG2, PRKAR2A, PRR5L, PRSS36, PTP4A3, REXO5, RHOU, RNASEH2C, RNU12, RPL41, RUNX1, SECISBP2L, SERPINB1, SH3RF3, SLC24A4, SLC35C2, SNX18, SPOCK2, TBC1D20, TERF2, TIGD7, TMED9, TMEM185B, TNIP3, TOB2, TRMT6, TSPYL1, VCAN, ZNF668, ZNF789, ZNF791 |
| lightyellow | AAGAB, AANAT, ABITRAM, ACBD4, ACOD1, ADRB2, AIRN, ANKRD18CP, AP4M1, ARHGEF5, ARMH2, ATG4C, ATP1A3, ATP23, ATPAF1, BMS1P14, BORCS8-MEF2B, C12orf75, C2orf66, C5orf67, CACNA1C, CAPN3, CCDC183, CDH4, CDK5RAP1, CFAP53, CH17-340M24.3, CHRFAM7A, CLIC2, CMTR2, CRAT37, CSTF3-DT, CXXC5, CYP2A7, DCLK1, DCXR, DECR2, DISP1, DNAAF5, DNAJA3, DNAJC19, DRG1, EIF5A2, EMILIN2, F8A2, F8A3, FADS2, FAM169B, FAM185A, FAM222B, FAM223A, FAM229A, FAM85A, FBXO44, FBXO8, FLVCR2, G2E3-AS1, GLI4, GOLGA2P6, GPRACR, GSTT1-AS1, H4C14, HACD3, HHIPL2, HIKESHI, HPR, KCNB1, KCNJ2-AS1, KCNQ5, KIFC1, LANCL1, LINC00417, LINC00431, LINC00690, LINC00881, LINC01730, LINC01800, LINC01881, LINC02320, LINC02799, LIPE-AS1, LOC100129434, LOC100507373, LOC101926969, LOC101927060, LOC101927283, LOC101927764, LOC101927814, LOC101928089, LOC101928387, LOC101928487, LOC101929208, LOC101929599, LOC102723439, LOC102724046, LOC102724238, LOC102724751, LOC102724889, LOC103021295, LOC105369246, LOC105370105, LOC105370543, LOC105370974, LOC105371223, LOC105371348, LOC105371430, LOC105371472, LOC105371507, LOC105371796, LOC105371941, LOC105372414, LOC105373759, LOC105373836, LOC105373881, LOC105374907, LOC105375045, LOC105375384, LOC105375591, LOC105376211, LOC105376412, LOC105376447, LOC105377826, LOC105379272, LOC105379355, LOC105379382, LOC105379409, LOC107984334, LOC107984482, LOC107984778, LOC107984952, LOC107985269, LOC107985521, LOC107985953, LOC107987007, LOC107987112, LOC107987234, LOC107987254, LOC107987401, LOC112267885, LOC112268203, LOC112268253, LOC112268422, LOC200772, LOC389765, LOC613206, LRRC14, LUZP1, MBOAT4, MED12L, MFF-DT, MGC12916, MICALL2, MIR1304, MIR4530, MIR497HG, MIR6774, MMD, MPPED2-AS1, MSRB3, MYO9A, NAP1L5, NEURL1B, NFIB, NFYC-AS1, NPRL3, NRARP, NRL, NSMCE3, OCRL, ORAI3, PANK4, PARM1, PHLDB1, PI16, PINLYP, PKP2, PLAAT3, PMS2P4, POP4, POT1, PPIP5K1, PPOX, PROSER2-AS1, PRR3, PSORS1C3, RARA-AS1, RBFOX1, RGS10, RNF216-IT1, RNVU1-19, ROBO4, SAMD14, SCARNA16, SCARNA28, SELENOOLP, SEPTIN6, SEZ6, SGCA, SGO1, SH3RF3-AS1, SKA1, SLC39A3, SLC66A3, SNORA18, SNORA23, SNORA28, SNORA51, SNORD129, SNORD19B, SNORD3B-2, SNORD72, SOX9, SPC25, SPIN1, SRGAP2, STAB2, STK31, SYN3, TBC1D32, TEKT2, TF, TMEM229B, TMEM63A, TNK2, TNRC6C-AS1, TPH1, TRIM39-RPP21, TROAP, TSSC4, TTK, TUBG2, UBE2D3-AS1, ULK4P2, VASH1-AS1, VGLL4, WBP11P1, WDTC1, ZBED1, ZNF346, ZNF426, ZNF451-AS1, ZNF461, ZNF561-AS1, ZNF587B, ZNF629, ZNRF2P1, ZSCAN12 |
| magenta | AATK, ACAT2, ACD, ADAM8, ADCY4, ADPRM, AFTPH, AGAP3, AGO2, AGO3, AK4, AKIRIN2, AMY2B, ANKLE2, ANKRD12, ANXA5, AP3D1, APBB3, ARAP1, ARFGEF1, ARHGEF2, ARIH1, ARL6IP1, ARL8A, ARL8B, ASAH1, ASL, ATP13A3, ATP2C2, ATP6V0D1, ATP6V1B2, ATP6V1C1, ATP6V1D, ATP6V1F, ATP6V1H, ATXN2L, ATXN7L3, AZIN1, B4GALT4, BABAM2, BACH1, BAX, BCLAF3, BHLHE40, BHLHE40-AS1, BIRC2, BNIP3, BNIP3L, BORCS8, BRD2, BRK1, BTAF1, BTBD2, BTN2A2, BZW1, C15orf48, C1orf122, C3, CAMKK2, CAMTA2, CAPG, CASP9, CC2D2A, CCDC32, CCDC47, CCDC84, CCDC93, CCDC97, CCNT1, CCR5AS, CD14, CD163, CD274, CD58, CD63, CD68, CD82, CDC42EP3, CEP170, CEP68, CERK, CFAP70, CHAF1A, CHMP2B, CHMP4B, CLCN6, CLIC4, CLTA, CLTC, CMTM6, COMMD4, CREB1, CREB3, CREBL2, CS, CSF1, CSF3, CSF3R, CSGALNACT2, CSNK1G2, CSTB, CTSA, CTSD, CXCR4, CXorf65, CYB5R3, CYBB, CYC1, CYFIP2, DAP, DAPK3, DCAF13, DDX18, DDX39A, DDX41, DENND2D, DENND5A, DHX15, DHX38, DIAPH1, DNAAF1, DNAJC5, DNM2, DOCK10, DOCK2, DOCK4, DOCK4-AS1, DOT1L, DRAM1, DSE, DTNB, DTNBP1, DUSP10, DUSP16, DUSP3, DUSP5, EAF1, EEF1D, EEF2, EIF1B, EIF2AK3, EIF4EBP3, EML4, ENO1, ENO3, ERBIN, ERN1, ESCO1, ETV3, ETV5, F3, FAM107B, FAM151B, FAM162A, FAM172A, FAM50A, FBXO34, FGR, FHL3, FLCN, FLNA, FNDC3B, FNIP1, FOXO3B, FUT11, FXR1, GABARAPL2, GALC, GAPDH, GBE1, GGT1, GK5, GLMP, GNB1, GNG2, GNPDA1, GPATCH2L, GPBP1, GPCPD1, GPR108, GPR68, GPR84, GPX1, GRAMD1A, GRINA, GRN, GSAP, GSTO1, GTF2A1, GTF2A2, GTPBP1, GUK1, GUSBP11, H2AC20, HBP1, HECA, HEIH, HELQ, HEXB, HIF1A-AS2, HIF1A-AS3, HILPDA, HINT3, HIVEP2, HK2, HMGA1, HMGN2P46, HNRNPC, HNRNPF, HOMER3, HPCAL1, HS3ST3B1, HSCB, HSD11B1, HSPA4, HSPB1, HUWE1, IDH2, IFNGR2, ILRUN, IMPDH2, INPP5K, INSIG1, IRAK3, IRGQ, ITGA5, JARID2, KCNAB2, KCNK6, KDM2A, KHNYN, KIFC3, KLF6, KLHL24, KMT2E, KPNA1, KRAS, LAMB1, LDHA, LGALS3, LGALS8, LIMS1, LIMS3, LIMS4, LIN54, LINC-PINT, LINC00239, LINC00243, LINC00476, LINC00513, LINC00654, LINC01093, LINC01303, LINC01503, LINC01762, LINC02154, LOC100287072, LOC100506990, LOC101927993, LOC101928762, LOC102724660, LOC102724850, LOC105371912, LOC105372630, LOC105374181, LOC105374369, LOC105375070, LOC105375304, LOC105376626, LOC105377347, LOC105378230, LOC105379094, LOC107985366, LOC107985926, LOC107986127, LOC107986161, LOC107986655, LOC107987057, LOC107987150, LOC112268199, LOC112268235, LOC339192, LOC400499, LONP1, LONRF3, LPCAT1, LPP, LRRC6, LUC7L3, LYRM7, MAD2L2, MAFG, MALAT1, MAN2B1, MAP2K1, MCRIP1, MCTP1, MEPCE, METTL23, MFSD1, MGAT1, MGLL, MIR29B1, MIR3198-1, MIR3671, MITD1, MMP2-AS1, MOB3A, MROCKI, MRPL18, MTF1, MTMR3, MVD, MYBPC3, N4BP1, NANOS3, NAP1L1, NARS2, NBPF15, NBPF19, NBPF25P, NBPF8, NDRG1, NDUFAF1, NDUFAF7, NDUFV2, NEK9, NEU1, NFAT5, NFKB1, NFKBIE, NFX1, NGLY1, NINJ1, NIPA2, NLN, NOCT, NOTCH2NLA, NOTCH2NLB, NOTCH2NLC, NPC1, NPEPPS, NR4A1, NSMCE2, NUP58, NUP98, NXF1, ORM1, ORM2, OSBP, OSGIN1, OSGIN2, OXA1L, P4HA1, P4HB, PARP6, PCM1, PCTP, PDE4DIP, PDK1, PDLIM7, PDXK, PEF1, PER1, PFKL, PGAP2, PGS1, PI3, PI4KB, PIK3AP1, PIK3C2A, PIK3IP1, PIK3R5, PIP4K2C, PKM, PLA2G4C, PLAGL2, PLD1, PLEKHB2, PLEKHF2, PLEKHJ1, PLEKHM3, PLEKHO2, PLIN2, PLPP3, PNKP, POLR2A, POR, POU5F2, PPARD, PPFIA1, PPP1R3E, PPP2CA, PRKCD, PSMD13, PTAFR, PTGER4, PTK2B, PTPN12, PTPN23, PXK, RAB10, RAB20, RAB21, RAB22A, RAB32, RAB3GAP2, RAB5C, RABL3, RALGAPA2, RALGDS, RAP2C, RAPGEF6, RASGEF1B, RASSF5, RCBTB1, REL, RELB, RELT, REV1, RGPD4, RGPD5, RHOA, RHOH, RIMKLB, RIOK3, RIPK2, RIPOR1, RLF, RNF175, RNMT, RPF2, RPL10, RPS15AP10, RPS6KB1, RRAGD, RRNAD1, RRP1, RTN2, SAFB, SAT2, SCARF1, SCPEP1, SEC22B2P, SEC24A, SEC24C, SEC24D, SEMA6B, SEPTIN2, SERPINB6, SETDB2, SGSH, SGTB, SH3D21, SHLD2P3, SIAH1, SIGLEC14, SIGLEC5, SIRPA, SKIV2L, SLAMF7, SLC11A2, SLC12A6, SLC25A13, SLC30A4, SLC43A3, SLC49A4, SLC4A1AP, SLC7A5P2, SLC7A7, SMG9, SNAPC1, SNHG32, SNIP1, SNN, SNORA20, SNORA70, SNX10, SNX8, SPATC1, SPHK1, SPINT2, SPNS1, SPPL2A, SRA1, SRP54, SRSF12, SSR2, STARD3, STARD3NL, STAT5A, STAT5B, STK10, STX11, SUCO, TAB2, TACC3, TANC2, TANK, TATDN2, TBC1D2, TCIRG1, TECPR1, TERF2IP, TES, TET3, TEX10, TIGAR, TIMM17B, TLR2, TMBIM1, TMC8, TMEM120A, TMEM138, TMEM205, TMEM222, TMEM38B, TMEM41B, TMEM50B, TMEM68, TMEM88, TMEM92, TNFRSF10A, TNFRSF10D, TNFRSF14, TNFSF15, TNIP1, TOLLIP, TOP1, TPD52L2, TPI1, TPMT, TPP1, TRAF3, TRAF3IP2, TREML3P, TRIM33, TRIP10, TRNT1, TRPM2, TSHZ1, TXN2, UBE2V2, UBE3B, UBQLN2, UBR4, UEVLD, UPB1, USP9X, USPL1, UVRAG, VDAC1, VDR, VEGFA, VHL, VILL, VOPP1, VRK1, WASHC5, WDFY3, WDR45B, WI2-87327B8.2, WSB1, XPR1, ZBTB21, ZBTB43, ZC3H11A, ZDHHC20, ZEB1, ZEB2, ZFX, ZFYVE16, ZFYVE26, ZKSCAN1, ZMIZ1, ZNF100, ZNF24, ZNF292, ZNF433-AS1, ZNF512, ZNF710, ZSWIM6 |
| mediumpurple3 | ABHD11, ACOXL, AURKAIP1, BRD9, C6orf226, CABLES2, CAPN13, CD1D, CD4, CD99P1, CENPN, CHCHD3, CHPF2, CLASP2, CLPTM1, CMAS, CPM, CR1, CTRL, CYP1B1, DNAJC16, DNMBP, DOCK9, EFCAB14, ERCC8, FARSA, FLJ42393, FUBP3, GART, GPAA1, GTF2IRD2B, HSF1, IL4I1, IQSEC1, KCNMB1, KDM3B, LDHAL6A, LOC100130298, LOC102723582, LOC105372064, LOC105372653, LOC105373311, LOC105376384, LOC105378721, LOC107984551, LOC107986488, LOC112267872, LOC112268088, LOC112268148, LOC388282, LOC388572, LTB, LY6G5C, MAP3K2-DT, MAPK9, MGST1, MINDY4B, MIR6840, MMP24, MTHFSD, NEIL3, NHSL1, OVCH1-AS1, PECR, PGRMC1, PHF5A, PIANP, PIK3R4, PLAA, PLXNA2, POC5, POLR3E, PRIM2, PSMC3IP, RAB1B, RBL2, RNASEL, RNF115, SELENOW, SH3BP5L, SNAI3-AS1, SNORD13F, TALAM1, TBC1D22B, TCEAL4, TECR, TNFSF12, TRAF4, TRRAP, TTI2, USP12, WEE1, WIZ, XRCC4, ZNF408, ZNF547, ZNF671 |
| midnightblue | ACOT9, ADNP2, ADORA2A, ALAS1, ALOX12, ALOX5AP, AMDHD2, AMZ2P1, ANKRD33B, AREG, ARHGAP18, ARHGDIA, ARL5B, ARMCX3, ASPRV1, ATG2A, ATG7, ATP2B1, ATP2B1-AS1, ATP6V0C, B3GALNT2, B3GNT5, BABAM2-AS1, BAG3, BCAS2, BCL2A1, BFAR, BIRC3, BRAF, C10orf55, C15orf39, C18orf32, C1orf43, C2CD3, C5AR1, CACYBP, CAMK1G, CARD17, CARD19, CASTOR1, CCL3, CCNH, CCRL2, CCS, CD44, CD48, CD53, CD83, CEP170P1, CFAP58, CFAP58-DT, CHD2, CHMP2A, CLEC1B, CLEC4E, CLEC5A, COMMD5, CPD, CREM, CSRNP1, CXCL1, CXCL16, CXCL2, CXCL3, CXCL8, CYSTM1, DARS-AS1, DCUN1D3, DDIT3, DDIT4, DDX21, DENND4B, DNAJB1, DNAJC3-DT, DNAJC8, DNTTIP2, DUSP2, DYNLT3, EDN1, EGR3, EHD1, EHD4, ELL2, ELMO1, ETF1, ETS2, EXOSC4, FAM177A1, FAM177B, FAM210A, FCER1G, FGD4, FOSL2, FTH1, FTH1P3, G0S2, GABPB1, GADD45B, GARS1, GBP3, GGA1, GK, GLA, GLUL, GMEB1, GPR65, GSTO2, GTF2H1, HCAR2, HCAR3, HCG18, HIP1, HMOX1, HPS5, HSP90AB1, HSPA1B, HSPA7, HSPH1, ICAM1, ID2, IER5, IL10RB-DT, IL1A, IL1B, IL1R1, IL1RN, IPMK, IRAK2, IRF2BP2, ITPRIP, IVNS1ABP, JOSD1, KDM6B, KLF10, KPTN, LAMB3, LAMTOR1, LAMTOR3, LCP2, LDLR, LIF, LINC00847, LINC01366, LINC02649, LINC02656, LINC02728, LMNB1, LOC100128059, LOC100130357, LOC100287467, LOC101927851, LOC101929007, LOC101929319, LOC102723694, LOC105369402, LOC105371934, LOC105372652, LOC105375084, LOC105375378, LOC105375924, LOC105376504, LOC105376976, LOC105378083, LOC105378198, LOC107984034, LOC107984316, LOC107984485, LOC107984658, LOC107985455, LOC107986153, LOC107986160, LOC107986193, LOC107986664, LOC107987175, LOC112267953, LOC112267968, LOC112268118, LOC112694756, LOC388813, LOC653712, LSMEM1, MAFF, MAP1LC3A, MAP1LC3B, MAP1LC3B2, MAP2K3, MAP3K20-AS1, MAP3K8, MAPK6, MED28, MEI1, METTL6, MFSD2A, MINK1, MIR616, MORF4L2, MPP5, MXD1, NAMPT, NBN, NEDD9, NFE2L2, NFKB2, NFKBIA, NFKBID, NFKBIZ, NFXL1, NKX3-1, NOD2, NR1D1, NR4A3, NRIP3, NSMAF, OLR1, OSM, OXSR1, PAK1, PDE4B, PFDN1, PFKFB3, PHACTR1, PHLDA2, PIM3, PKN1, PLAU, PLAUR, PLEK, PLEKHM2, PLSCR1, PM20D2, PNP, PNPLA8, PNRC1, PPCDC, PPIF, PPP1R15A, PPP1R15B, PRDM8, PRKCH, PRKDC, PTGS2, PTPN1, RAB1A, RAB8B, RAB9A, RABGEF1, RALGAPA1, RANBP2, RBKS, RILPL2, RNASEK, RNF103-CHMP3, RNF167, RNF185, RNF41, RRAGC, SAMD4B, SAMSN1, SDCBP, SEC13, SEC22B, SEC62, SELENOK, SERP1, SFT2D1, SGK1, SH2D3A, SH3BP5, SKIL, SLC25A51, SLC2A3, SLC36A4, SLC3A2, SMIM29, SMU1, SMURF2, SOD2, SPAG9, SQSTM1, SRGN, STIP1, STX4, SUN2, SYAP1, SYTL3, TAGAP, TBC1D30, TBC1D7, TGIF1, THAP9-AS1, TMED10P1, TMEM231, TMEM52B, TNFAIP3, TNFAIP6, TNFRSF1B, TNFSF14, TOM1, TPM4, TREM1, TSC22D1, TXNDC11, UBB, UBBP4, USP36, VKORC1, VPS18, VPS26A, WTAP, XBP1, YIPF6, ZC3H12A, ZDHHC5, ZFAT, ZNF165, ZNF250, ZNF252P, ZNF267, ZNF277, ZNF43, ZNF654, ZNRD1, ZSWIM4 |
| navajowhite2 | AP3M2, CCDC138, CCN2, CDCA5, CMC4, CTSV, DENND5B-AS1, EPS15-AS1, EXD3, FAM161B, FASN, FHAD1, HM13-AS1, LIX1, LOC101928371, LOC105377584, LOC107984602, LOC171391, LOC644215, MIR573, NDUFAF5, NRN1L, PHLDB2, PTK2, RMRP, RPS14P3, SAMD12, SDR16C5, SERINC2, SSTR3, STC1, TMPRSS13, UBL7-AS1, XPNPEP1, ZNF585B |
| orange | ABCC5, ACOX3, ADNP, ALDH2, ALDH3B1, AMMECR1L, APBA3, APEX1, ARID3A, ATG10, ATG2B, ATP1B3, ATP2C1, BATF3, BBC3, BBS4, BEX4, BMX, BOLA3-AS1, C14orf28, C6orf62, CAND1, CAPRIN2, CCAR1, CDC14A, CDKN2AIP, CELF6, CERS4, CHCHD1, CHCHD10, CLASP1, CLEC18A, CLINT1, CMTM3, COASY, COL27A1, COPS2, COQ2, DAZAP1, DDX43, DDX52, DHX33, DMTF1, DNAJC3, DRICH1, EIF4B, EIF4ENIF1, EPOR, EXOSC9, FAAP20, FAM110B, FAM91A1, FANCA, FKBP1AP1, GABPB1-IT1, GATAD2A, GFM1, GNL3L, GOLPH3, GP1BA, GPANK1, GPD2, GPR19, GUSBP14, GUSBP15, HAUS8, HDAC2, HDAC4, HECTD1, HTATSF1, INHBB, INSR, INSYN2B, INTS9, IQGAP3, KBTBD4, KCNH7, KCTD2, KCTD21, LOC100129931, LOC100506585, LOC102723415, LOC105370461, LOC105370547, LOC105372578, LOC105372697, LOC105373133, LOC105374542, LOC105374845, LOC105375036, LOC105375536, LOC105375746, LOC107985403, LOC107985869, LOC107986077, LOC107986304, LOC107986668, LOC112268043, LOC112268063, LOC112268260, LOC647070, LRRC28, LRRC29, MAP3K2, MBTPS1, METTL26, MFSD8, MICALCL, MMP24OS, MMP9, MMUT, MTFR2, MTIF2, MTMR9, MTOR, MYBPH, NIPSNAP2, NKRF, NLRX1, NME7, NPAT, NPEPPSP1, NRDE2, NSA2, NT5DC3, NUDT1, OAT, OPA1, OXLD1, PCGF1, PDCD6IP, PDCL, PDE8A, PDHB, PHAX, PIGBOS1, PLGLB2, PLXNC1, POLDIP2, PPP1R3D, PPP2R2D, PRDM10, PSMD3, PTPN22, QTRT2, RAB11B-AS1, RAB11FIP2, RAB15, RABL2B, RETREG2, RGPD2, RGS16, RNF14, RNF19A, RPA1, RPRD2, SECISBP2, SHLD1, SLC17A5, SLC25A12, SMARCD2, SMC4, SMG1P5, SNAP29, SNRNP200, SNX16, SPRTN, SPTAN1, SRCAP, STAG3L5P-PVRIG2P-PILRB, STX8, TASOR2, TEDDM1, THAP12, TOMM20, TOP2B, TRIM52, TRMT10B, TTC19, UBE2G2, UBLCP1, USE1, VAMP1, VIRMA, VPS11, VPS13C, WDR73, YIPF5, YOD1, ZBED5, ZDHHC9, ZGPAT, ZNF136, ZNF319, ZNF44, ZNF518B, ZNF687-AS1, ZNF763, ZNF805, ZNF92 |
| palevioletred3 | ACTR8, ACYP1, ATN1, CEACAM6, DUS3L, ELF3-AS1, EPN2, FAM135A, GEMIN5, GRAMD1B, H2AC13, HDAC10, LOC101929650, LOC102723922, LOC105375218, LZTR1, MARC1, MCM3, MGARP, MRPS33, PARPBP, PIGR, PRDM5, PRRT1B, PSMG1, RCAN3, RMI2, SELENON, SSH3, STAR, TCF7, TLCD3A, TMEM106C, UBA5, VKORC1L1, ZNF137P |
| pink | ABHD2, ABHD3, ABTB1, ACAP2, ACOX1, ACPP, ACTR10, ADD3, AGER, AGFG1, AIM2, AIP, AKAP13, ALDH9A1, ALG6, ANAPC16, ANKRD28, ANKRD44, ANP32E, ANXA2, ANXA2P2, AOAH, AP2M1, APBB1IP, APH1B, API5, APOL2, AQR, ARGLU1, ARHGDIB, ARID1A, ARL11, ARL6IP5, ARL6IP6, ARPC2, ARPC3, ARRB1, ATF6, ATF7IP, ATG5, ATP5F1B, ATP5F1C, ATP5MD, ATP5PO, ATXN3, ATXN7, B3GAT2, B9D2, BAG4, BAZ1A, BAZ2B, BBX, BCLAF1, BLVRA, BMP2K, BOD1L1, BPTF, BRD3, BROX, BUB3, C11orf58, C11orf98, C18orf25, C1orf131, C1orf52, C2orf68, C2orf69, CALCOCO1, CAMK2G, CAMLG, CAPNS1, CARD8, CASP2, CASP8, CASS4, CBWD3, CBWD5, CBX3, CCDC28A, CCDC69, CCDC90B, CCND3, CCNDBP1, CCNJL, CCPG1, CD164, CD46, CD47, CDADC1, CDC123, CDC42SE2, CDK19, CDK9, CDS2, CDV3, CEBPB, CEBPD, CELF2, CEP350, CGGBP1, CHST11, CHTOP, CHURC1, CISD2, CISH, CKLF, CLEC1A, CLEC7A, CMC1, CNIH4, COLGALT1, COPG1, COPS9, COQ7, COTL1, COX7B, COX7C, CPPED1, CRBN, CRIPT, CRLF3, CTDSP2, CUEDC2, CUL3, CUTC, CWC15, CWF19L1, CXCR1, CXCR2, CYTH4, DCAF6, DCTN3, DDHD1, DDX59, DECR1, DGCR2, DHRS12, DICER1, DIPK2A, DIS3, DLEU2, DNMT3A, DOCK11, DOCK8, DPEP2, DPYD, DR1, EAPP, EBLN3P, EDEM3, EFCAB2, EIF3G, EIF3M, EIF4E2, EIF4E3, EIF5B, ELOF1, ELOVL5, EMC3, EMC7, ENTPD1, ERGIC2, EXOC6, FAM126B, FAM8A1, FBXL20, FBXO3, FBXO30, FBXO6, FCGR3A, FCGR3B, FCHO2, FCHSD1, FGFR1OP, FGL2, FKBP5, FKBP9, FMC1, FOS, FPGT, FRAT1, FRY, FYB1, G3BP1, GALK1, GCA, GDE1, GGNBP2, GID8, GIHCG, GLB1, GLIPR1, GMEB2, GOLGB1, GPR155, GSEC, GSK3B, GTF2B, GTF2E2, GTF2H2, GTF2H2B, GTF2H2C, GTF2IP4, H2BC12, H2BC5, HAUS4, HDAC1, HDLBP, HEATR5A, HERC3, HIGD2A, HIPK1, HIPK3, HLA-A, HNRNPUL1, HNRNPUL2, HP1BP3, HSD17B11, HSDL2, HVCN1, IDH3B, IDI1, IFI16, IGBP1, IKBIP, IKZF1, IL13RA1, IL16, INKA2, INPP5D, IQGAP1, IQGAP2, IRF2, ITGAE, ITGB2, ITPRID2, ITSN2, JADE1, JAML, JDP2, JKAMP, KAT2B, KAT6A, KIAA0040, KIAA0232, KIAA1109, KIAA1257, KIF13A, KIF5B, KLF3, KLHL5, KLHL8, LAPTM4A, LBR, LIMD2, LIN7A, LINC02289, LMBRD1, LOC100129034, LOC100507006, LOC101928674, LOC101929240, LOC102723340, LOC105370135, LOC105373711, LOC105374407, LOC105375945, LOC105376568, LOC105378415, LOC107984104, LOC107984788, LOC107984984, LOC107985409, LOC606724, LOC643802, LOC728392, LONRF1, LPAR2, LPCAT2, LPGAT1, LPIN1, LRMP, LRP10, LRRFIP1, LRRFIP2, LRRK2, LRRK2-DT, LSM12, LTBR, LYPLAL1, LZIC, MACF1, MAK, MAN1A1, MAN2B2, MANF, MAP2K6, MAP3K1, MAP3K5, MAPK14, MAPK3, MAPKAPK3, MARCHF2, MAT2B, MBD6, MBNL1, MBOAT2, MED13, MED4, MED8, MEGF9, MESD, MIDN, MIR320E, MKRN1, MLKL, MMADHC, MNDA, MNT, MOB1A, MON1B, MORF4L1, MPHOSPH8, MPLKIP, MPPE1, MPZL1, MRPL20, MRPL22, MRVI1, MSL1, MSL3, MSRB1, MTFMT, MYADM, MYCBP2, MYO15B, MYO1F, NAIP, NANS, NARF, NCF4, NCKAP1L, NCL, NCOA1, NCOA6, NDUFA10, NDUFB10, NDUFB4, NDUFB8, NDUFC2, NFE2, NKTR, NOSIP, NR3C1, NRBF2, NSD1, NUDC, NUDT5, NUP214, NUP50, OCIAD1, OGFRL1, OGT, OSBPL1A, PACS1, PAFAH1B2, PARK7, PARP9, PCBP2, PCED1B-AS1, PDCD5, PDK3, PDS5B, PECAM1, PER2, PET100, PFAS, PFDN2, PGD, PGGT1B, PHC2, PHOSPHO1, PIGB, PIP4K2A, PIP4P2, PITPNB, PJA2, PKN2, PLBD1, PLCB2, PLCL2, PLEKHO1, PLIN5, PLP2, PLXDC2, PMF1, PNISR, POLR2B, POLR2C, POLR2D, POLR2G, PPCS, PPIA, PPIG, PPIP5K2, PPP1R12A, PPP1R7, PPP2R2A, PPP2R5E, PPP6R1, PRAM1, PRDX3, PRELID3B, PRPF38B, PRPF40A, PRPS2, PSIP1, PSMA2, PSMA4, PSMB1, PSMB4, PSMC1, PSMD12, PSMD7, PSMD9, PSME3, PSMF1, PTDSS1, QRICH1, RAB11FIP1, RAB18, RAB27A, RAB29, RAB37, RAB3D, RALA, RAP1GAP2, RBBP6, RBM8A, RBPJ, RBX1, RCOR3, RDH10, REEP5, RELL1, RERE, REST, REV3L, RFX3, RFXANK, RGL2, RGL4, RGS18, RIPOR2, RNASET2, RNF111, RNF122, RNF130, RNF24, ROCK2, ROMO1, RPL11, RPL13A, RPL26L1, RPL27, RPL28, RPL3, RPL30, RPL31, RPL35, RPL37A, RPL5, RPS14, RPS16P5, RPS27, RPS3A, RPS4X, RPS6KA1, RPS6KA5, RRM2B, RSBN1L, RSL24D1, RUNX2, SAFB2, SARAF, SARS1, SCAF11, SCARNA22, SDCBP2-AS1, SDHAP2, SEC61B, SELENOF, SELPLG, SEMA4B, SGF29, SKP1, SLC25A44, SLC37A3, SLC39A9, SLC40A1, SLC8A1, SLF1, SLIRP, SMAGP, SMAP2, SMARCA2, SMC3, SMCO4, SNAP23, SNORA77, SNRPG, SNX6, SOD1, SORL1, SP110, SPEN, SPG21, SPOP, SPOPL, SRGAP2C, SRP19, SRP9, SRSF1, SRSF4, SSH2, SSR1, SSR4, SSU72, ST8SIA4, STIM2, STK17B, STRN3, STX10, STX7, SUPT4H1, SURF1, SYK, TAFA2, TALDO1, TBC1D10C, TDG, TENT2, TENT4B, TGOLN2, THOC5, TIMP2, TK2, TKT, TLR1, TLR5, TLR6, TLR8, TM2D1, TM9SF2, TMBIM4, TMC6, TMED2, TMEM11, TMEM184B, TMEM208, TMEM260, TMEM33, TMEM59, TMLHE, TNFRSF10C, TNFSF10, TNPO1, TNRC6B, TOMM7, TOPBP1, TOX4, TRA2B, TRAPPC2L, TRERF1, TRIM5, TRIP4, TRPM6, TSEN34, TSNAX, TUBA1A, TUBA1B, TUT7, TXNDC12, UBE2CP5, UBE2D3, UBE2E3, UBE2I, UBE3A, UBE4A, UBR2, UFC1, UFD1, UGP2, UIMC1, UMAD1, UQCRB, USP1, USP15, USP3, USP7, UTP23, VAMP8, VBP1, VDAC3, VPS9D1, VTI1B, WAS, WNK1, XRN2, XYLT2, YIPF4, YKT6, YPEL3, ZBTB11, ZBTB44, ZC3H15, ZFAND2B, ZMPSTE24, ZNF101, ZNF106, ZNF276, ZNF33A, ZNF467, ZNF592, ZNF652, ZNF830 |
| plum2 | ADAP1, ARHGAP27P1, ARPC5L, ASAP1-IT1, BCR, BTF3, CA5BP1, CD300E, CEP120, CPNE8, CPSF6, DNAH1, DTD1, FAM149B1, FAM20A, GNL2, GPNMB, GTF3C1, H1-2, HUS1, IGFBP7, KLHL2, MGAT4A, MIR4477B, MRPS11, MSH3, MYL12B, NAA60, NBPF9, NFYB, NME8, NMT1, PDE4A, PIGT, PTTG1, RABEPK, RAD23B, RAVER1, RBIS, RBM6, REXO2, RPL22, RPL39, RPS29, RTL10, SIKE1, SMARCE1, TAF3, TCP11L1, TLR10, TPP2, TTLL12, UBE2J2, ZNF780B, ZSWIM1 |
| purple | AAMP, AATF, ABCA11P, ABCA2, ACBD5, ACOT13, ACTN4, ADGRE5, AFMID, ALOX5, AMD1, ANAPC5, ANKS1A, AP5M1, APOBEC3C, APOBR, APOL1, ARF6, ARHGAP1, ARHGAP25, ARHGAP30, ARMC8, ARSG, ASB8, ASNSD1, ATF2, ATG16L2, ATM, ATP11C, ATP5PD, ATP5PF, ATXN1-AS1, ATXN7L3B, BANF1, BCCIP, BCKDHA, BCL2L12, BIN2, BIN3-IT1, BIRC6, BLVRB, BMT2, BNIP2, BOLA2-SMG1P6, BORCS7, BRWD1, BRWD3, BTN3A3, BUD23, C1GALT1C1, C21orf91, C4orf46, C6orf120, C9orf16, C9orf78, CACUL1, CALHM6, CAMK1D, CANT1, CARD8-AS1, CBL, CBLL1, CBX3P2, CBX7, CCDC126, CCDC17, CCNK, CCT4, CCT5, CCT6A, CCT6P3, CCT8, CD300LF, CD9, CDA, CDK17, CEACAM21, CEBPZ, CENPC, CERS5, CHD9, CHMP4A, CHP1, CLIP1, CLMN, CLN5, CLP1, CMPK1, CNN2, CNOT10, CNTD1, CNTNAP3, CNTNAP3P2, CNTRL, COA6, COG1, COL18A1, COMMD1, COMMD3, COPS3, COPS4, COX4I1, CPEB3, CPT1A, CRCP, CRK, CRYBG1, CSAD, CSRNP2, CST7, CTNNAL1, CXorf56, CYBC1, CYBRD1, CYTH2, DAP3, DCP2, DDB1, DDOST, DDX42, DGKA, DGLUCY, DHDDS, DHPS, DHRS13, DHRS7, DHRS7B, DHTKD1, DHX36, DKFZP586I1420, DLD, DMXL1, DNAJC13, DNASE2, DOK4, DOP1B, DPF2, ECH1, EDF1, EIF2S1, EIF3K, ELMO2, ELP1, ENSA, EP300, ERG28, ERO1B, ERP29, FABP5, FAM110A, FAM120AOS, FAM214A, FAM53A, FAM53C, FAM89B, FBRSL1, FBXO33, FBXO7, FBXW11, FCGR2A, FDFT1, FFAR2, FLJ32255, FOXP1, FRMD8, FRY-AS1, FTSJ3, FUNDC1, FYTTD1, GABARAPL1, GALK2, GALNT11, GANAB, GBF1, GBP2, GCM1, GFOD2, GGPS1, GLE1, GLIPR2, GMCL1, GMDS, GNAI2, GNAT2, GPKOW, GRB10, GRK6, GTF2F2, GTF3A, H2AJ, HADHA, HARS1, HCCS, HCG26, HEATR5B, HECW2, HELZ, HERC6, HIGD1A, HK1, HK3, HNRNPAB, HNRNPK, HSBP1L1, HSP90B1, IDH3G, IFNAR1, IL18, IL3RA, ILF2, ILF3-DT, IMPA2, INAFM1, INPP5B, INTS11, IPO8, IPP, IRAK4, ISG20L2, ISOC1, ITFG1, ITGAD, IWS1, JAZF1, KAZN, KIAA0513, KIAA1324, KIF1C, KIF21B, KLHL15, KLHL7, KMT2E-AS1, LAIR1, LAP3, LATS1, LINC00260, LINC00309, LINC00467, LINC00921, LINC01146, LINC01215, LINC01531, LINC02362, LINC02471, LINC02555, LINC02751, LINS1, LMAN2, LOC101927069, LOC101928093, LOC101928429, LOC101929667, LOC102724389, LOC105369380, LOC105369748, LOC105371240, LOC105372261, LOC105372657, LOC105373148, LOC105374071, LOC105375547, LOC105377449, LOC105378841, LOC107984036, LOC107984421, LOC107984669, LOC107984974, LOC107985209, LOC112267877, LOC112268067, LOC441601, LOC731075, LPCAT3, LRRC57, LSM7, MAF1, MAL, MALT1, MAN2A2, MAP2K2, MAP3K7, MAU2, MAX, MCM3AP, MCMBP, MDH1, MEA1, MED12, MFSD13A, MGAM, MGAM2, MGAT2, MGST3, MICB-DT, MIR3128, MMGT1, MOSPD2, MRGBP, MRPL21, MRPL28, MRPL36, MRPL44, MRPL54, MRPS16, MRPS21, MRPS28, MTFR1L, MXI1, NAA50, NAAA, NACA, NAGK, NCCRP1, NCF1C, NCK2, NDST2, NDUFAB1, NDUFB11, NDUFB7, NDUFS5, NDUFS6, NDUFS7, NFAM1, NIPBL-DT, NIPSNAP1, NIT1, NLK, NMI, NPRL2, NSUN3, NSUN7, NT5C3A, NUDT16L1, NUDT21, NUP155, NXPE3, OAZ1, OCIAD2, OIP5-AS1, ORMDL1, OSGEP, OTUD6B-AS1, PADI4, PARL, PCNX1, PCNX3, PDCD4, PDE6D, PDLIM2, PDS5A, PEX2, PFKFB2, PGPEP1, PHC3, PHPT1, PIAS2, PIGH, PIK3R1, PITHD1, PITPNC1, PLEKHM1P1, PLGRKT, PNPLA2, POC1B, POLE4, POLR2F, POLR3A, POLR3K, PPIL4, PPP1CC, PPP1R12B, PPP1R8, PPP1R9B, PPP2CB, PPP4R3A, PRCC, PRDX4, PRMT5, PRMT9, PRPF39, PRR14L, PRSS8, PSMB6, PSMC3, PSMD2, PTBP3, R3HDM4, RAB11B, RAB11FIP4, RAB3IP, RASSF1, RBM10, RBM22, RBM27, RBMX, RBMX2, RDX, RENBP, RGS3, RIDA, RIF1, RIN2, RNF135, RNF138, RNF168, RNF31, RNF6, RPAP3, RPL14, RPN1, RPS17, RPS2, RPS23, RPS6KB2, RPSA, RPSAP58, RSPH9, RWDD4, S100A10, SACM1L, SARNP, SBF1, SCAF1, SDF2L1, SEC22A, SENCR, SEPTIN7, SESTD1, SET, SH3BGRL3, SKA2, SLC19A1, SLC25A45, SLC2A6, SLC30A6, SLC30A9, SLC35B1, SLC35B3, SLC36A1, SLC39A4, SLC44A1, SMIM8, SMPDL3A, SNHG3, SNX1, SOS2, SPAST, SPATA2L, SPATA5L1, SPCS2, SPDYE2, SPRYD3, SRI, SSBP1, SSBP3, SSR3, ST20-MTHFS, STAT1, STAT6, STN1, STOM, STOML2, STX16-NPEPL1, SUMF1, SUMO4, SURF4, SUZ12P1, SVIL-AS1, SYTL1, SZT2, TADA2B, TAF2, TAOK3, TBPL1, TBXAS1, TCP11L2, TERF1, THAP2, THUMPD1, TIA1, TIAM2, TIGD3, TJP2, TK1, TMEM101, TMEM127, TMEM167A, TMEM168, TMEM170A, TMEM170B, TMEM179B, TMEM216, TMEM234, TMEM259, TMEM273, TMEM69, TMSB10, TMX4, TNFRSF12A, TNRC6C, TP53I11, TPRKB, TRAPPC12, TRAPPC5, TRIM28, TRIQK, TRMT10C, TSPAN3, TSPO, TSTD1, TTC22, TUBB4B, TUBGCP3, TUFT1, TYW1B, UBE3C, UBTD1, UCP2, USP49, VAMP7, VCL, VCP, VNN3, VPS26B, VPS39, VRK2, WAC-AS1, WASH3P, WDR1, WDR44, WIPI2, WLS, WWC3, WWP2, ZBTB7B, ZCCHC7, ZCRB1, ZDHHC7, ZFP36L1, ZFPL1, ZFR, ZNF18, ZNF354A, ZNF430, ZNF562, ZNF608, ZNF674-AS1, ZNF814, ZNF818P, ZSCAN16, ZSWIM8, ZXDC |
| royalblue | ABCA13, ABCA5, ACTR6, ADSL, AGA, AHSA2P, AKAP11, AKNA, ALDH16A1, ALKBH1, ANKRD19P, ANKRD50, ANP32A, ANXA9, AOPEP, APOLD1, ARF4-AS1, ARHGAP12, ARID3B, ARL17A, ASB7, ATG4A, ATP8B4, B3GALT2, BAK1, BCDIN3D, BLOC1S4, BRAT1, BRI3BP, BRPF1, C16orf46, C1orf174, C1R, C4orf47, C7orf25, CAMKK1, CCDC112, CCDC12, CCDC191, CCDC43, CCNQ, CCSAP, CD36, CDC27, CDK12, CDK13, CEP295, CIRBP, CLEC2D, CNOT6L, COMMD10, COX11, CPSF1, CSNK2A1, CSNK2A3, CTDSPL2, CUL1, DCUN1D1, DEAF1, DPM2, EBAG9, ECPAS, EEF1AKMT2, EFHC1, EFNA4, EFNA5, EIF2AK1, EIF2B2, ELK4, ELOA, ELP5, EMC8, ERCC6, ETFDH, FAM184B, FLJ45513, FOPNL, FUCA1, GKAP1, GLT8D1, GLUD1, GNL1, GTF2IP1, GTF3C2, GXYLT1, H3P6, HCG27, HEXIM1, HNRNPA0, HS2ST1, IARS2, ICA1, IDI2-AS1, IRF2BPL, ITGB3BP, ITPR1-DT, KCNJ13, KCNJ2, KCTD18, KDM4A, KIF27, KLF4, KMT5B, LARP1, LILRA1, LIN52, LINC00672, LOC100506100, LOC100506235, LOC100506639, LOC101927556, LOC101927974, LOC101928304, LOC101928893, LOC101930112, LOC102723704, LOC105369663, LOC105370191, LOC105370491, LOC105370557, LOC105373637, LOC105374664, LOC105376220, LOC105377645, LOC105378041, LOC105378723, LOC105379086, LOC105379173, LOC107983998, LOC107984092, LOC107984988, LOC107985360, LOC107987072, LOC112267891, LSM14B, LYRM4, MADCAM1, MANBAL, MAP4K2, MAPKAPK5, MED6, MFSD5, MIS12, MIS18BP1, MRPL3, MRPS5, MRPS9, MT1G, MTERF2, MTM1, MZT1, NFATC1, NFATC2IP, NFS1, NSUN5, NUP43, OFD1, OSBPL8, OXER1, OXNAD1, PARG, PCMTD1, PDXDC2P-NPIPB14P, PEAK3, PEX14, PHB2, PIP5K1B, PLA2G7, PPAT, PPM1B, PRKAG2, PRKCB, PRUNE1, PYROXD2, R3HCC1, RAMAC, RASSF2, RCSD1, RECQL, RFLNB, RNF125, RPL23AP7, RPL6, RPS6KC1, RREB1, RUNDC3B, SAP30L, SART3, SASS6, SCP2, SCYL3, SEMA3F-AS1, SETD2, SFMBT1, SFXN5, SLC25A39, SLC26A6, SLC35A3, SMAD4, SND1, SNHG10, SNORA25, SNORA32, SNORD6, SNRPC, SNX4, SOCS4, SRRD, SSPN, STAM2, STUB1, SUFU, SYCP2, TAB3, TARM1, TBKBP1, THBS1, THRA, THRAP3, TM7SF3, TMEM106B, TMEM250, TMEM265, TMEM9, TP53INP1, TRAPPC11, TRIM11, TSPOAP1-AS1, TTC26, UBASH3B, UBE2O, UCHL5, USP54, VWF, WRAP73, ZBTB33, ZHX1, ZNF217, ZNF236, ZNF37A, ZNF468, ZNF586, ZNF611, ZNF615, ZRSR2, ZSCAN26 |
| salmon | ABCC6P2, ABCF1, ACKR3, ADAMTSL5, ADGRG6, AFF1-AS1, AGBL2, AGBL5, AGR2, AHNAK, AIFM2, AK1, ALG1L2, AMOTL2, ANKRD1, ANKRD18A, ANKRD46, APH1A, ARHGEF16, ARHGEF35-AS1, ARHGEF38, ARMCX2, AS-PTPRE, ASCL1, ASMT, ASNS, ATIC, B4GALT6, BARX2, BBOF1, BDH1, BICDL1, BMPR1B-DT, BPIFA1, BPIFB1, BUB1, C16orf74, C17orf67, C1QL3, C1QTNF6, C2CD2, C2orf42, C5orf38, CACNA1A, CACNA2D4, CACNG1, CALHM5, CAMK2N1, CAMK4, CAMTA1-DT, CAV2, CBS, CCDC189, CCDC68, CCDC96, CCNA1, CCSER1, CD70, CDC37L1-DT, CDC42BPB, CDCA7, CDH11, CDKN2C, CEP57L1, CEP70, CEP76, CFB, CFH, CHKB-DT, CKMT2-AS1, CLUH, CPEB2-DT, CPSF2, CRYZ, CST1, CXCL10, CYP26B1, CYP2S1, DBF4B, DBR1, DCN, DGKD, DHCR24, DLAT, DNAH7, DOCK6, DPP4, DSC3, DUOXA1, DUSP4, ECI2, ECSIT, ENAH, EREG, EXOC6B, FAIM, FAM131B, FAM30A, FAM3D, FAM53B, FAM72C, FAM83H, FAM86B1, FAM86B2, FBXL6, FBXO36, FGF11, FGFR3, FGFR4, FHAD1-AS1, FIGNL1, FILIP1, FLNC, FPGS, FRMD7, FRZB, FSTL1, GALNT5, GBX2, GEMIN2, GINS2, GJC2, GLCCI1, GLTPD2, GLYATL2, GOLGA6C, GOLPH3L, GOT2, GPD1L, GPR161, GPR89A, GRB7, GRHL2, GRPEL2-AS1, GTF2IP7, HAGHL, HNRNPA3P1, HOXA1, HOXA5, HOXB-AS3, ID4, IFNK, IFT22, IGBP1P1, IGFBP2, IGFBP3, IGFBP4, IGFBP6, ILDR1, IMMP2L, INTS5, IQSEC2, IRGC, ITGA2, ITGA3, JCAD, KBTBD6, KCNG2, KCNJ5, KCNJ6, KCNK7, KIAA1614-AS1, KIR2DL5A, KIR2DS4, KLHL26, KLK5, KRT81, KRT86, LAMB2, LARGE1, LGALSL-DT, LINC00293, LINC00444, LINC00537, LINC00592, LINC00621, LINC00673, LINC00685, LINC00945, LINC01315, LINC01355, LINC01530, LINC01620, LINC02088, LINC02227, LINC02265, LINC02518, LINC02560, LINC02596, LINC02851, LINCR-0001, LIPC, LOC100129534, LOC100310756, LOC100505715, LOC100506473, LOC100507634, LOC100996419, LOC101927490, LOC101927752, LOC101928007, LOC101928075, LOC101928530, LOC101928817, LOC101929018, LOC101929142, LOC101929977, LOC101930129, LOC102546294, LOC102723382, LOC102723649, LOC102723672, LOC102724832, LOC102725254, LOC105369645, LOC105370194, LOC105370205, LOC105370460, LOC105370492, LOC105371058, LOC105371081, LOC105371268, LOC105371942, LOC105372212, LOC105373174, LOC105374897, LOC105374944, LOC105375322, LOC105375543, LOC105375704, LOC105375724, LOC105376360, LOC105376361, LOC105376878, LOC105377157, LOC105377308, LOC105377623, LOC105377951, LOC105378199, LOC105378539, LOC105378807, LOC105379163, LOC107984023, LOC107984543, LOC107985012, LOC107986172, LOC107986552, LOC107986629, LOC107986634, LOC107986714, LOC107986847, LOC107986910, LOC107986949, LOC107987100, LOC107987171, LOC112268225, LOC112268334, LOC403312, LOXL2, LRRC1, LRRC46, LY6G6C, LZTS2, MAGED1, MAPK10, MAPT, MARS2, MATN1, MECOM, MEIS1, MGP, MIR16-1, MIR181A2HG, MIR205HG, MIR26B, MIR3611, MIR3649, MIR423, MIR548P, MIR6748, MIR6838, MLST8, MMP7, MRGPRE, MRPL9, MSL3P1, MTA3, MTRF1L, MUC1, MUC16, MUCL1, MYZAP, NCAM2, NECTIN3, NEDD4L, NODAL, NOXA1, NPNT, NR2E1, NT5DC2, NT5E, NUP205, NYAP2, OGDHL, OR2W5, PADI6, PARP2, PBK, PDC, PDE4C, PDK2, PFKP, PGAP3, PGLYRP4, PHF14, PLAC9, PLEKHA5, PLEKHA8, PLPP2, PLS1, PLSCR4, PLXNB2, PMEPA1, PPP1R13B, PPP1R27, PPP2R5D, PRIM1, PRR15, PRR29, PRSS58, PSAT1, PSMG3-AS1, PTH2R, PUDP, PXDNL, PYCR1, RAB17, RARRES2, RASSF8, RBM14-RBM4, RBM44, RHBDL3, RHPN2, RIC1, RING1, RNASEK-C17orf49, RNF139-AS1, RNF182, RNVU1-6, RPL13AP20, RWDD2B, SAG, SCAND2P, SCARNA12, SCGB1D2, SCGB2A1, SCN2B, SCRN2, SDC1, SDHA, SERPINA12, SETD1A, SHROOM2, SIX1, SKA3, SLC12A5-AS1, SLC18B1, SLC1A1, SLC25A32, SLC39A2, SLC45A3, SMIM1, SNHG7, SNORD104, SNORD18C, SNORD35A, SNORD97, SOX17, SPAG16, SPANXC, SPDYE3, SPNS2, SRD5A2, SRM, SSX2IP, ST6GALNAC4, STC2, STRA6, SUN1, SYCE1L, TAS2R39, TCTEX1D4, TEK, TESK2, TESPA1, THAP6, THSD4, THUMPD2, THUMPD3, TIMM9, TIMP3, TIPARP-AS1, TJP1, TLR8-AS1, TM4SF1, TMEM150A, TMEM267, TMPRSS11A, TMPRSS9, TNS4, TOMM40, TP53I13, TRAPPC2B, TRERNA1, TRIM29, TRIM34, TRIM7, TRIP13, TRMT5, TSPAN1, TSPAN12, TSPAN9, TST, TTC12, TTF2, UCP1, UTS2, VSTM2L, VWDE, WDR54, WDR78, XKR6, XPNPEP2, ZNF132, ZNF204P, ZNF260, ZNF300, ZNF331, ZNF33B, ZNF343, ZNF391, ZNF418, ZNF496, ZNF548, ZNF551, ZNF71, ZNF831, ZSWIM9 |
| sienna3 | AGO1, ARAP2, ARAP3, ARHGAP9, ARHGEF3, BANP, BLOC1S6, BRD7, CALCOCO2, CBWD6, CDKL5, CEP135, CEP164, CFAP20, CHCHD5, CHKA, CLEC17A, CLTB, CMAHP, CNIH1, COPS6, CSDE1, DCAF8, DDX24, DDX5, DHRS9, DIP2A, DUSP11, EDRF1, EMC4, ERCC6L2, FAM126A, FAM27B, FMR1, GNL3, GOLGA1, GTF2H5, GTF2IP12, GTPBP10, HMG20B, HSPBAP1, IDO1, IFT52, INCENP, ITGB1BP1, KLHL6-AS1, LACTB, LDHB, LINC01094, LOC101928236, LOC105371981, LOC105374162, LOC105375457, LOC107984992, LOC107985303, LRRC4, LRRC59, LSM8, LUC7L2, LYSMD1, MANBA, MCM9, MED23, MGST2, MIOS, MLLT10, MRVI1-AS1, NCK1, NDUFA6, NTNG2, NUDT9, NUTM2A-AS1, PAQR3, PBRM1, PDE1B, PHACTR2, PIGC, PLPP5, PSMB5, PSMD11, RAF1, RAN, RBM15, RBM39, RCE1, RNF123, RNF152, RNF2, RNPC3, RUBCN, SCUBE3, SEC61A2, SERINC5, SF3B2, SLC9A3R1, SLK, SMCHD1, SNAPIN, SNORA78, SNTB2, SPAG1, ST6GALNAC2, STRADA, SUMO1P1, SYVN1, TAF7, TAS2R40, TM6SF1, TMED5, TMED7, TMEM126B, TMEM154, TMEM87A, TNKS, TNPO3, TOMM6, TRIM23, TTC14, UBAC2, UBE2A, UBL7, UBXN2B, USP16, USP39, VMP1, VPS28, WDR45, XKR8, ZMYM2, ZNF37BP, ZNF638, ZNF808 |
| skyblue | ABHD12B, ACADSB, AIDA, ANKRD13C, ARHGAP19, ARL14EP, ATP6V0A2, ATP8A1, ATR, B3GNT2, B4GALT3, BIK, C15orf40, C1orf50, CASK, CCZ1P-OR7E38P, CD302, CDK2AP1, CDKN1B, CDKN1C, CLDN10, CNNM3, CNOT6, CNTLN, COG6, CRISP3, CYP2R1, CZIB, DMAC1, DUSP13, EFCAB11, EHBP1, EMC10, EP400, EPAS1, EPHB4, EPHB6, EXOSC6, EXT2, F8A1, FAM157C, FAM200A, FAM234A, FAM3B, FAM78A, FASTKD5, FBXL17, FCMR, FLAD1, FLJ20021, FLYWCH2, FOXK1, FOXO1, FOXRED1, GAB1, GCDH, GFPT1, GGA2, GGH, GLUD1P3, GNB1L, GPALPP1, GTF2H2C_2, GTF3C5, HAUS6, HIRA, KANTR, KCTD10, KIAA2012, KIF13B, KPNA2, KRR1, LDLRAD4, LINC00893, LINC00937, LINC01409, LMO4, LOC105369869, LOC105378968, LOC105379251, LOC107985335, LOC107985522, LOC107985747, LOC107985949, LOC107986385, LOC107986926, LOC107987303, LOC112268426, LOH12CR2, LPCAT4, LRRC37A4P, LSM6, LY75, MAGOHB, MAVS, MCAT, MFN2, MFNG, MIB2, MICA-AS1, MIR548O, MORF4L2-AS1, MPND, MSRB2, MTERF1, MTURN, MYL5, NAA16, NECTIN1, NEDD8-MDP1, NRN1, NUP160, NUP54, NXT2, OCEL1, OTX1, PANX2, PELP1, PHOSPHO2, PIGN, PLCL1, PLOD3, PMF1-BGLAP, POC1A, PPP2R5A, PRKAG3, PYGM, R3HDM1, RALGPS1, RBM14, RBM33, RCAN1, REEP4, RHBDD1, RN7SL832P, RNF170, RPL21P44, RTN1, S100Z, SDHAF4, SGMS1-AS1, SH3GLB2, SIPA1L3, SLC12A4, SLC35E2B, SLC4A7, SLCO4C1, SNORD140, SPATA6, SPN, SPRYD4, ST14, STING1, STK25, SWI5, TASOR, TBC1D17, TCF7L1, TESC, TGFA, TMEM38A, TNRC6A, TPCN1, TRAF3IP1, TSPYL4, UBP1, VMA21, WDPCP, WDR61, XPO4, ZC3H6, ZKSCAN5, ZNF211, ZNF264, ZNF347, ZNF486, ZNF490, ZNF511, ZNF672, ZNF699, ZNF780A, ZNHIT3, ZSCAN32 |
| skyblue3 | ACAT1, ADAR, ALDH1A3, ANXA1, ANXA2R, ARFGAP2, ARHGAP4, ARL2BP, ATP1A1-AS1, B4GALT1-AS1, BICDL2, C16orf91, CCT7, CEACAM1, COQ8B, CPNE2, DCAF15, DCAF4, DUSP23, EDEM2, EHMT1, EPS8L2, EZH1, FAM157B, FGD6, GAS7, GBGT1, GEMIN7, GLO1, GMPPA, GTSF1, HEBP1, HEPH, HSD17B7, HSPB8, IDH1, IL20RB, INSL3, INTS10, INTS4, ITGA4, ITPRIPL2, KDM1B, KLK12, KRT18, KRT4, KRT6A, LIMA1, LINC02035, LINC02352, LOC101926887, LOC105370969, LOC105372295, LOC105374902, LOC105377016, LOC105379099, LOC105379752, LOC107984696, LOC644936, LRRC75B, MAIP1, MDM1, MIR4434, MLX, MRPL57, MTDH, MTVR2, NECTIN2, NRM, NUP133, PABPC1L, PI4K2A, PIEZO1, PNMA1, RABL6, RCN2, RNASE3, SASH1, SCAMP1, SCLT1, SCO2, SEPSECS-AS1, SERPIND1, SERPINH1, SHKBP1, SLC25A28, SLFN12, SMARCA4, SNRPF, SPINT1-AS1, STK11, STXBP5-AS1, SWT1, TBC1D2B, TM2D2, TNFAIP8L2-SCNM1, TRAM1, TRAPPC9, TRMT12, TRMT2A, TSPAN17, TUBGCP6, WASF2, WASH7P, WFDC21P, YIF1B, YTHDF3-AS1, ZNF117, ZNF609 |
| steelblue | ABCC6, ABCF2, ADCY10P1, ALG11, ANKAR, AP5S1, ASB13, ATE1, BAIAP2-DT, BFSP2-AS1, BTRC, C17orf80, C5orf66, C6orf47, CAD, CATSPER2P1, CCDC127, CCDC22, CD151, CD2BP2-DT, CDAN1, CDK8, CFAP298, CHCHD6, CLSTN1, CRLS1, CTSG, DAAM2, DANCR, DDB2, DDX54, DNAAF4, DYRK1B, EFCAB12, EIF2AK3-DT, EVL, EXOSC10, FAM223B, FGD2, FOXO4, GAL3ST4, GBAT2, GPATCH11, GPRIN3, GPS1, GPSM2, GRHPR, HAUS1, HBG2, HDAC6, HLA-DMB, HLA-DPB1, HYAL1, IL15, IL5RA, JADE2, JCHAIN, KIF14, KIF16B, KIT, KLHDC1, KLHDC10, LINC01545, LOC100286925, LOC100505774, LOC101926933, LOC101927151, LOC101927837, LOC101928004, LOC101929418, LOC102724532, LOC102724992, LOC105369655, LOC105369851, LOC105370359, LOC105371520, LOC105372279, LOC105372787, LOC105375521, LOC105377015, LOC105377469, LOC105378120, LOC107984835, LOC107986087, LOC107986514, LOC107986811, LOC648987, LRSAM1, LYVE1, MCM8, MED22, MFAP3L, MGAT5, MICALL1, MIR1244-3, MIR373, MIR4755, MIR5690, MPST, MTRNR2L10, MYCBP, MZT2A, NOMO2, NRADDP, NSMCE4A, NT5DC1, NUP88, OTUD6B, P2RY8, PAFAH1B3, PARD6A, PAXIP1, PCLAF, PCSK7, PDE6B, PDE8B, PDYN, PEF1-AS1, PHF24, PKD2, PMS1, POLR2H, PPIH, PPP1R12A-AS1, PTPN4, RB1-DT, RBAK, RGCC, RIOK2, RMI1, RRP36, SCCPDH, SDSL, SELP, SEMA3C, SHLD2, SHLD2P1, SHTN1, SLC12A2, SLC16A4, SMYD2, SOCS6, SRD5A1, ST3GAL5, STAB1, STIMATE, STK32C, STRBP, SUOX, TBCE, TCTEX1D2, TESMIN, TFB1M, TSPEAR-AS1, TTC7B, TUBE1, TYW3, WASL, YEATS4, ZBTB3, ZFP69B, ZNF141, ZNF280D, ZNF302, ZNF419, ZNF639, ZNF718, ZNF782, ZNF862, ZNF93 |
| tan | ABR, ACAD10, ACTR3B, ADAM22, ADCY3, AHSA1, AIG1, AK6, ALB, ALDH1A2, AMY2A, ANKHD1, ANP32AP1, AP1S3, AP5Z1, ARFIP1, ARHGEF26, ARL13B, ASPSCR1, ATG14, ATP6V0E1, ATP6V1E1, ATP6V1G1, B2M, BAMBI, BEND7, BET1L, BLACE, BMERB1, BST1, BTNL3, C11orf80, C12orf49, C17orf107, C19orf71, C2orf15, C2orf80, C5orf51, C7orf26, C7orf50, CAAP1, CASP6, CAST, CBR3-AS1, CCDC61, CCL4, CCL4L1, CCL4L2, CCP110, CD1B, CD2AP, CD300C, CDC42BPG, CDC6, CDIP1, CDK2, CDYL2, CEACAM4, CEP72, CFAP36, CHD5, CHI3L2, CLEC6A, CLIP2, CLPTM1L, CNGA1, CPEB1-AS1, CREBRF, CTSZ, CUTA, CXCL6, CYREN, DBP, DCTN6, DDX60L, DHFR, DHX58, DIABLO, DNAH17-AS1, DNAJC25, DNM1L, DPP3, DSN1, DVL2, DZIP3, E2F3, EIF1, ELANE, ELF4, EMC1-AS1, EMC3-AS1, EMP3, EPHA1, EPHB1, ERCC6L, ESR2, ETV7, FAF1-AS1, FAM133DP, FAM71F2, FAM83G, FANCI, FAP, FBN1, FBXL14, FBXL7, FBXO39, FBXO46, FGF13, FGGY, FLJ31356, FRG1BP, FUNDC2, FZD5, GATAD2B, GCC2-AS1, GCSH, GID4, GLIDR, GM2A, GNPTG, GOPC, GPR4, GPX2, GPX7, GS1-204I12.4, GSS, GTF2IP20, GUCY2EP, H1-4, H2BC18, H2BC6, HCAR1, HCG22, HCG4B, HCP5, HDAC9, HEATR1, HES4, HIP1R, HLA-L, HMGCS1, HOMER1, HOTAIRM1, HOXA2, HSD11B1-AS1, HSP90AA1, HSPA14, HSPA1A, HSPA6, HSPA8, HSPA9, HSPE1, HYDIN, IFI30, IL17C, IL6R-AS1, ILF3, INHBA-AS1, INTS3, JARID2-AS1, JMY, JPT2, KANSL1-AS1, KATNBL1, KATNBL1P6, KCNIP1-OT1, KIAA1841, KIF20B, KIR2DL1, KIR2DL2, KIR2DL4, KIRREL2, KLF2, KLHL21, KLHL6, KRTCAP3, KY, LGSN, LILRA4, LINC00656, LINC00885, LINC01115, LINC01136, LINC01191, LINC01691, LINC01740, LINC01792, LINC02034, LINC02319, LINC02399, LINC02537, LINC02757, LINC02773, LOC100129203, LOC100287036, LOC100419583, LOC100506411, LOC100507144, LOC100507554, LOC100996756, LOC100996842, LOC101927178, LOC101927550, LOC101927603, LOC101927811, LOC101927817, LOC101928092, LOC101928120, LOC101928193, LOC101928554, LOC101929066, LOC101929243, LOC101929356, LOC102723447, LOC102723464, LOC104968399, LOC105274304, LOC105369724, LOC105369725, LOC105369803, LOC105369864, LOC105370032, LOC105370635, LOC105370891, LOC105371413, LOC105371490, LOC105371605, LOC105371822, LOC105371932, LOC105372115, LOC105372160, LOC105372569, LOC105372599, LOC105372656, LOC105373190, LOC105373271, LOC105373652, LOC105373656, LOC105373719, LOC105373724, LOC105373794, LOC105373805, LOC105374031, LOC105374217, LOC105374263, LOC105374426, LOC105374906, LOC105374947, LOC105375055, LOC105376001, LOC105376003, LOC105376037, LOC105376076, LOC105376279, LOC105376333, LOC105376344, LOC105376405, LOC105376617, LOC105377033, LOC105377495, LOC105377803, LOC105377929, LOC105378663, LOC105378671, LOC105378758, LOC105379117, LOC105379383, LOC105379476, LOC107984142, LOC107984352, LOC107984510, LOC107984532, LOC107984653, LOC107984871, LOC107985000, LOC107985204, LOC107985309, LOC107985375, LOC107985486, LOC107985679, LOC107985892, LOC107985908, LOC107986413, LOC107986450, LOC107986457, LOC107986871, LOC107986874, LOC107987237, LOC107987244, LOC107987251, LOC107987261, LOC107987438, LOC112267915, LOC112267940, LOC112268170, LOC112268175, LOC112268259, LOC112268340, LOC112268469, LOC154761, LOC643406, LOC646214, LOC728673, LOXL1-AS1, LRP5L, LRRC18, LRRC2, MAATS1, MACC1, MAMDC2, MAML2, MANEAL, MAPKBP1, MBD3, MFSD12, MGC4859, MIEN1, MILR1, MIR101-1, MIR29A, MIR30B, MIR4477A, MIR5094, MIR590, MOB4, MRPS7, MVK, MXRA7, MYH10, NBPF1, NBR2, NCK1-DT, NDUFV2-AS1, NINJ2, NOLC1, NPC2, NPHP3-AS1, NPIPB15, NPR2, NR4A2, NTAN1, NUP210L, NXPE2, ODAPH, OR52K2, OR8G5, PAFAH1B1, PAPPA2, PARN, PCAT18, PCAT29, PCP4L1, PDE2A, PDLIM5, PDZD2, PKD2L2, PLCXD1, PNMT, PPP1R2, PPP4R1L, PRORP, PRPF8, PRRT3, PSMC4, PYCARD-AS1, QRICH2, QSOX2, RBM38, RBMS3, RFPL4A, RFPL4AL1, RGL1, RHOV, RIOX1, RN7SL3, RNF103, RNU6-1, RNU6ATAC, RNY1, RNY4, ROPN1L-AS1, RPGR, RPL21P28, RPL36A, RPS26P11, RPUSD3, RUFY4, RWDD1, SBNO2, SCOC-AS1, SEC22B4P, SENP3, SERF1A, SETD9, SGMS2, SH3RF1, SIRT7, SLC19A2, SLC25A27, SLC25A47, SLC38A7, SLC66A1, SLC66A2, SMIM15, SMIM19, SNHG4, SNX19, SPATA25, SPDL1, SPHK2, SPOUT1, SREBF2, SREBF2-AS1, STARD10, STK38L, STPG1, SYCE3, SYNPO2, SYPL1, TAB2-AS1, TACC2, TAF9, TAPT1, TCEA2, TDP2, TELO2, TENT5B, TGM3, THADA, TIMM17A, TLK2, TM2D3, TMEM191A, TMEM191C, TMEM202-AS1, TMEM251, TMEM45B, TPI1P3, TPM2, TPPP, TRIB1, TRIP12, TRMO, TRPC4AP, TRPV4, TSIX, TSPAN10, TTC3, TTC33, TTC5, TTLL8, TTN-AS1, TUBB3, UBAC2-AS1, UBE2M, UBE2MP1, UBE3D, UCP3, UFM1, ULBP1, URM1, USP31, VAMP2, VTRNA1-3, WDR91, XRRA1, ZCWPW2, ZDHHC19, ZFAND2A, ZFAND3, ZNF10, ZNF114-AS1, ZNF140, ZNF274, ZNF295-AS1, ZNF407, ZNF555, ZNF616, ZNF737, ZSCAN21, ZSWIM8-AS1 |
| thistle1 | AHCYL2, ARHGAP27P1-BPTFP1-KPNA2P3, CD7, CGRRF1, CTSK, DCAF1, FCGR1CP, GDI2, GGA3, GLRX2, GNLY, GNPAT, HECTD3, IGLL5, IK, KIZ, LINC01579, LOC105379326, LOC107984755, LOC107984756, LOC112267905, METTL18, MS4A7, MSR1, MTPAP, MTRF1, MZF1-AS1, NIPBL, PMM1, PRKCQ, PSMA1, SLC12A1, SNORA4, TAOK2, TMEM252-DT, TMEM87B, TMTC1, TNRC18, TOR3A, WDFY2, WDR37, WHAMM, ZNF266, ZNF800 |
| turquoise | AASDH, AASS, ABCA10, ABCB10, ABCB7, ABCC10, ABCC6P1, ABHD10, ABHD11-AS1, ABHD12, ABHD15, ABL1, ACACB, ACAD8, ACADS, ACER2, ACP2, ACP5, ACSM3, ACSS1, ACSS3, ACTL6A, ACTN1, ADA2, ADAL, ADAM10, ADAM19, ADCK2, ADD3-AS1, ADGB, ADGRA2, ADGRE4P, ADGRG2, ADGRG5, AFF3, AFG3L2, AGL, AGPAT5, AHR, AHSP, AIFM1, AK7, AKAP8L, AKIP1, AKR7A2, AKT1, AKT1S1, ALAS2, ALDH1A1, ALDH1B1, ALDH8A1, ALG12, ALG3, ALG5, ALKBH3, ALKBH4, ALOX15, AMMECR1, AMT, ANAPC10, ANKDD1B, ANKEF1, ANKMY2, ANKRD20A12P, ANKRD34A, ANKRD40, ANKRD55, ANKS1B, ANLN, AP3B2, AP4E1, AP4S1, APEX2, APMAP, APOBEC3D, APOBEC3F, APOM, APPL2, ARF5, ARHGAP11B, ARHGAP21, ARHGAP26-AS1, ARHGEF34P, ARHGEF39, ARIH2OS, ARL1, ARL17B, ARMC1, ARMH1, ARSD, ARSK, ASAP2, ASB15, ASB2, ASB9P1, ASCC3, ASF1A, ASGR1, ASGR2, ASMTL, ASTE1, ASXL1, ASXL2, ATAD3A, ATF6B, ATG4D, ATP13A1, ATP2A1, ATP7B, ATP9A, ATRIP-TREX1, ATXN10, AURKB, AXDND1, AXIN1, AXL, B3GALNT1, B3GALT4, B3GLCT, B4GALNT3, BACE1, BAHCC1, BAIAP2L2, BANK1, BAZ1B, BBS10, BBS12, BBS2, BCL2L15, BCL7C, BCS1L, BDH2, BDNF-AS, BDP1, BEAN1, BEND5, BICRA, BLNK, BMP2, BMS1, BNIP5, BOP1, BRD3OS, BRIX1, BTBD7, BTBD8, C10orf88, C11orf49, C11orf65, C12orf10, C12orf29, C12orf57, C12orf65, C12orf66, C12orf76, C16orf54, C16orf58, C16orf87, C17orf100, C17orf75, C19orf47, C19orf48, C1orf105, C1orf109, C1orf112, C1QTNF3, C20orf96, C2CD2L, C2orf16, C2orf92, C3orf14, C3orf33, C4A, C5orf30, C5orf47, C6orf163, C6orf201, C8orf44, C8orf58, C9orf147, CA1, CA4, CACNA1F, CACNA2D3, CALHM2, CAMK1, CAMK2D, CAMP, CAPN15, CAPS, CARD9, CASD1, CASP3, CATSPER1, CATSPERG, CBSL, CBY3, CC2D1A, CCDC103, CCDC121, CCDC137, CCDC14, CCDC142, CCDC144A, CCDC200, CCDC25, CCDC39, CCDC66, CCDC85B, CCHCR1, CCL22, CCNF, CCR7, CD101, CD19, CD1A, CD1C, CD200R1, CD27, CD3EAP, CD3G, CD40, CD52, CD72, CD79B, CD81, CD8A, CDC23, CDC25B, CDC42EP4, CDCA7L, CDK1, CDK10, CDK5, CDK5R1, CDKL1, CDKN2AIPNL, CDPF1, CDYL, CEBPA, CEBPA-DT, CEMIP, CENPE, CENPJ, CENPK, CENPL, CENPQ, CENPT, CENPU, CENPX, CEP152, CEP250, CEP250-AS1, CEP55, CEP85L, CEP89, CEP97, CERS1, CES2, CETN3, CFAP100, CFAP157, CFAP206, CFAP97, CFP, CHAMP1, CHD1L, CHD6, CHDH, CHEK2, CHERP, CHID1, CHIT1, CHMP1B, CHN2, CHROMR, CHST12, CHST13, CHTF18, CIAO2B, CIITA, CIT, CKAP5, CKB, CLCA4-AS1, CLCC1, CLCN3, CLDND2, CLEC10A, CLEC11A, CLEC4C, CLEC4O, CNOT9, CNP, CNR1, CNTNAP3B, CNTNAP3C, COA4, COG2, COG8, COL4A2, COL4A3, COL9A2, COQ10A, COQ5, COQ8A, CORO2A, COX18, CPA3, CPB2-AS1, CPNE4, CPSF4, CPVL, CR1L, CRACR2B, CRAT, CRISP2, CROCCP3, CRYGS, CSE1L, CSF2RA, CSNK2A2, CSRP1, CTAGE1, CTC-338M12.4, CTDSP1, CTPS1, CTTNBP2NL, CUL5, CXCL13, CXorf58, CYB561, CYP1B1-AS1, CYP21A1P, CYP2T1P, CYP4F2, CYP4F29P, CYSLTR2, CYYR1, DAG1, DCLRE1A, DCST2, DCTN5, DDRGK1, DDX10, DDX28, DDX55, DEF8, DEFA3, DEFA4, DEFA8P, DENND5B, DENND6B, DET1, DGCR11, DGCR6, DGCR8, DHFR2, DHRS4, DHRS4L2, DHX57, DIP2A-IT1, DIS3L, DISC2, DKK4, DMAP1, DMPK, DNA2, DNAAF2, DNAJB5-DT, DNAJC9, DNAL4, DNASE1L2, DNASE1L3, DNPEP, DNTTIP1, DOK2, DOLK, DOLPP1, DOP1A, DPH6, DPH7, DPY19L1, DPY19L1P1, DRC3, DRG2, DSCAS, DTWD1, DTX4, DUS1L, DUSP12, DUSP7, DVL1, DYNC2LI1, DYNLRB2, DYRK2, DYRK4, DYTN, DZIP1L, E2F2, EBF1, ECHDC3, ECHS1, EEF1AKNMT, EEFSEC, EFEMP2, EFL1, EFR3B, EI24, EID2, EID2B, EIF2B4, ELAC2, ELP3, ENG, ENPP4, ENTPD6, EP400P1, EPB41L3, EPB42, EPC2, EPHA4, EPHX1, EPM2A, EPRS1, ERBB2, ERCC3, ERCC4, ERMP1, ESF1, ESS2, ETAA1, EXO5, EXOG, EXOSC7, EYA2, FAAH, FAAP24, FABP6, FADS3, FAF1, FAM169A, FAM210B, FAM228A, FAM234B, FAM238A, FAM32A, FAM83F, FAM90A1, FAM95C, FAM98A, FAM98B, FANCB, FANCG, FANCM, FARP1, FARS2, FARSA-AS1, FARSB, FASTKD2, FASTKD3, FBXL5, FBXO24, FBXO45, FBXW4P1, FBXW8, FCER1A, FCRL2, FCRL3, FCRLA, FCSK, FDX1, FECH, FEM1C, FER, FERMT2, FGD3, FGF13-AS1, FGFBP2, FGFR1, FH, FKBP1A, FLACC1, FLJ42627, FLOT2, FLVCR1-DT, FLYWCH1, FMC1-LUC7L2, FMN1, FMNL2, FMO4, FMO5, FOXD2, FOXI1, FRG1CP, FRS3, FSCN3, FTO, FUCA2, FUOM, FURIN, FXR2, G6PC3, GABRB1, GALNT15, GANC, GAS5-AS1, GATA3, GCAT, GCFC2, GCLC, GCN1, GCNT4, GEMIN4, GEN1, GFER, GFI1B, GGTA1P, GIGYF2, GIMAP1, GIMAP6, GIMAP7, GIMAP8, GIPR, GLCE, GLG1, GLI1, GLOD4, GMPR, GMPR2, GNAL, GNB3, GNG12-AS1, GNGT2, GNPNAT1, GNPTAB, GOLGA8A, GOLGA8B, GORAB, GPATCH2, GPC2, GPN2, GPR137, GPR162, GPR82, GPRC5C, GRHL1, GRHL3, GRIP2, GRK2, GSTM1, GSTM2, GSTM4, GTF2H3, GTF2IRD2, GTF3C3, GTSE1, GUCY2D, GUSBP16, GZMA, H1-6, H2AC15, H2AW, H2BC15, H2BP2, H3C10, H3C13, H3P4, H4C11, H4C12, H4C15, H4C4, HAAO, HARBI1, HAUS3, HBA1, HBA2, HBB, HBD, HBM, HBQ1, HCFC2, HCG11, HDAC8, HDC, HDGFL2, HEATR4, HELLS, HEMGN, HEMK1, HERC2P2, HEXIM2, HIC2, HIPK1-AS1, HIRIP3, HJURP, HLA-DMA, HLTF, HMGN3, HMGXB3, HOMER2, HOMEZ, HOXA4, HPF1, HS6ST1, HSD17B1, HSF2, HTT, HYAL3, HYLS1, IBA57, IBTK, ICAM3, ICE2, IDE, IFITM5, IFT122, IGF1R, IKZF3, IKZF4, IL22RA2, IMPDH1, IMPG2, INKA1, INPP5F, INPP5J, INTS2, INTS6-AS1, IPO9, IQCG, IQCH-AS1, IRF4, IRF8, IST1, ITGA1, ITGA10, ITGA6, ITGA9-AS1, ITIH4, ITM2C, ITPA, IVD, JOSD2, JUNB, JUP, KCNAB1, KCNC4, KCNE1B, KCNE3, KCNJ1, KCNMA1, KIAA0895, KIAA1211L, KIAA1328, KIAA1522, KIAA1586, KIAA1958, KIF11, KIF23, KIF24, KIFAP3, KLF8, KLHDC3, KLHDC4, KLHL11, KLHL13, KLHL17, KLHL20, KLRB1, KLRC2, KLRC3, KMT5A, KNOP1, L3HYPDH, L3MBTL1, LACTB2-AS1, LAMA2, LAMP2, LAS1L, LAT2, LCK, LCMT1-AS1, LCN2, LEMD2, LETMD1, LGALS12, LGALS2, LGALS8-AS1, LGI2, LILRA2, LINC00174, LINC00205, LINC00266-1, LINC00299, LINC00339, LINC00471, LINC00649, LINC00667, LINC00680, LINC00899, LINC00926, LINC00996, LINC01006, LINC01037, LINC01126, LINC01333, LINC01352, LINC01504, LINC01547, LINC01612, LINC01781, LINC01871, LINC01890, LINC01970, LINC01973, LINC01993, LINC02166, LINC02218, LINC02255, LINC02256, LINC02363, LINC02432, LINC02454, LINC02458, LINC02561, LINC02569, LINC02610, LINC02734, LINC02785, LINC02836, LINC02856, LINC02863, LIPT2, LIX1-AS1, LMBRD2, LMNB2, LMTK2, LOC100129484, LOC100130172, LOC100132356, LOC100133331, LOC100288846, LOC100289333, LOC100294145, LOC100505622, LOC100505716, LOC100505909, LOC100506551, LOC100507412, LOC100507551, LOC100652768, LOC100996643, LOC101926892, LOC101926977, LOC101927322, LOC101927330, LOC101927345, LOC101927454, LOC101927478, LOC101927509, LOC101927552, LOC101927573, LOC101927609, LOC101927864, LOC101928059, LOC101928068, LOC101928212, LOC101928383, LOC101928389, LOC101928415, LOC101928462, LOC101928504, LOC101928517, LOC101928864, LOC101928972, LOC101929054, LOC101929117, LOC101929255, LOC101929270, LOC101929309, LOC101929431, LOC101929540, LOC101929613, LOC101929691, LOC101929692, LOC101929718, LOC101929798, LOC101929805, LOC102467080, LOC102606465, LOC102723506, LOC102723714, LOC102723739, LOC102723811, LOC102723885, LOC102724015, LOC102724080, LOC102724104, LOC102724146, LOC102724330, LOC102724358, LOC102724378, LOC102724497, LOC102724528, LOC102724596, LOC102724708, LOC102724748, LOC102724765, LOC102724770, LOC102724881, LOC102724919, LOC102725021, LOC105369149, LOC105369299, LOC105369351, LOC105369477, LOC105369519, LOC105369535, LOC105369612, LOC105369625, LOC105369714, LOC105369736, LOC105369755, LOC105369783, LOC105369957, LOC105369960, LOC105370029, LOC105370348, LOC105370437, LOC105370513, LOC105370624, LOC105370658, LOC105370687, LOC105370707, LOC105370790, LOC105370807, LOC105370819, LOC105371168, LOC105371224, LOC105371254, LOC105371378, LOC105371442, LOC105371498, LOC105371622, LOC105371716, LOC105371785, LOC105371816, LOC105371856, LOC105371871, LOC105371985, LOC105372107, LOC105372117, LOC105372249, LOC105372250, LOC105372401, LOC105372449, LOC105372520, LOC105372631, LOC105372649, LOC105372688, LOC105372738, LOC105372859, LOC105372860, LOC105372880, LOC105372903, LOC105373024, LOC105373195, LOC105373233, LOC105373323, LOC105373431, LOC105373570, LOC105373618, LOC105373689, LOC105373925, LOC105374245, LOC105374257, LOC105374334, LOC105374341, LOC105374368, LOC105374414, LOC105374419, LOC105374465, LOC105374545, LOC105374584, LOC105374777, LOC105374800, LOC105374808, LOC105374898, LOC105374938, LOC105374965, LOC105375010, LOC105375044, LOC105375059, LOC105375114, LOC105375196, LOC105375330, LOC105375422, LOC105375470, LOC105375500, LOC105375513, LOC105375634, LOC105375675, LOC105375785, LOC105375928, LOC105376136, LOC105376146, LOC105376266, LOC105376364, LOC105376527, LOC105376542, LOC105376704, LOC105376882, LOC105377046, LOC105377205, LOC105377267, LOC105377452, LOC105377633, LOC105377683, LOC105377691, LOC105377766, LOC105377872, LOC105377885, LOC105378298, LOC105378360, LOC105378577, LOC105378632, LOC105378687, LOC105378719, LOC105378751, LOC105378976, LOC105379283, LOC105379322, LOC105379356, LOC105379426, LOC105379461, LOC105379508, LOC105379521, LOC105379854, LOC107983990, LOC107984037, LOC107984138, LOC107984139, LOC107984176, LOC107984192, LOC107984244, LOC107984312, LOC107984344, LOC107984442, LOC107984643, LOC107984757, LOC107984774, LOC107984889, LOC107984932, LOC107984950, LOC107985013, LOC107985082, LOC107985219, LOC107985262, LOC107985284, LOC107985305, LOC107985320, LOC107985323, LOC107985562, LOC107985576, LOC107985667, LOC107985694, LOC107985701, LOC107985712, LOC107985769, LOC107985770, LOC107985871, LOC107985877, LOC107985897, LOC107985900, LOC107985911, LOC107985923, LOC107985942, LOC107986008, LOC107986168, LOC107986245, LOC107986348, LOC107986516, LOC107986528, LOC107986660, LOC107986824, LOC107986854, LOC107986858, LOC107986929, LOC107986973, LOC107986997, LOC107987080, LOC107987121, LOC107987222, LOC107987399, LOC107987461, LOC108783645, LOC108783654, LOC112267983, LOC112267988, LOC112268024, LOC112268052, LOC112268090, LOC112268103, LOC112268168, LOC112268182, LOC112268237, LOC112268239, LOC112268243, LOC112268256, LOC112268258, LOC112268278, LOC112268295, LOC112268301, LOC112268317, LOC112268330, LOC112268446, LOC112543491, LOC114841040, LOC148696, LOC150776, LOC199882, LOC220729, LOC440700, LOC728554, LOC728743, LOC728975, LOC729683, LOC730098, LPL, LRATD2, LRIG2, LRP12, LRP3, LRRC40, LRRC61, LRRC7, LRRN1, LSG1, LSMEM2, LTBP3, LTF, LY86, LYPD8, M1AP, MAB21L3, MAD1L1, MAGEH1, MAN1B1, MANEA, MAP10, MAP1A, MAP3K5-AS1, MAP4K5, MAP6D1, MAP7D3, MAPKAPK5-AS1, MARCHF8, MAST2, MBD4, MBIP, MBNL3, MCC, MCF2, MCUB, MCUR1, MDFIC, MDGA1, MED4-AS1, MEGF11, METTL17, METTL21C, METTL3, MEX3C, MFSD4B, MGAT4B, MGMT, MHENCR, MICB, MIEF1, MIEF2, MIER3, MIGA2, MINDY1, MIPEPP3, MIR10522, MIR25, MIR3150BHG, MIR324, MIR372, MIR3936HG, MIR4481, MIR4494, MIR4716, MIR4802, MIR5696, MIR6129, MIR617, MIR646, MIR650, MIR6529, MIR6781, MIR6820, MIR6847, MKI67, MKLN1-AS, MLC1, MLEC, MLLT11, MLLT3, MMAB, MMP12, MMP19, MMP25-AS1, MMP27, MMP8, MMS19, MMS22L, MNAT1, MORC2, MORC4, MPHOSPH9, MPI, MPL, MPP6, MPV17, MRC1, MRFAP1L1, MRM2, MRPL10, MRPL11, MRPL2, MRPL20-AS1, MRPL30, MRPL34, MRPL38, MRPL40, MRPL41, MRPL58, MRPS17, MRPS18B, MRPS23, MRPS27, MRPS35, MRRF, MRS2, MRTO4, MS4A1, MS4A14, MS4A4A, MSC-AS1, MSH2, MTERF4, MTLN, MTMR11, MTRES1, MTRNR2L1, MTRNR2L3, MTSS1, MTX2, MTX3, MUC13, MUC20, MUSTN1, MXD4, MYADM-AS1, MYB, MYBL1, MYCBP2-AS1, MYEF2, MYO16, MYO16-AS1, MYO7B, MYPOP, MYRIP, N4BP2L2-IT2, N6AMT1, NAA15, NAA30, NAA80, NAALADL1, NACC2, NAE1, NAGA, NAGLU, NAPA-AS1, NAT14, NAXE, NBDY, NBR1, NCAPD2, NCAPD3, NCDN, NCF1, NCF1B, NCF4-AS1, NCOA4, NCR3, NDC1, NDE1, NDST1-AS1, NDST3, NDUFAF6, NDUFAF8, NDUFS4, NEIL1, NEK3, NEK5, NEMP2, NETO2, NF2, NFATC2, NHLRC1, NHS, NHSL2, NIBAN1, NIBAN3, NIF3L1, NIFK, NIFK-AS1, NIPAL3, NIPSNAP3A, NIPSNAP3B, NIT2, NKAPP1, NLRP12, NME3, NMT2, NNT, NNT-AS1, NOL11, NOL3, NOP9, NR1H3, NR2C1, NR2C2AP, NR6A1, NRBP2, NREP, NSD2, NSMF, NSUN5P1, NUDT14, NUDT15, NUDT3, NUDT7, NUFIP1, NUP210, NUP35, NUP37, NUP42, NXNL2, ODF2, OGG1, OGN, OIP5, OLAH, OLFM2, OLFM4, OLFML2B, OMG, OPRL1, OR10V2P, OR2B11, OR52K1, OR6N1, ORAI2, OSBPL11, OSBPL6, OSBPL7, OTUD1, OTUD3, P2RX5, P3H1, P4HTM, PA2G4, PA2G4P4, PACC1, PALLD, PAN2, PANX1, PAQR4, PARGP1, PARGP1-AGAP4, PARP15, PARP16, PARP3, PARTICL, PATJ, PAXBP1-AS1, PBX1, PBX3, PCAT6, PCBP2-OT1, PCCB, PCED1A, PCED1B, PCGF6, PCID2, PCK2, PCNA, PCNT, PCOLCE, PDCD6, PDCD7, PDE6C, PDE6G, PDF, PDGFD, PDPN, PDRG1, PDSS1, PELO, PEX12, PEX16, PEX26, PFKM, PGAM1, PGAM4, PGBD2, PGP, PHF7, PIGL, PIGO, PIGU, PIH1D2, PINK1, PINK1-AS, PINX1, PIP, PIP4K2B, PISD, PJVK, PKD1L1, PKIG, PKN3, PLA2G2C, PLBD1-AS1, PLCB1, PLCD1, PLD4, PLEKHG1, PLEKHH1, PLXND1, PMFBP1, PMM2, PMS2P9, PNMA6A, PNPLA4, PNRC2, POGLUT1, POLE2, POLL, POLN, POLR2E, POLR3D, POLR3F, POLR3G, POLR3H, POLRMT, POMT1, PON2, POU2AF1, PPARA, PPARGC1B, PPBP, PPDPF, PPEF1, PPFIBP2, PPP1CA, PPP1R16B, PPP1R37, PPP3CC, PREB, PREP, PRICKLE1, PRKAB1, PRKAB2, PRKACB, PRKX, PRMT1, PRMT7, PROC, PROCA1, PROM1, PROS1, PROSER1, PROX2, PRPF19, PRPF31, PRPH2, PRR11, PRR12, PRR14, PRR5, PRRC2B, PRSS33, PRSS41, PRSS53, PSD4, PSMB8-AS1, PSTK, PTGDR2, PTGR2, PTPMT1, PTPN9, PTPRO, PURA, PVT1, PWP1, PWWP2B, PXDC1, PXN-AS1, PXYLP1, PYHIN1, QPCT, QSER1, RAB14, RAB30, RAB4A, RACGAP1, RAD51D, RAD54L2, RAPSN, RASA4B, RASAL2, RASSF7, RBL1, RBM12B, RBM15-AS1, RBM15B, RBM41, RBM48, RBM5, RBSN, RCAN3AS, RCC1L, RCCD1, RCL1, RDH13, RECK, RFC4, RFX7, RFX8, RGS19, RHBDD3, RHD, RHNO1, RHOBTB3, RIPOR3, RITA1, RMND5B, RNASEH1-AS1, RNASEH2B, RNF10, RNF151, RNF214, RNF216P1, RNF217, RNF26, RNF5, RNF8, RNU5A-1, RNU5D-1, RNU5E-1, RNVU1-14, ROGDI, RPARP-AS1, RPE, RPL31P11, RPP38, RPS6KA4, RPUSD1, RPUSD4, RRM1, RRP1B, RSL1D1, RSPH4A, RTCB, RTEL1, RTL5, RTL8A, RTN4IP1, RUFY3, RUNDC3A, RUVBL1, RWDD3, RYR1, S100A7A, SAMD1, SAMD3, SAMM50, SAP130, SAP30, SAYSD1, SCAI, SCAMP3, SCGB3A1, SCIMP, SCMH1, SCO1, SCRG1, SCRN1, SDAD1, SDHAF1, SEC24B-AS1, SELENBP1, SELENOP, SEMA6A, SEMA6A-AS1, SEPHS1, SEPTIN1, SEPTIN4, SERGEF, SERINC1, SERPINA9, SERPINB10, SERPINE3, SERPINF1, SETD7, SETSIP, SF3A2, SF3B3, SFTPB, SFXN2, SGPP1, SGSM3, SH2B1, SH2D3C, SH2D4A, SH2D4B, SH3YL1, SHQ1, SIGLEC10, SIGLEC12, SIGLEC15, SIGLEC17P, SIGLEC6, SIGLEC8, SIN3B, SIPA1, SIRPB1, SIRPB2, SIT1, SKAP1, SKAP2, SKP2, SLAIN1, SLC16A12, SLC16A14, SLC16A7, SLC22A13, SLC22A15, SLC23A1, SLC25A1, SLC25A20, SLC25A42, SLC26A8, SLC27A3, SLC27A4, SLC29A1, SLC29A3, SLC29A4, SLC2A11, SLC2A4RG, SLC35C1, SLC35F6, SLC35G2, SLC38A9, SLC4A5, SLC6A12, SLC6A16, SLC6A8, SLC7A8, SLC9A9, SLFN11, SLFN13, SLMAP, SLX4, SMAD1, SMAD5, SMARCAD1, SMARCB1, SMG1P7, SMG6, SMG8, SMIM10, SMIM10L1, SMIM11A, SMIM18, SMIM30, SMPD4, SMS, SNAP47, SNAPC3, SNCA, SNHG17, SNHG21, SNORA16B, SNORA21, SNORA40, SNORA53, SNORA64, SNORA73B, SNORA81, SNORD12, SNORD12B, SNORD12C, SNORD13J, SNORD1B, SNORD36B, SNORD36C, SNORD59A, SNORD9, SNORD96A, SNRK-AS1, SNRPA1, SNX12, SNX22, SOAT1, SORCS2, SOWAHD, SOX5-AS1, SP1, SPACA9, SPATA21, SPATA41, SPC24, SPDYE11, SPDYE4, SPESP1, SPIN3, SPNS3, SPON2, SPRED1, SPRY3, SPRYD7, SPSB2, SPTA1, SRGAP1, SRPRB, SRSF8, STAC3, STAG3, STAG3L1, STAG3L2, STAP1, STARD5, STK11IP, STOML1, STON1, STPG2, STPG4, STS, STX18, STX2, SUCLA2, SUGT1, SULT1A1, SYCP2L, SYCP3, SYMPK, SYNJ2BP, SYT2, TAB1, TACR2, TADA1, TAF1B, TAF4, TAF6, TANGO6, TARBP1, TARBP2, TASP1, TBC1D12, TBC1D13, TBC1D19, TBC1D31, TBC1D9, TBCB, TBCEL, TBCK, TC2N, TCF12, TCF3, TCHP, TCTEX1D1, TCTN2, TEAD3, TENT4A, TERB2, TEX2, TEX29, TFB2M, TFF3, TGDS, THAP11, THAP3, THAP8, THEM4, THOC1, THOC3, THOC6, THOP1, THYN1, TIAM1, TIFAB, TIGD1, TIMELESS, TIMM13, TIMM22, TIMM23B, TIMM50, TLCD4, TLE1, TLE5, TLR7, TLR9, TMA16, TMCC1-AS1, TMCC2, TMED11P, TMEM104, TMEM141, TMEM14A, TMEM156, TMEM19, TMEM192, TMEM203, TMEM209, TMEM223, TMEM236, TMEM245, TMEM254, TMEM256, TMEM263, TMEM269, TMEM42, TMEM62, TMEM63C, TMEM79, TMEM80, TMEM86B, TMEM94, TMX1, TNFAIP8L1, TNK2-AS1, TNKS2-AS1, TNS3, TOB1, TOB1-AS1, TOMM70, TOX, TP53I3, TP53TG1, TPD52, TPTE2P5, TPTEP2, TPX2, TRAPPC6A, TRIAP1, TRIM35, TRIM4, TRIM44, TRIM58, TRIM59, TRIM68, TRIM8, TRIP6, TRMT11, TRMT13, TRMT2B, TRMU, TRPV1, TSC22D3, TSEN15, TSNAX-DISC1, TSPAN6, TSPEAR, TTC13, TTC16, TTC21B, TTC27, TTC38, TTC39B, TTC7A, TTC8, TUBD1, TUBG1, TVP23B, TVP23C, TWF2, UBA1, UBE2Q2, UBE2Q2P1, UBL4A, UBN2, UFL1, UGDH, UGT8, UHRF1BP1, ULK4P1, UNC80, UPRT, UROD, USP14, USP34, VASH1, VASH2, VAV2, VCPIP1, VDAC2, VEPH1, VEZT, VLDLR, VPS33B, VPS35L, VPS4B, VPS52, VPS9D1-AS1, VRK3, VSTM4, VWA8, WASIR2, WBP1, WBP1L, WDCP, WDR11, WDR18, WDR3, WDR36, WDR4, WDR46, WDR5, WDR6, WDR7, WDR7-OT1, WDR75, WDR83, WDR89, WDSUB1, WEE2-AS1, WHAMMP2, WNK2, WRAP53, WWP1, XIST, XK, XPA, XPOT, XRCC3, XXYLT1, YARS2, YIPF2, ZBED3, ZBED4, ZBED6CL, ZBED8, ZBTB11-AS1, ZBTB14, ZBTB20-AS4, ZBTB38, ZBTB41, ZBTB45, ZBTB5, ZBTB6, ZBTB8B, ZDHHC11, ZDHHC23, ZFP28, ZFYVE19, ZGLP1, ZIK1, ZKSCAN3, ZKSCAN7, ZMYM4, ZNF134, ZNF14, ZNF169, ZNF17, ZNF180, ZNF2, ZNF212, ZNF213-AS1, ZNF22, ZNF225, ZNF227, ZNF234, ZNF26, ZNF268, ZNF282, ZNF321P, ZNF322, ZNF326, ZNF335, ZNF350, ZNF362, ZNF366, ZNF367, ZNF383, ZNF398, ZNF415, ZNF416, ZNF428, ZNF436, ZNF443, ZNF446, ZNF449, ZNF470, ZNF473, ZNF484, ZNF485, ZNF500, ZNF526, ZNF529-AS1, ZNF542P, ZNF543, ZNF544, ZNF549, ZNF550, ZNF558, ZNF565, ZNF567, ZNF568, ZNF574, ZNF582, ZNF589, ZNF595, ZNF600, ZNF606, ZNF607, ZNF621, ZNF623, ZNF624, ZNF628, ZNF630, ZNF649, ZNF649-AS1, ZNF658, ZNF667-AS1, ZNF669, ZNF677, ZNF688, ZNF692, ZNF714, ZNF721, ZNF736, ZNF740, ZNF747, ZNF764, ZNF768, ZNF772, ZNF775, ZNF783, ZNF784, ZNF786, ZNF788P, ZNF799, ZNF8, ZNF829, ZNF83, ZNF835, ZNF845, ZNF846, ZNF852, ZNF865, ZNF880, ZNF888, ZRANB2-AS2, ZSCAN22, ZSCAN30, ZSWIM3, ZZZ3 |
| violet | ANKZF1, AP2A2, ARHGAP27, ARMCX5-GPRASP2, ASB6, ATXN1L, BEX1, BTBD1, C19orf25, C3orf38, CASC1, CCNG2, CCR5, CDC34, CDH13, CEBPG, CGAS, CHAF1B, CIPC, COX19, CSTF3, DAGLB, DCK, DMRTC2, DNAJC2, DVL3, EVA1B, FAM114A1, FAM114A2, FAM168B, FBXO25, FCGR2B, FGFR1OP2, FYCO1, GCC1, GGT3P, GNRH1, GPN1, GSK3A, H4C3, HIC1, HP11014, HTATSF1P2, ING2, KANSL1, KANSL1L, KBTBD2, KCTD9, KDM7A-DT, KEAP1, KIFBP, KPNA3, KPNA6, LAMP1, LMO2, LOC100287497, LOC101927949, LOC101928323, LOC102724979, LOC105370861, LOC105370943, LOC105375034, LOC105376959, LOC105378756, LOC107985279, LOC107985423, LOC107985481, LOC107987128, LOC112267855, LOC374443, LOC401261, LY86-AS1, LYRM2, MAGED2, MCPH1, MINDY2, MTMR12, NAIF1, NAT1, NCBP2AS2, NIP7, NOXRED1, NUDT18, NUDT4, NUSAP1, OLIG1, OLIG2, OPTN, PAK1IP1, PDP1, PEX19, PIP5K1A, PLEC, PLEKHA2, POLDIP3, PPTC7, PROSER3, PSMD5, RAD1, RAD52, RAP2A, RBBP4, RDH5, RHOQ, RMND5A, RNH1, RPS28, SAMD8, SENP1, SERAC1, SIGIRR, SLC25A16, SLC35A4, SLC41A2, SLC7A11, SLFN5, SMIM3, SNORD102, SPTY2D1OS, STK35, SUDS3, TAP2, TBC1D25, TGS1, TMEM128, TMEM183B, TMEM268, TP53BP2, TPRG1-AS1, TRAPPC13, TRIM41, TSHZ3, TTL, TUG1, UBE2D2, UBE2Q1, UQCRFS1, USP48, VPS37C, WDR59, WDR5B, ZCCHC18, ZNF16, ZNF174, ZNF213, ZNF226, ZNF28, ZNF333, ZNF561, ZNF81, ZNF844, ZSCAN9 |
| white | AAAS, AAK1, ABCA7, ABCB9, ABCF1-DT, ABHD16B, ABL2, ABTB2, ACAP1, ACER3, ACOT11, ACOT7, ADGRL1, ADO, ADPGK, ADPGK-AS1, AFF4, AHNAK2, AIF1L, AIPL1, AK2, AMBRA1, ANKRD13B, ANKRD20A4-ANKRD20A20P, ANKRD33, ANO6, ANO7L1, ANP32C, AP1B1, ARAP1-AS2, AREL1, ARGFXP2, ARHGAP31, ARHGEF17, ARL4C, ARMC10, ARPC1A, ASH1L-AS1, ATP10D, ATP1A1, ATP2A2, ATP6, ATP6AP2, ATP6V0A1, ATP6V1A, ATP8, ATP8B5P, ATXN1, AUTS2, AVPI1, AVPR2, AZGP1, B3GAT3, B3GNT3, BAHD1, BATF, BCL6, BRD4, BRI3, BTBD6, C11orf91, C12orf73, C17orf64, C1orf194, C1orf54, C1S, C8orf88, CA5BP1-CA5B, CACNA1G, CAMKMT, CAMSAP1, CAPN10, CAPN12, CAPNS2, CBFA2T2, CBX5, CCDC134, CCDC167, CCDC50, CCDC85C, CCDC88C, CCND2, CD300A, CD320, CD69, CD96, CDC14B, CDC37L1, CDK16, CELA2B, CELF3, CELSR2, CEP104, CHIC2, CHKB, CHMP4C, CHMP7, CHST7, CIC, CILP, CKLF-CMTM1, CKS2, CLCN7, CLEC4A, CLHC1, CLIC3, CLU, CMC2, CNDP2, CNFN, CNNM4, COA7, COA8, COLGALT2, COPB2, CORO1C, COX1, COX16, COX2, COX20, COX3, CPA4, CRAMP1, CRCT1, CRY2, CRYBB2, CSF2RB, CSRP2, CSTF2, CTBP2, CTC1, CTDP1, CTNNBIP1, CTSC, CUL7, CXADR, CYP2B7P, CYP2D6, CYP4V2, CYSRT1, CYTB, CYTIP, DCDC2B, DEFA1B, DEFB103A, DEFB103B, DENND2C, DENR, DEPDC4, DGKE, DGUOK, DIAPH1-AS1, DLEC1, DNAH9, DNAJC27-AS1, DNHD1, DNMT1, DPH2, DPY19L2, DTD2, DYNLL1, ECHDC1, ECT2, EED, EEF1A1, EEF1E1, EHD4-AS1, EHF, EIF3A, EIF3E, EIF4A3, EIF5A, EIF5AL1, EIPR1, ELK3, ELL, ELMO3, EMC2, EMP2, ENTPD5, EOGT, EPM2AIP1, EPS15, EPS8L1, ERI3, ERMN, ERV3-1, ESYT2, ETFRF1, EYA3, EZH2, F12, F2R, FABP2, FAHD2A, FALEC, FAM102A, FAM106A, FAM118A, FAM124A, FAM131A, FAM193B, FAM209B, FAM20B, FAM25A, FAM25E, FAM3A, FAM49B, FAM76A, FAM83A, FAM83D, FAM95B1, FANCC, FBXL15, FBXO32, FBXO4, FBXW7, FCGBP, FKBP7, FKBPL, FNBP1L, FOXD4, FOXK2, FOXQ1, FPR3, FSD2, FTL, FUT6, FZD3, GABRR2, GADD45G, GAK, GARS-DT, GBA2, GEM, GFM2, GGT8P, GGTLC2, GINM1, GIPC1, GLCCI1-DT, GLS, GNAZ, GNB2, GNB5, GNG12, GOLGA7B, GPAT2, GPR156, GPR37L1, GPRC5A, GPSM1, GPX4, GRIP1, GSDMA, GSG1L, GSPT1, GSTZ1, GTF2IRD1P1, GTF3C6, GYG1, GZF1, H2AC16, H2AC18, H2AC19, H2AX, H4C5, HACE1, HAUS5, HCFC1R1, HCST, HEPHL1, HERPUD1, HIBADH, HINT1, HLA-DRB3, HM13, HMGCL, HNRNPA1L2, HNRNPCL1, HNRNPM, HOOK2, HPRT1, HS3ST1, HSD17B14, HYMAI, IL4R, IL7R, INPP4A, IRF6, ITPR1, KATNA1, KCNE5, KCNH3, KCNRG, KCNS1, KDELR2, KHDRBS1, KIAA0319, KIRREL3, KLF5, KLK6, KLK8, KLK9, KLRD1, KPRP, KRT16P3, KRT5, KRT8, KRT80, KRTDAP, L3MBTL3, LAMA4, LARS1, LCE1F, LCE3A, LCE3D, LCE3E, LGALS1, LIAS, LILRP2, LIMD1, LINC00115, LINC00211, LINC00869, LINC00880, LINC01000, LINC01108, LINC01203, LINC01214, LINC01331, LINC01791, LINC01943, LINC02043, LINC02100, LINC02328, LINC02413, LINC02568, LINC02604, LINC02827, LIPC-AS1, LOC100128276, LOC100129027, LOC100132781, LOC100134423, LOC100190986, LOC100288069, LOC100505736, LOC100505915, LOC100506098, LOC100506321, LOC100506804, LOC100507103, LOC100996720, LOC100996740, LOC101060341, LOC101927018, LOC101927066, LOC101927272, LOC101927391, LOC101928008, LOC101928032, LOC101928317, LOC101928617, LOC101928626, LOC101929057, LOC101929908, LOC102723678, LOC102723709, LOC102723728, LOC102723809, LOC102724262, LOC102724334, LOC102724591, LOC102724805, LOC105369161, LOC105369199, LOC105369313, LOC105369753, LOC105369905, LOC105370088, LOC105370534, LOC105370821, LOC105370964, LOC105371056, LOC105371082, LOC105371090, LOC105371362, LOC105371692, LOC105371711, LOC105371795, LOC105371894, LOC105371903, LOC105372098, LOC105372435, LOC105372480, LOC105372698, LOC105373017, LOC105373137, LOC105373511, LOC105374063, LOC105374303, LOC105374304, LOC105374454, LOC105374745, LOC105374775, LOC105374790, LOC105374807, LOC105374855, LOC105374986, LOC105374995, LOC105375334, LOC105375532, LOC105375787, LOC105375942, LOC105376063, LOC105376070, LOC105376139, LOC105376341, LOC105376611, LOC105376717, LOC105377103, LOC105377320, LOC105377499, LOC105377743, LOC105377896, LOC105377937, LOC105378347, LOC105378487, LOC105378536, LOC105378573, LOC105378954, LOC105378963, LOC105379102, LOC105379346, LOC105379554, LOC105747689, LOC107983950, LOC107984328, LOC107984659, LOC107984660, LOC107984852, LOC107984880, LOC107984910, LOC107985010, LOC107985224, LOC107985268, LOC107985306, LOC107985367, LOC107985395, LOC107985416, LOC107985484, LOC107985551, LOC107985780, LOC107985873, LOC107985876, LOC107986080, LOC107986119, LOC107986141, LOC107986435, LOC107986598, LOC107986656, LOC107986924, LOC107986930, LOC107986941, LOC107987046, LOC107987097, LOC107987299, LOC112267874, LOC112268035, LOC112268101, LOC112268104, LOC112268124, LOC112268130, LOC112268245, LOC284950, LOC285500, LOC285638, LOC341056, LOC441087, LOC441242, LOC643339, LOC729218, LOR, LOXL3, LRP11, LRRC41, LRRC69, LRRCC1, LRWD1, LSM2, LSP1P4, LSR, LYG1, LYN, MADD, MAGOH, MAP2K4P1, MAP3K9, MAP7, MAPKAPK2, MAPRE3-AS1, MCM10, MDC1, MDK, MED29, METRNL, METTL21A, METTL2A, METTL2B, MFSD2B, MIPEP, MIR1244-4, MIR155HG, MIR1915HG, MIR210HG, MIR3135A, MIR3682, MIR3909, MIR3925, MIR4308, MIR4426, MIR4435-2HG, MIR4651, MIR558, MIR6514, MIR657, MIR6755, MIR6842, MIR7113, MLANA, MON1A, MON2, MORN1, MPO, MPV17L2, MREG, MROH6, MRPL33, MRPL37, MRPL48, MRPL52, MRPL55, MRS2P2, MSMO1, MT1X, MTCH1, MTHFR, MTHFS, MTRNR2L6, MTRNR2L8, MTRNR2L9, MTX1, MYBBP1A, MYLPF, MYO6, MYO9B, MYOF, N4BP2, NAB1, NANP, NAT10, NBPF11, NCEH1, NCOA5, ND1, ND2, ND3, ND4, ND4L, ND5, ND6, NDFIP2, NDUFA3, NDUFS8, NEAT1, NELFE, NEMP1, NEU3, NFKBIB, NFKBIL1, NGRN, NIPAL1, NOC4L, NOL9, NOMO1, NOTCH2NLR, NOTCH4, NPHP3, NSMCE1, NT5DC4, NUP107, NUPR1, NUTM2E, ODC1, OGFOD2, OGFOD3, OMA1, OOSP1, OSCP1, PAG1, PAK2, PAK4, PAOX, PAPSS2, PARD6G, PARP10, PBLD, PCDH11Y, PCYOX1L, PDCL3P4, PDGFA, PDIK1L, PDLIM1, PDZK1IP1, PELI3, PFDN5, PGGHG, PHF19, PHF23, PHYHD1, PI4KAP2, PICK1, PID1, PIK3CD-AS2, PIM1, PIM2, PITPNA, PIWIL4, PKIB, PKP1, PLA2G4D, PLAC8, PLGLA, PLLP, PLXNA3, PMAIP1, PMS2P1, POLE3, POU2F2, POU5F1P3, PPA1, PPFIBP1, PPIAL4H, PPM1N, PPP1R16A, PPP1R2B, PPP1R3C, PRC1, PRELID1, PRKCA, PRLR, PRMT3, PROM2, PRXL2B, PSMA6, PSMC5, PSPH, PTCD3, PTCSC1, PTK6, PTOV1, PTP4A1, PTPRA, PTS, PTTG1IP, PTX3, PWARSN, PYGO2, RAB33A, RAB3A, RAB3GAP1, RAB3IL1, RAC2, RAD23A, RAMP3, RBBP8, RBM19, RC3H2, RCHY1, RECQL4, RELA, RELA-DT, RFK, RGSL1, RHBDD2, RHCE, RHCG, RHEB, RHOB, RHOF, RHOG, RHOT2, RMND1, RN7SK, RNA45SN2, RNF19B, RNF39, RNFT1-DT, ROBO3, RP9, RPAP2, RPIA, RPL23P8, RPL32P3, RPL8, RPLP1, RPLP2, RPP14, RPS15A, RPS16, RPS20, RPUSD2, RRP7BP, RSF1, RXRB, S100A14, SBDS, SBDSP1, SCAMP4, SCARNA9L, SCNN1B, SCNN1D, SCRT2, SDAD1P1, SDC2, SDCBP2, SDE2, SEC14L1P1, SEC61G, SEC63, SERF2, SERPINB3, SERPINB9P1, SERTAD2, SETX, SF1, SH3BGRL, SHE, SHMT2, SHOC1, SIDT2, SIGLEC16, SIMC1, SLAMF9, SLBP, SLC12A9, SLC20A1, SLC25A25, SLC25A26, SLC25A52, SLC25A6, SLC2A5, SLC35G3, SLC35G5, SLC39A11, SLC7A14, SLCO5A1, SLMO2-ATP5E, SLPI, SLU7, SLURP1, SMAD3, SMDT1, SMG1P6, SMN2, SNHG30, SNORA10, SNORA29, SNORA31, SNORA59B, SNORA92, SNORD11, SNORD141A, SNORD14C, SNORD3A, SNORD54, SNORD60, SNORD63, SOCS3, SOWAHC, SPAG4, SPDYA, SPDYE2B, SPECC1L, SPG11, SPINK7, SPRED2, SPRR1B, SPRR2B, SPRR2D, SPRR3, SPTBN2, SRC, SRP72, SRRM5, ST20, STAM, STIM2-AS1, STK39, STMP1, STX12, STX17, STX6, SUB1, SULT2B1, SYNRG, SYS1, SYT7, TAB3-AS1, TACO1, TACSTD2, TAF5, TARP, TBC1D3G, TBC1D3L, TCEAL9, TCF25, TDRD3, TEAD2, TEKT4P2, TFAM, TFE3, TGFBR1, TGFBR3L, THAP9, THG1L, THUMPD3-AS1, TIFA, TM7SF2, TMED10, TMEM238L, TMEM252, TMEM253, TMEM40, TMEM45A, TMPPE, TMPRSS11E, TMPRSS11F, TMPRSS11GP, TMPRSS4, TMX2, TNIP2, TNPO2, TOP3A, TPT1, TRIM16L, TRIM25, TRMT9B, TSGA10, TSPAN31, TSTD2, TTN, TUBGCP2, TUSC1, TYSND1, U2AF2, UACA, UBALD2, UBE2H, UBE2Z, UBOX5-AS1, UBQLN4, UBXN4, UBXN7, UNC119B, UNC45A, UNC5CL, UPF3A, UPF3B, UPK1A, UPP1, UQCRC2, UQCRQ, USF2, USP32, USP38, USP6, UXT, VAT1, VIM-AS1, VNN1, VPS35, WASHC1, WDR31, WDR60, WFDC5, WRNIP1, WTIP, XKR7, XPO5, XYLB, YBX3P1, YTHDC2, YWHAEP1, YWHAG, YWHAH, YY1AP1, ZBTB10, ZBTB22, ZBTB7A, ZC2HC1C, ZC3HC1, ZDHHC14, ZDHHC24, ZFAND6, ZFX-AS1, ZG16B, ZHX1-C8orf76, ZNF12, ZNF143, ZNF296, ZNF354C, ZNF524, ZNF569, ZNF619, ZNF705D, ZNF708, ZNF75A, ZNF790, ZNF839, ZNF84, ZNRD2 |
| yellowgreen | ACO2, AFF1, ATP5MC1, BAD, CBX8, CDKN2A, CHCHD4, CLSPN, CPNE1, CRIP1, CT70, CTTN, DCAF11, DCUN1D4, DDX56, DERA, DIAPH2, DNAJB12, DUOX2, E4F1, EEA1, EXOC3, F2RL2, FAR2, FBXL12, FCF1, FIP1L1, FNBP4, GALNT14, GTF2F1, GYPC, HAUS2, HHEX, HLA-DRA, HORMAD2-AS1, IDO2, IKBKG, ITGB2-AS1, KIF3C, KRT15, LINC01160, LINC02404, LMAN2L, LOC100996318, LOC101928988, LOC107984945, LOC107985909, LOC107986950, LOC107987044, LOC107987398, MACO1, MAP2K7, MEF2C-AS1, MOSPD3, MPHOSPH6, MRPL16, MRPL43, MRTFA, NCBP1, NCOA7, NDEL1, NDUFA8, NHLH1, NT5C, NUDT22, P2RY10, PGM3, PIGS, PLRG1, POLR2J2, POLR3C, POMC, RAB24, RAB2B, RACK1, RFC2, RPS27L, S100A16, SAP30L-AS1, SEL1L3, SIRPD, SLC12A8, SLC8B1, SMURF2P1-LRRC37BP1, SNHG20, SNHG6, SOX4, SP100, SPINT1, SPTSSA, SSB, SSBP2, STAMBPL1, STK19, TAGLN, TINAGL1, TMEM14C, TMEM44-AS1, TOM1L2, TPTEP1, TRIM52-AS1, TRIM9, UBA6-AS1, UQCC3, USF1, VPS72, WDR47, XPO6, XRN1, ZNF417 |
